# Radiation-response in primary fibroblasts of long-term survivors of childhood cancer with and without second primary neoplasms: the KiKme study

**DOI:** 10.1186/s10020-022-00520-6

**Published:** 2022-09-06

**Authors:** Caine Lucas Grandt, Lara Kim Brackmann, Alicia Poplawski, Heike Schwarz, Willempje Hummel-Bartenschlager, Thomas Hankeln, Christiane Kraemer, Federico Marini, Sebastian Zahnreich, Iris Schmitt, Philipp Drees, Johanna Mirsch, Desiree Grabow, Heinz Schmidberger, Harald Binder, Moritz Hess, Danuta Galetzka, Manuela Marron

**Affiliations:** 1 Leibniz Institute for Prevention Research and Epidemiology, BIPS, Achterstraße 30, 28359 Bremen, Germany; 2 Faculty of Human and Health Sciences, University of Bremen, Bremen, Germany; 3 Institute of Medical Biostatistics, Epidemiology and Informatics (IMBEI), University Medical Center of the Johannes Gutenberg University Mainz, Mainz, Germany; 4 Institute of Organismic and Molecular Evolution, Molecular Genetics and Genome Analysis, Johannes Gutenberg University Mainz, Mainz, Germany; 5 Department of Radiation Oncology and Radiation Therapy, University Medical Center of the Johannes Gutenberg University Mainz, Mainz, Germany; 6 Department of Orthopaedics and Traumatology, University Medical Center of the Johannes Gutenberg University Mainz, Mainz, Germany; 7 Radiation Biology and DNA Repair, Technical University of Darmstadt, Darmstadt, Germany; 8 Division of Childhood Cancer Epidemiology, German Childhood Cancer Registry, Institute of Medical Biostatistics, Epidemiology and Informatics (IMBEI), University Medical Center of the Johannes Gutenberg University Mainz, Mainz, Germany; 9 Institute of Medical Biometry and Statistics, Faculty of Medicine and Medical Center, University of Freiburg, Freiburg, Germany

**Keywords:** Pathway analysis, Differential gene expression, RNA-Seq, Radiation experiment, Radiation response, High dose, Low dose, NGS

## Abstract

**Background:**

The etiology and most risk factors for a sporadic first primary neoplasm in childhood or subsequent second primary neoplasms are still unknown. One established causal factor for therapy-associated second primary neoplasms is the exposure to ionizing radiation during radiation therapy as a mainstay of cancer treatment. Second primary neoplasms occur in 8% of all cancer survivors within 30 years after the first diagnosis in Germany, but the underlying factors for intrinsic susceptibilities have not yet been clarified. Thus, the purpose of this nested case–control study was the investigation and comparison of gene expression and affected pathways in primary fibroblasts of childhood cancer survivors with a first primary neoplasm only or with at least one subsequent second primary neoplasm, and controls without neoplasms after exposure to a low and a high dose of ionizing radiation.

**Methods:**

Primary fibroblasts were obtained from skin biopsies from 52 adult donors with a first primary neoplasm in childhood (N1), 52 with at least one additional primary neoplasm (N2+), as well as 52 without cancer (N0) from the KiKme study. Cultured fibroblasts were exposed to a high [2 Gray (Gy)] and a low dose (0.05 Gy) of X-rays. Messenger ribonucleic acid was extracted 4 h after exposure and Illumina-sequenced. Differentially expressed genes (DEGs) were computed using *limma* for R, selected at a false discovery rate level of 0.05, and further analyzed for pathway enrichment (right-tailed Fisher’s Exact Test) and (in-) activation (z ≥|2|) using *Ingenuity Pathway Analysis*.

**Results:**

After 0.05 Gy, least DEGs were found in N0 (n = 236), compared to N1 (n = 653) and N2+ (n = 694). The top DEGs with regard to the adjusted *p*-value were upregulated in fibroblasts across all donor groups (*SESN1*, *MDM2*, *CDKN1A*, *TIGAR*, *BTG2*, *BLOC1S2*, *PPM1D*, *PHLDB3*, *FBXO22*, *AEN*, *TRIAP1*, and *POLH)*. Here, we observed activation of ***p53 Signaling*** in N0 and to a lesser extent in N1, but not in N2+. Only in N0, DNA (excision-) repair (involved genes: *CDKN1A*, *PPM1D*, and *DDB2*) was predicted to be a downstream function, while molecular networks in N2+ were associated with cancer, as well as injury and abnormalities (among others, downregulation of *MSH6*, *CCNE2*, and *CHUK*). After 2 Gy, the number of DEGs was similar in fibroblasts of all donor groups and genes with the highest absolute log_2_ fold-change were upregulated throughout (*CDKN1A, TIGAR, HSPA4L*, *MDM2*, *BLOC1SD2*, *PPM1D*, *SESN1*, *BTG2*, *FBXO22*, *PCNA*, and *TRIAP1*). Here, the ***p53 Signaling****-*Pathway was activated in fibroblasts of all donor groups. The ***Mitotic Roles of Polo Like Kinase****-*Pathway was inactivated in N1 and N2+. ***Molecular Mechanisms of Cancer*** were affected in fibroblasts of all donor groups. *P53* was predicted to be an upstream regulator in fibroblasts of all donor groups and *E2F1* in N1 and N2+. Results of the downstream analysis were ***senescence*** in N0 and N2+, ***transformation of cells*** in N0, and no significant effects in N1. Seven genes were differentially expressed in reaction to 2 Gy dependent on the donor group (*LINC00601*, *COBLL1*, *SESN2*, *BIN3*, *TNFRSF10A*, *EEF1AKNMT*, and *BTG2*).

**Conclusion:**

Our results show dose-dependent differences in the radiation response between N1/N2+ and N0. While mechanisms against genotoxic stress were activated to the same extent after a high dose in all groups, the radiation response was impaired after a low dose in N1/N2+, suggesting an increased risk for adverse effects including carcinogenesis, particularly in N2+.

**Supplementary Information:**

The online version contains supplementary material available at 10.1186/s10020-022-00520-6.

## Introduction

Malignant neoplasms or any neoplasm in the central nervous system occurring in children and adolescents before the age of 20 years are defined as childhood cancer (IARC 2016). Despite extensive research on this topic, risk factors for sporadic childhood cancer remain largely unknown (Thun et al. 2017). Inherited genetic predispositions only account for 10% of childhood cancer cases (Saletta et al. 2015). Such predispositions can affect pathways that are needed for radiation-response in the course of treatment for the first primary neoplasm (Saletta et al. 2015; Vogelstein et al. 2004). Genotoxic cancer therapies using cytostatic drugs and ionizing radiation (IR) in high doses [HDIR, ≥ 2 Gray (Gy)] during radiation therapy are established risk factors for the development of second primary neoplasms later in life (Spector et al. 2015; Tukenova et al. 2011; Scholz-Kreisel et al. 2018; Travis et al. 2012; Ron et al. 1988; Inskip et al. 2016; Meadows et al. 1980; Tucker et al. 1991). Young age at exposure is an additional and important risk factor for iatrogenic second primary neoplasms (Hodgson et al. 2017). Of all survivors of a first primary neoplasm occurring before the age of 15 years in Germany, 8% develop a second primary neoplasm within 30 years of the first diagnosis (Scholz-Kreisel et al. 2018). Since the incidence and survival rates of childhood cancer are increasing worldwide, the number of second primary neoplasms is expected to increase as well (Scholz-Kreisel et al. 2018; Kutanzi et al. 2016; Kaatsch et al. 2017). Contrary to the established cancer risk associated with exposure to HDIR, the carcinogenic potential of low doses of IR (LDIR, < 0.1 Gy) is still being discussed and controversial (Inskip et al. 2016; Spycher et al. 2015; Cardarelli et al. 2018; Sutou 2018; Ji et al. 2019; Luckey 1982; Graupner et al. 2017; Ma et al. 2013; Velegzhaninov et al. 2015). In children, studies suggest elevated leukemia risk in association with diagnostic applications of LDIR, such as computed tomography (Berrington de Gonzalez et al. 2016; Little et al. 2018). Despite the potential risk not being fully understood, LDIR are increasingly used in medical diagnostics (Smith-Bindman et al. 2008; Linet et al. 2012). In Germany, diagnostic applications account for about 41% of total radiation deposited in organs per person and year (1.6 of 3.9 millisievert) (Claudia Hachenberger et al. 2016). Also, in radiation therapy, the unwanted but inevitable exposure to LDIR outside the primary radiation beam is regarded as an additional risk factor for second primary neoplasms besides the impact of HDIR deposited within the tumor volume (Diallo et al. 2009).

Irrespective its dosage, exposure to IR causes a variety of cellular responses primarily based on the potent induction of deoxyribonucleic acid (DNA) damage (Nikitaki et al. 2016). On the cellular level, radiation-induced DNA damage activates complex signaling cascades of DNA repair, cell cycle regulation, apoptosis, senescence, and immunogenic responses (Mavragani et al. 2016). The induction of these signaling pathways is dose-dependent (Graupner et al. 2017; Velegzhaninov et al. 2015; Ghandhi et al. 2015; Tilton et al. 2016; Helm et al. 2020), whereby signaling pathways for the inactivation of cells by apoptosis or premature senescence predominate after HDIR (Sokolov et al. 2015; Ding et al. 2005). Such mechanisms of radiation-response can be measured by changes in gene expression (Wahba et al. 2016) and ribonucleic acid (RNA)-sequencing has shown to be a potent and precise method for transcriptome analysis, replacing probe-based tools such as microarrays (Kukurba et al. 2015). Studies that examined the molecular response to IR via transcriptome analysis have been conducted using vastly different cell types, irradiation doses, time points of extraction of messenger RNA (mRNA), and sequencing-methods depending on their research intent (Sokolov et al. 2015; Ding et al. 2005; Ray et al. 2012; Yunis et al. 2012; Warters et al. 2009; Stecca et al. 1998). In a previous work, we have reported the downregulation of *Cell cycle checkpoint control protein RAD9A* (*RAD9A* and *Cyclin Dependent Kinase Inhibitor 1A* (*﻿CDKN1A*) in non-irradiated (Victor et al. 2013a) and irradiated [1 Gy, (Weis et al. 2011)] fibroblasts of former childhood cancer patients with at least one second primary neoplasm (N2+), compared to former childhood cancer patients with a first primary neoplasm only (N1). Both of these genes are associated with the cellular response and repair of radiation-induced DNA damages. These findings indicate a compromised radiation-response in N2+ that might be associated with an increased risk to their second primary malignancies. Additionally, we hypothesize that former childhood cancer patients show differences in the transcriptional radiation-response in their skin fibroblasts compared to those of cancer-free controls (N0). To date, changes in gene expression induced by IR have not been examined and results have not been compared between N1 and N2+ using next-generation sequencing and subsequent pathway analysis.

The present study aimed to unravel molecular mechanisms of susceptibilities to sporadic cancer in early childhood and therapy-associated second primary neoplasms related to the genotoxic radiation-response. For this purpose, we explored gene expression profiles using Illumina-based next-generation sequencing and *Ingenuity Pathway Analysis* (*IPA*) in N1 (n = 52), N2+ (n = 52), and N0 (n = 52) after exposure to a low and a high dose of IR.

## Design, participants, and methods

### Study design and participants

The KiKme study is a nested case–control study with emphasis on the interplay between hereditary dispositions, cellular reaction to IR, risk for sporadic childhood cancer, as well as therapy-related second primary neoplasms. From 2013 to 2019, 591 participants were included in the study. The study population, data collection, and strategies for recruitment were described elsewhere (Marron et al. 2021). For the experiments in this project, primary skin fibroblasts, obtained from skin biopsies of 156 participants from the KiKme-study, were used (Table 1). Former childhood cancer patients registered in the German Childhood Cancer Registry (Scholz-Kreisel et al. 2018) provided the basis for the recruitment of cancer patients (52 with at least one second primary neoplasm and 52 with first primary neoplasms only). Participants had to be at least 18 years old and diagnosed with one first primary neoplasm in childhood. The first primary neoplasm had to be one of the three most common childhood cancers, including leukemia, lymphoma, or a tumor of the central nervous system. The second primary neoplasm had to occur at an anatomic site potentially exposed during radiation therapy, such as thyroid carcinoma, breast cancer, skin carcinoma, malignant melanoma, leukemia, or ependymomas and choroid plexus tumors (Dracham et al. 2018). In addition, 52 cancer-free controls were recruited from patients undergoing an elective surgery unrelated to cancer at the Department of Orthopedic Surgery at the University Medical Center Mainz. One long-term childhood cancer survivor with at least one second primary neoplasm, one without a second primary neoplasm, and one cancer-free control were matched according to age at sampling, as well as sex, and then processed as triplets. The long-term cancer survivors of a triplet were also matched according to the tumor entity as well as the age at and year of diagnosis of the first primary neoplasm.

**Table 1:** Description of participants (N = 156; n = 52 per donor group)

	Donors with at least one second primary neoplasm	Donors with a first primary neoplasm	Cancer-free controls
Female, %	51.92		
Median age at sampling [Interquartile Range (IQR)], years	32.00 (28.00–38.20)	32.50 (28.00–38.20)	33.00 (27.80–38.00)
Median age at first neoplasm (IQR), years	8.00 (4.00–11.20)	8.00 (3.75–12.00)	–
Median age at second neoplasm (IQR), years	23.00 (17.00–30.00)	–	–
First neoplasm			
Hodgkin lymphomas	18	18	–
Lymphoid leukaemias	18	18	–
Intracranial and intraspinal embryonal tumours	5	5	–
Non-Hodgkin lymphomas (except Burkitt lymphoma)	4	4	–
Acute myeloid leukaemias	3	3	–
Chronic myeloproliferative diseases	1	1	–
Burkitt lymphoma	1	1	–
Ependymomas and choroid plexus tumour	1	1	–
Astrocytomas	1	1	–
Second neoplasm			
Skin carcinomas	25	–	–
Thyroid carcinomas	17	–	–
Acute myeloid leukaemias	3	–	–
Breast Cancer	2	–	–
Malignant melanomas	2	–	–
Lymphoid leukaemias	2	–	–
Ependymomas and choroid plexus tumour	1	–	–
Third neoplasm			
No third neoplasm	49	52	52
Other specified intracranial and intraspinal neoplasms	2	–	–
Renal carcinomas	1	–	–

### Samples and experiments

**Fig. 1:**
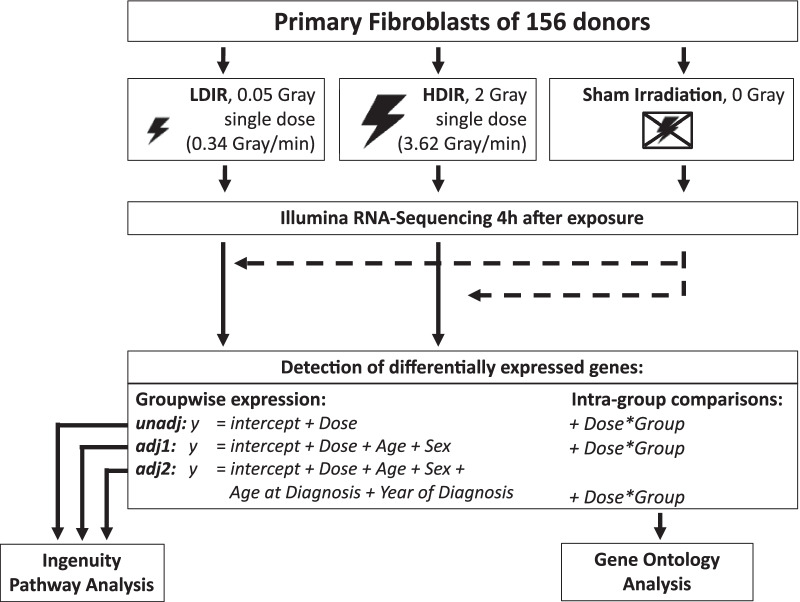
**Experimental workflow.** Primary fibroblasts of all donors were processed as matched triplets consisting of a donor with a first primary neoplasm only, a donor with a second primary neoplasm and a cancer-free control. The triplets were exposed to a low dose of ionizing radiation (LDIR, 0.05 Gray), a high dose of ionizing radiation (HDIR, 2 Gray), or were sham-irradiated (0 Gray). mRNA was extracted 4 h after exposure, Illumina-sequenced, and processed for the investigation of differentially expressed genes for each donor with regard to the regulation/level in sham-irradiated cells (dashed arrows). Differentially expressed genes as a result of groupwise-expression models were further subjected to *Ingenuity Pathway Analysis* and resulting gene-sets of the intra-group comparisons were analyzed for over-representation using the Gene Ontology via ConsensusPathDB

The acquisition of biosamples, the establishment of primary fibroblasts, radiation experiments, RNA extraction and processing, as well as the analysis for differentially expressed genes (DEGs) have been described in depth elsewhere (Marron et al. 2021; Brackmann et al. 2020). In summary, fibroblasts were obtained via skin biopsies from former childhood cancer patients as a 3 mm punch biopsy from the inside of the cubital region. Samples from cancer-free controls were taken from the scar region of the surgery. Primary fibroblasts were expanded from biopsies for 14–15 days and cryopreserved. For radiation experiments, cells were thawed, cultured, and synchronized in G1 by confluency. An overview of the experimental workflow is provided as Fig. 1. Cells were exposed at room temperature to a dose of 2 Gy with 140 kilovolt X-rays at a dose rate of 3.62 Gy per minute or to 0.05 Gy with 50 kilovolt X-rays at a dose rate of 0.34 Gy per minute using the D3150 X-ray Therapy System (Gulmay Medical Ltd, Byfleet, UK). This was achieved by reducing voltage to 50 kilovolt and a source-to-target distance of 30 cm. Thus, exposure times were 0.55 and 0.15 min for HDIR and LDIR, respectively. Sham-irradiated cells were kept at the same conditions in the radiation device control room. Cells from matched triplets were cultivated and treated simultaneously to prevent batch effects within groups. mRNA was extracted four hours after radiation exposure. In previous work, we showed the highest number of DEGs in fibroblasts at this time point during an overall observational period of 15 min to 24 h postexposure to IR (Brackmann et al. 2020). For mRNA-sequencing (RNA-Seq), libraries were processed on a HiSeq2500 instrument (Illumina, San Diego, California, USA) set to high-output mode. Single-end reads with a length of 50 base pairs were generated using *TruSeq Single Read Cluster Kit v3* (Illumina, San Diego, California, USA) and *TruSeq SBS Kit v3* (Illumina, San Diego, California, USA). Base calling was performed by *Real-Time Analysis* (Version 1.8.4) and the resulting data were converted into FASTQ format using *bcl2fastq* (Version 1.8.4, Illumina, San Diego, California, USA).

### Processing of RNA-Seq data

To detect DEGs and conduct pathway analysis, RNA-Seq data were processed (Marron et al. 2021). First, *Trimmomatic* was employed to clean adapter sequences from the raw reads (Bolger et al. 2014). Here, a quality of less than 3 was defined as the threshold to remove bases. The reads with an average quality less than 15 over 4 bases were trimmed. Processed reads were aligned to the human reference genome (GRCh38) using *STAR* [STAR_2.6.0c, Dobin et al. 2013]. The number of aligned reads per gene (seen as equal to the expression per gene), was quantified using *FeatureCounts* (Liao et al. 2014). Data were then normalized using the *voom* method (Law et al. 2014).

### Analysis of differential gene expression

DEGs dependent on radiation dose were detected using linear models implemented in the *limma* package (Ritchie et al. 2015), accounting for the individual donor as block variable, as well as the factors *donor group* and *radiation dose*. The differential gene expression after irradiation was computed by comparing measurements of fibroblasts of each participant with measurements after sham-irradiation (e.g., counts of transcripts in cells of each individual after 0 Gy versus counts after 2 Gy; Additional files 1, 2). Differential expression was detected using three different models: (1) crude model, (2) considering age and sex (model 1), and (3) considering age, sex, age at and year of diagnosis of the first neoplasm, and tumor type (model 2). The latter was used in analysis with N1 and N2+ data only. *P*-values were computed for the interaction between the effect of radiation and donor group, as well as the effect of radiation alone. Genes with a *p*-value adjusted at a false discovery rate [FDR, Benjamini–Hochberg procedure (Benjamini et al. 1995)] below 0.05 were flagged as significant for further analyses. Since multiple pair-wise comparisons were calculated, *p*-values from the separate analysis for each donor group have to be regarded as explorative.

### Quantitative real-time reverse-transcriptase polymerase chain reaction (qRT-PCR)

Total RNAs were prepared from treated and untreated fibroblast cultures using the Nucleo Spin RNA Plus Kit from Macherey–Nagel (Düren, Germany). 2 µg of the RNA samples were reversely transcribed into complementary DNA using the SuperScript IV First-Strand random hexamer Synthesis System (Invitrogen, Waltham, United States). Exon-spanning forward and reverse primers were designed with Primer-BLAST (Ye et al. 2012) or purchased from Qiagen (Hilden, Germany), depending on the availability of primers at Qiagen (Additional file 3a). Transcripts of the *TATA-Box Binding Protein* gene were used as endogenous reference gene control. Each 10 µl reaction volume contained 25 ng complementary DNA or DNA template in 5 µl Sybr-Green Master Mix (Biozym), 2 µl ribonuclease-free PCR graded water (MN), and 1 µl each of forward and reverse primer (10 µM). All reactions were performed in triplicate and two stages, with one cycle of 95 °C for 10 min (first stage) and 45 cycles of 94 °C for 10 s, 10 s at the primer-specific melting temperature, and 72 °C for 10 s (second stage) using the LightCycler 480II Roche. Amplification qualities were assayed using melting curves and agarose gel analysis. The qRT-PCR amplification efficiency was calculated using the LinReg program (Ruijter et al. 2009) and the cycle threshold values were corrected using the mean amplification efficiency. Relative quantification was carried out with the comparative cycle threshold method using the endogenous control gene and the control at 0 Gy for calibration.

### Ingenuity pathway and gene ontology analysis

Pathway analyses were conducted via *IPA* [Content Version 57662101, QIAGEN Inc., 2020, (Krämer et al. 2014)]. As input, lists of DEGs and the corresponding gene-wise *p*-values (adjusted with the FDR procedure), as well as the log_2_ fold-change (LFC) retrieved from the analysis of differential expression were used. The cutoff for the FDR was set to 0.05. The settings for *IPA* were selected as *experimentally observed data in human fibroblasts and commercial fibroblast cell lines* (Additional file 4). In *IPA*, Fisher’s Exact Test is employed to compute the significance of the overlap of a list of DEGs and a gene list, representing a pathway. Negative logarithms (-log) of the resulting *p*-values are reported by *IPA* and a value of at least 1.3 (≙ *p*-value = 0.05) after Benjamini–Hochberg correction was regarded as *significant* here. Such pathways were marked as *affected*. (In-) activation z-score threshold was set to z ≥|2| (Krämer et al. 2014). The z-score indicates pathway activation or inactivation by comparing given expressional directions of pathway components with information from the data set entered for analysis. If z ≥|2|, a pathway was marked as *modulated*. Moreover, pathways with a negative z-score ≤ −2 were marked as *inactivated*, pathways with a z-score ≥ 2 were marked as *activated*. The ratio, calculated as the number of DEGs (k) per number of postulated genes in the pathway in *IPA* database (K), was documented as well. For some pathways, z-score calculation was not possible [z = Not a number (NaN)]. This was either because of insufficient data in the *IPA* database (lack of information on “expected direction of differential expression”) or the information provided by our data was not sufficient. For these pathways, either activity prediction is not useful due to its circular or multi-cellular nature, or data are not sufficient for proper calculation of the z-score. The complete output from the *IPA* analyses can be found in Additional file 5. For all experiments, molecular networks from *IPA* were extracted. For the sake of legibility, only networks with a network score > 10 were included into the main body of this work, as all data sets showed only 1–2 networks with a score of this magnitude, with the next networks per donor group and dose combination reaching scores in the range of 1–6. The full list can be found in Additional file 5d. *IPA* was also employed to predict upstream regulators. These are transcription factors that are likely to have induced the DEGs and the direction of differential expression. Again, Fisher’s Exact Test was used to calculate a *p*-value for the significance of overlap of DEGs and genes, known to be regulated by a specific transcription factor. As described above, a z-score for the prediction of the activation state was calculated where applicable by *IPA*. This method was also applied for the prediction of downstream functions and diseases (Additional file 5c). As with pathway analyses, results from upstream and downstream analyses were adjusted using the Benjamini–Hochberg procedure to adjust for FDR. All results of the pathway analyses for model 1 are displayed in heat maps in Additional file 6. A comparison of differential expression values of genes per donor group and treatment in the analyzed pathways was also plotted as heat maps (Additional file 7). In all analyzed pathways, the molecule activity prediction of *IPA* was used (Additional file 8). Moreover, genes found in the *IPA* network analysis were displayed in a heat map, as well (Additional file 9). To account for even small effects in the interaction of radiation and donor group, results of the interaction analysis were filtered for the top 50 genes per donor group comparison with regard to the *p*-value irrespective of the significance threshold. These gene sets were then analyzed for over-representation using the ConsensusPathDB [Additional file 5e, (Kamburov et al. 2012)] against the gene background provided in Additional file 5f. The resulting Gene Ontology (GO) terms were then summarized as well as displayed in tree maps using REVIGO (Supek et al. 2011) with an allowed semantic similarity of 0.9 and organism set to *Homo sapiens* (Additional file 10a and b). Moreover, we filtered the genes per comparison for those within the 0.01% lowest *p*-values of each respective data set, a *p*-value < 0.15, or LFC >|0.75|. For additional sensitivity analyses, DEGs and subsequent *IPA* analyses were also computed for data (1) stratified by sex and adjusted for age (Additional file 11) and (2) after exclusion of self-reported non-Caucasian participants (n = 1) prior to computing DEGs using model 1 (Additional file 12). In addition to that, we repeated the *IPA* analyses using only genes showing absolute changes larger than 20% compared to the expression levels after 0 Gy (Additional file 13).

## Results

### Study participants

Primary fibroblasts of 156 donors were analyzed (52 N0, 52 N1, and 52 N2+; Table 1). The median age of participants was 32.0 and 32.5 years at sampling for long-term survivors of childhood cancer with at least one second primary neoplasm and without, respectively (interquartile range (IQR): 28.0–38.2 years) and 33.0 years (IQR: 27.8–38.0 years) for cancer-free controls. In each donor group, 27 (51.92%) of the participants were female. For long-term childhood cancer survivors, the median age at diagnosis for the first primary neoplasm was 8.0 years (IQR_second primary neoplasm_: 4.0–11.2 years, IQR_first primary neoplasm_: 3.8–12.0 years). The median age at diagnosis for the second primary neoplasm was 23.0 years (IQR: 17.0–30.0 years).

### Differential gene expression after exposure to LDIR

**Fig. 2:**
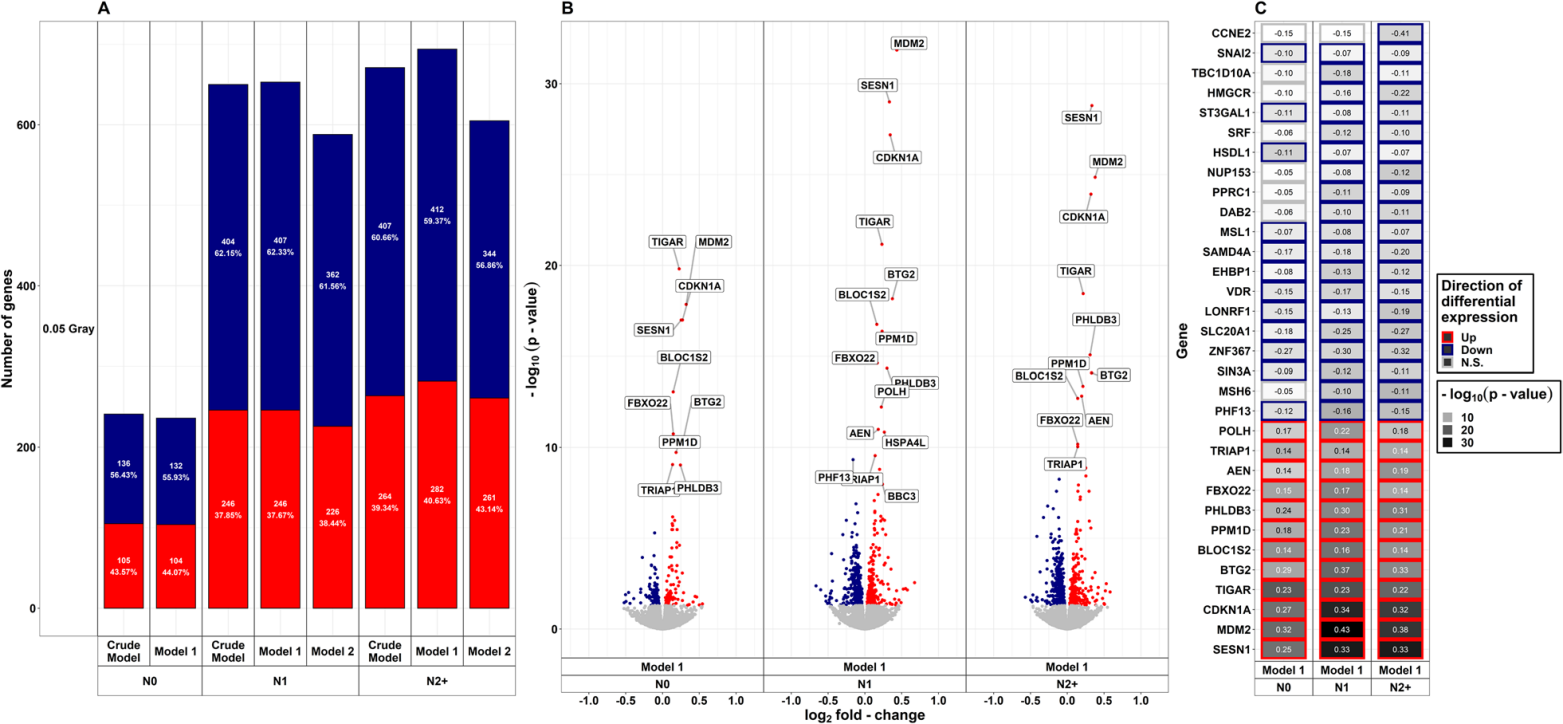
**Summarized results on differential gene expression after 0.05 Gray.** Differentially expressed genes in irradiated compared to sham-irradiated fibroblasts from donors with a first primary neoplasm only (N1), donors with at least one second primary neoplasm (N2+), and cancer-free controls (N0) 4 h after exposure to 0.05 Gray (false discovery rate adjusted *p*-value < 0.05). The data are presented for the crude model, model 1 (considering age at sampling and sex), and model 2 [considering age at sampling, sex, age at and year of diagnosis of the first neoplasm, and tumor type (not applicable for N0 data)]. In total, 14,756 genes were detected in the samples. Shown are **A** the proportion of up- and downregulated genes stratified by dose, group, and model, **B** volcano plots for the results of model 1, and **C** top 10 genes with regard to *p*-value, stratified by direction of log_2_ fold-change

In fibroblasts of all donor groups and for all adjustment models (crude model/model 1/model 2), more genes were down- than upregulated after exposure to LDIR (Fig. 2a). The total number of DEGs was about three times higher in both, N2+ (model 1: 694) and N1 (model 1: 653), compared to N0 (model 1: 236). A detailed list of DEGs after LDIR is provided in Additional file 1a. The amount of DEGs was similar across adjusted models for each group of donors. Less than half of all DEGs were upregulated across all three groups in all adjustment models in reaction to LDIR (Fig. 2a). N0 showed the highest fraction of upregulated genes (44.07%, model 1), albeit the lowest amount (n = 104, model 1) of total upregulated genes, followed by N2+ (40.63%, model 1), and N1 (37.67%, model 1) after LDIR. Compared to female N1 and N2+ and male N0, male N1 and N2+ showed a larger number of DEGs after LDIR (Additional file 11a). These were mostly centered at the *p*-value threshold of significance. The top genes with regard to the *p*-value in the main analysis were differentially expressed in all groups nevertheless (Additional file 11b). The removal of self-reported non-Caucasian participants did not change the results of the differential expression analysis after LDIR (Additional file 12a). Moreover, upregulated genes after LDIR showed higher peaks concerning the -log(*p*-value) in the volcano plots (Fig. 2b). Only upregulated genes showed a *p*-value < 10^–10^ across donor groups after LDIR. The top 10 genes with regard to *p*-value after LDIR are shown stratified by donor group and LFC-direction in Fig. 2c. All of the highest-ranking upregulated genes were present in all groups after LDIR. Across these top-ranking genes that were downregulated, *mutS homolog 6* (*MSH6*), *Disabled homolog 2* (*DAB2*), *Peroxisome proliferator-activated receptor gamma coactivator-related protein 1* (*PPRC1*), *Nucleoporin 153* (*NUP*153*)*, *Serum response factor* (*SRF*), *3-hydroxy-3-methyl-glutaryl-coenzyme A reductase* (*HMGCR*), and *TBC1 Domain Family Member 10A* (*TBC1D10A*) were observed to be only downregulated in N1 and N2+. *Cyclin E2* (*CCNE2*) was only downregulated in N2+. Nevertheless, qPCR conducted on *MSH6* for a subsample (n = 6 per group) did not confirm differential expression after LDIR (Additional file 3b). In a previous work within the collective of the KiKme study [n = 2 per group, (Brackmann et al. 2020)], we showed that across groups *Mouse double minute 2 (MDM2*) is upregulated after LDIR (Additional file 3c).

**Fig. 3:**
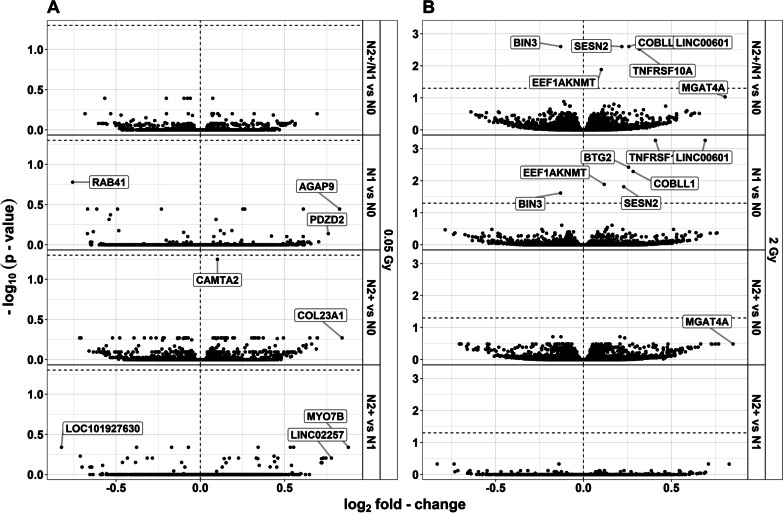
**Summarized results on interaction effects.** Results of analysis for differentially expressed genes in irradiated compared to sham-irradiated fibroblasts from donors with a first primary neoplasm only (N1), donors with at least one second primary neoplasm (N2+), and cancer-free controls (N0) 4 h after exposure to **A** 0.05 Gray and **B** 2 Gray including the interaction of group status and radiation dose. The data are presented for model 1 (considering age at sampling and sex). The highlighted genes were selected based on the data structure of each comparison, to highlight top-ranking genes: if their *p*-value was among the top 0.01% of that data set, p-value < 0.15, and/or log_2_ fold-change> |0.75|. Dashed horizontal lines indicate the significance threshold of the adjusted *p*-value < 0.05

Analysis for interactions of the effect of radiation dose and donor group was used to identify genes showing a differential reaction to radiation exposure between donor groups. Only one gene showed a differential reaction after exposure to LDIR: Comparing N2+ and N0, *Calmodulin binding transcription activator 2* (*CAMTA2*) was upregulated with a borderline significant *p*-value [LFC: 0.101, *p*-value = 0.056 (crude model)] after LDIR (Table 2). All genes that were differentially expressed after LDIR in more than one donor group showed the same direction of differential expression (Additional files 1b and 2b). Comparing fibroblasts of cancer survivors with N0, *member RAS oncogene family* (*RAB41*), *CAMTA2*, *POZ/BTB and AT hook containing zinc finger 1* (*PATZ1*), *receptor interacting serine/threonine kinase 1* (*RIPK1*), *speedy/RINGO cell cycle regulator family member E3* (*SPDYE3*), and *zinc finger protein 226* (*ZNF226*) were of interest based on the extended criteria after LDIR (Table 2, Fig. 3a). Comparing N1 with N2+, nine genes of interest were observed after LDIR: *ADNP antisense RNA 1* (*ADNP-AS1*), *ALG9 alpha-1,2-mannosyltransferase* (*ALG9*), *clustered mitochondria homolog pseudogene 3* (*CLUHP3*), *deleted in lymphocytic leukemia 2* (*DLEU2*), uncharacterized *LOC101927630*, *myosin VIIB* (*MYO7B*), *transcription factor CP2* (*TFCP2*), *Transforming growth factor beta-induced anti-apoptotic factor 1* (*TIAF1*), and *long intergenic non-protein coding RNA 2257* (*LINC02257*). Due to multiple occurrences of identical *p*-values, filtering for the top 50 genes with regard to the *p*-value per donor group comparison for the use in GO enrichment analysis resulted in more than 50 genes in two instances (n = 82 for N1/N2+ vs. N0 and n = 55 for N1 vs. N0). All of these were used in the GO analyses.

**Table 2:** Overview of genes for the analysis of interactions between the effect of the radiation dose and the donor group

	Gene	Genename	N2+ /N1 vs. N0		N1 vs. N0		N2+ vs. N0		N2+ vs. N1	
			FDR	LFC	FDR	LFC	FDR	LFC	FDR	LFC
0.05 Gray	*RAB41*	RAB41, member RAS oncogene family	0.404	− 0.567	0.166	− 0.757	–	–	–	–
	*CAMTA2*	Calmodulin binding transcription activator 2	0.404	0.074	–	–	0.056	0.101	-	-
	*PATZ1*	POZ/BTB and AT hook containing zinc finger 1	0.404	− 0.077	–	–	–	–	–	–
	*RIPK1*	Receptor interacting serine/threonine kinase 1	0.404	− 0.060	–	–	–	–	–	–
	*SPDYE3*	Speedy/RINGO cell cycle regulator family member E3	0.404	− 0.202	–	–	–	–	–	–
	*ZNF226*	Zinc finger protein 226	0.404	− 0.097	–	–	–	–	–	–
	*AGAP9*	ArfGAP with GTPase domain, ankyrin repeat and PH domain 9	–	–	0.359	0.826	–	–	–	–
	*PDZD2*	PDZ domain containing 2	–	–	0.727	0.758	–	–	–	–
	*COL23A1*	Collagen type XXIII alpha 1 chain	–	–	-	–	0.536	0.840	–	–
	*ADNP-AS1*	ADNP antisense RNA 1	–	–	–	–	–	–	0.457	0.533
	*ALG9*	ALG9 alpha-1,2-mannosyltransferase	–	–	–	–	–	–	0.457	-0.171
	*CLUHP3*	Clustered mitochondria homolog pseudogene 3	–	–	–	–	–	–	0.457	0.553
	*DLEU2*	Deleted in lymphocytic leukemia 2	–	–	–	–	–	–	0.457	-0.378
	*LOC101927630*	Uncharacterized LOC101927630	–	–	–	–	–	–	0.457	-0.824
	*MYO7B*	Myosin VIIB	–	–	–	–	–	–	0.457	0.877
	*TFCP2*	Transcription factor CP2	–	–	–	–	–	–	0.457	-0.071
	*TIAF1*	TGFB1-induced anti-apoptotic factor 1	–	–	–	–	–	–	0.457	0.342
	*LINC02257*	Long intergenic non-protein coding RNA 2257	–	–	–	–	–	–	0.624	0.777
2 Gray	***LINC00601***	**Long intergenic non-protein coding RNA 601**	**0.003**	**0.552**	**0.001**	**0.692**	–	–	–	–
	***COBLL1***	**Cordon-bleu WH2 repeat protein like 1**	**0.003**	**0.258**	**0.005**	**0.282**	–	–	–	–
	***SESN2***	**Sestrin 2**	**0.003**	**0.218**	**0.015**	**0.229**	0.193	0.208	–	–
	***BIN3***	**Bridging integrator 3**	**0.003**	−**0.130**	**0.024**	**− 0.131**	0.193	-0.128	–	–
	***TNFRSF10A***	**TNF receptor superfamily member 10a**	**0.003**	**0.317**	**0.001**	**0.409**	–	–	–	–
	***EEF1AKNMT***	**eEF1A lysine and N-terminal methyltransferase**	**0.013**	**0.101**	**0.013**	**0.118**	–	–	–	–
	*MGAT4A*	alpha-1,3-mannosyl-glycoprotein 4-beta-N-acetylglucosaminyltransferase A	0.093	0.805	0.427	0.760	0.324	0.851	–	–
	*CTPS2*	CTP synthase 2	0.130	− 0.113	–	–	–	–	–	–
	***BTG2***	**BTG anti-proliferation factor 2**	0.157	0.173	**0.004**	**0.256**	–	–	–	–
	*ZNF598*	Zinc finger protein 598	0.157	− 0.105	–	–	–	–	–	–
	*IP6K3*	Inositol hexakisphosphate kinase 3	–	–	0.339	− 0.787	–	–	–	–
	*PDZD2*	PDZ domain containing 2	–	–	0.434	0.755	–	–	–	–
	*ADAMTS17*	ADAM metallopeptidase with thrombospondin type 1 motif 17	–	–	–	–	–	–	0.469	− 0.833
	*DUOXA1*	Dual oxidase maturation factor 1	–	–	–	–	0.324	0.750	0.472	0.828
	*EDARADD*	EDAR associated death domain	–	–	–	–	0.324	0.769	–	–
	*HAS3*	Hyaluronan synthase 3	–	–	–	–	–	–	0.469	0.711
	*LOC100505622*	Uncharacterized LOC100505622	–	–	–	–	–	–	0.469	− 0.737
	*TSEN54*	tRNA splicing endonuclease subunit 54	–	–	–	–	0.193	− 0.175	–	–
	*ZNF2*	Zinc finger protein 2	–	–	–	–	–	–	0.469	− 0.140

Genes were selected if their *p-*value was among the top 0.01% of that data set, *p-*value < 0.15, or log2 fold-change >|0.75|

Data adjusted for age at sampling and sex (model 1) are shown. Genes with *p-*value < 0.05 adjusted for false discovery rate (FDR) are presented in bold text

N2+ = fibroblasts of donors with a first primary neoplasm in childhood and at least one second primary neoplasm, N1 = fibroblasts of donors with a first primary neoplasm in childhood, N0 = fibroblasts of cancer-free controls; LFC = log_2_ fold-change

### Functional analysis of DEGs after exposure to LDIR

The over-representation analysis resulted in distinct GO terms for all comparisons of interactions between radiation dose and donor group and can be found in Additional files 5e and 10b. Noteworthy, N2+ compared to N0 showed a large cluster of seven GO terms subsumed under the term ***tRNA methylation***. Comparing N1/N2+ with N0, ***stem cell differentiation*** and ***xenobiotic transport*** (including ***ion transport***) were the largest clusters of GO terms (three terms each).

**Fig. 4:**
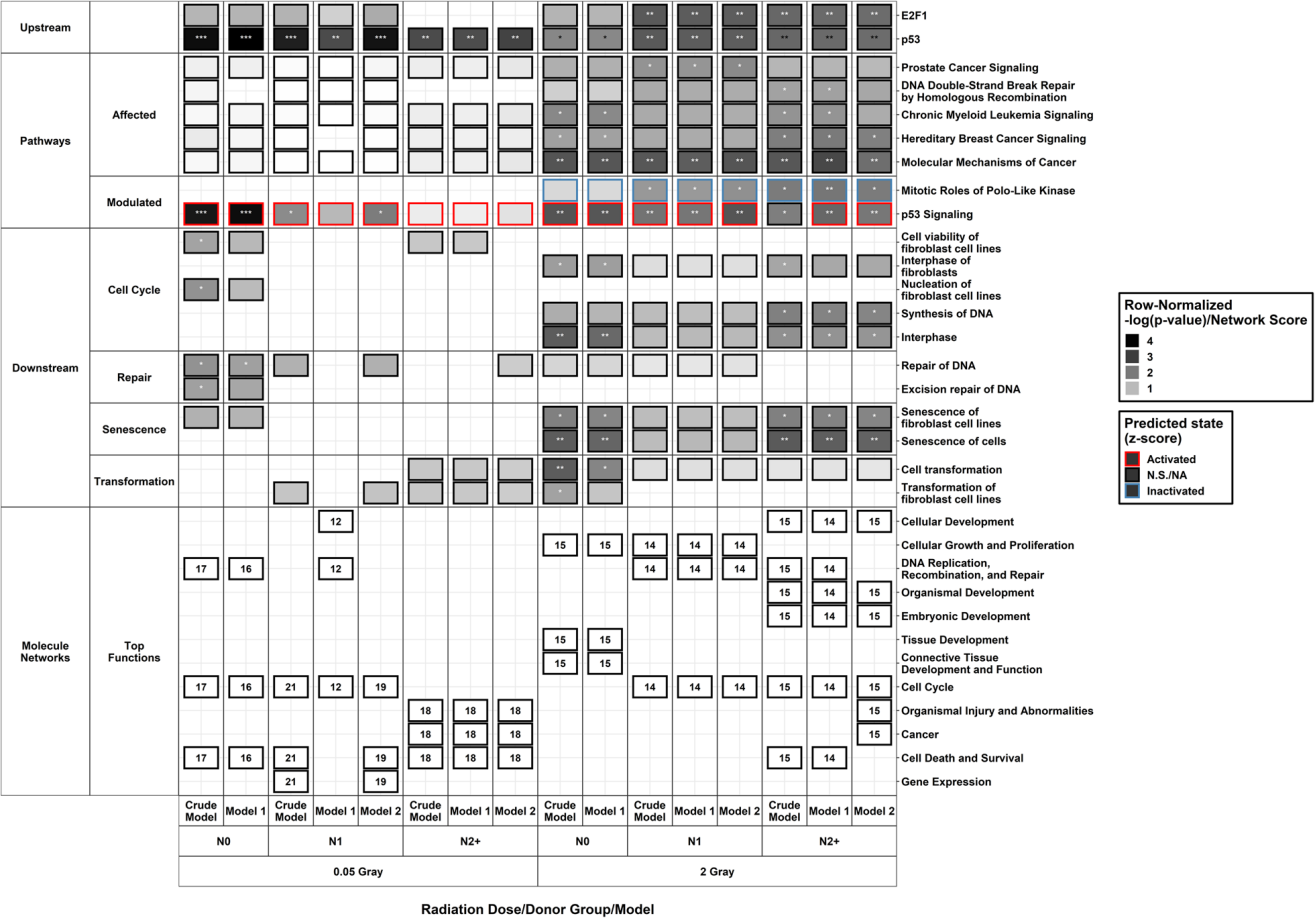
**Results of Ingenuity Pathway Analysis**. Overview of affected (false discovery rate adjusted at *p*-value < 0.05) and (in-) activated pathways (|z|≥ 2), predicted upstream effectors, downstream biofunctions and diseases, and observed molecular networks after irradiation with a low (0.05 Gray) or a high dose (2 Gray) ordered by *p*-value. For molecular networks, the network score instead of a *p*-value and no z-score was calculated by *Ingenuity Pathway Analysis* and is given inside the tiles. For each combination of dose, model, and donor group, the top functions of the network with a score > 10 is presented, except for N2+ after 2 Gray, where two networks had a score above 10 in all models. On the right-hand side, the functions associated with the networks are shown. *p-value < 0.05, **p-value < 0.01, ***p-value < 0.001. Model 1: considering age at sampling and sex, model 2: considering age at sampling, sex, age at and year of diagnosis of the first neoplasm, and tumor type (not used with data from N0), N0 = fibroblasts of cancer-free controls, N1 = fibroblasts of childhood-cancer survivors, N2+  = fibroblasts of childhood-cancer survivors with at least one second primary neoplasm

Data of DEGs per donor group, radiation dose, and adjustment model were used for upstream analyses in *IPA* (Fig. 4, Additional file 5b). We observed *Tumor protein p53* (*p53*) as a common predicted upstream regulator in fibroblasts of all donor groups after LDIR (Additional file 5a). N2+ and N0 showed *E2F* *Transcription Factor 1* (*E2F1*) to be an additional upstream regulator after LDIR, which was not significant after correction for false discovery.

Correspondingly, pathway analysis showed that ***p53 Signaling*** was affected and activated after LDIR, however only in N0 (crude model and model 1) and N1 (crude model and model 2), but not in N2+ (Fig. 4, Additional file 5b). Here, *Proliferating cell nuclear antigen (PCNA*) and *Tumor Protein P53 Inducible Nuclear Protein 1* (*TP53INP1*) were not differentially expressed in N2+, *P53-Induced Death Domain Protein 1* (*PIDD1*), and *B-cell lymphoma 2* (*BCL2*) only in N0 (Additional file 7b). Using only DEGs with an LFC higher than |0.25|, ***p53 Signaling*** was not activated in any donor group and only affected in all donor groups and models but N1, model 1 (Additional file 13). In this analysis, ***Cyclins and Cell Cycle Regulation*** and ***Estrogen-mediated S-phase Entry*** were inactivated in all models of N2+. In the sensitivity analysis ***Molecular Mechanisms of Cancer*** were only affected in N2+ after LDIR. Downstream functions in reaction to LDIR for N0 were predicted by *IPA* as well (Fig. 4, Additional file 5c). These were *Cell viability of fibroblast cell lines* [*CDKN1A*, *DNA Polymerase Eta* (*POLH*), and *Breast Cancer Gene 2, DNA repair associated* (*BRCA2*)]*, Nucleation of fibroblast cell lines* (*CDKN1A* and *BRCA2*)*, Excision repair of DNA* [*CDKN1A* and *DNA damage-binding protein 2* (*DDB2*)], and *Repair of DNA* (*CDKN1A*, *PPM1D*, and *DDB2*). Except the latter, all of these were only present in data of the crude model, whereas *Repair of DNA* was also found in model 1.

In each donor group and adjustment model, one network with a score over 10 was identified by *IPA* in the data from the LDIR treatment (Fig. 4, Additional files 5d and 9). The annotated functions of the network in N0 were *Cell Cycle*, *Cell Death and Survival*, and *DNA Replication, Recombination, and Repair* in the crude model and model 1. For N1, annotations in the crude model and model 2 were *Cell Cycle, Cell Death and Survival*, and *Gene Expression* after LDIR. In model 1, the network included *Cell Cycle, Cellular Development,* and *DNA Replication, Recombination, and Repair* after LDIR. For N2+, top diseases and functions were *Cancer*, *Cell Death and Survival*, and *Organismal Injury and Abnormalities* for all models. Together, we observed a trend towards the activation of cell cycle control and DNA repair in N0 and cell death- and cancer-associated signaling pathways in N2+ following LDIR.

### Differential gene expression after exposure to HDIR

**Fig. 5:**
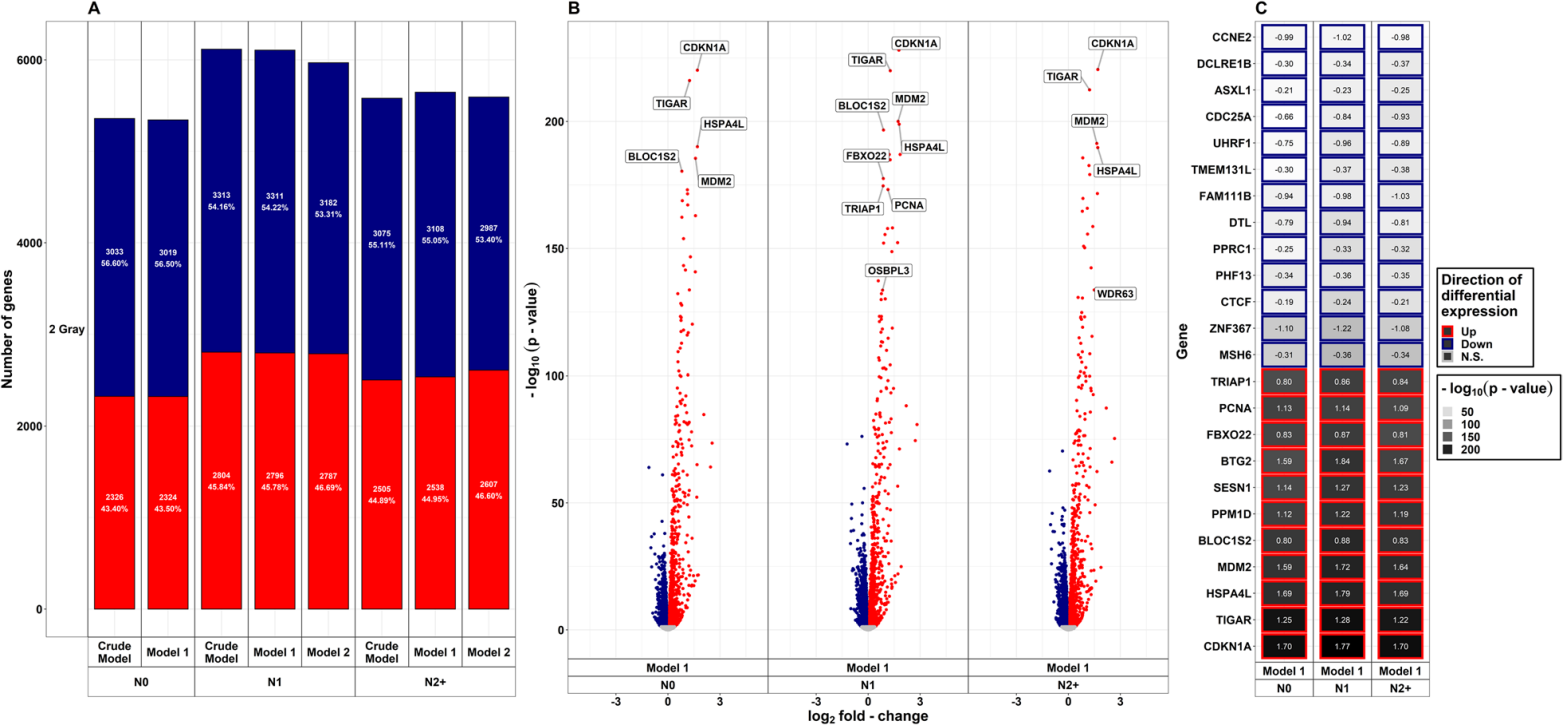
**Summarized results on differential gene expression after 2 Gray**. Differentially expressed genes in irradiated compared to sham-irradiated fibroblasts from donors with a first primary neoplasm only (N1), donors with at least one second primary neoplasm (N2+), and cancer-free controls (N0) 4 h after exposure to 2 Gray (false discovery rate adjusted *p*-value < 0.05). The data are presented for the crude model, model 1 (considering age at sampling and sex), and model 2 [considering age at sampling, sex, age at and year of diagnosis of the first neoplasm, and tumor type (not applicable for N0 data)]. In total, 14,756 genes were detected in the samples. Shown are **A** the proportion of up- and downregulated genes stratified by dose, group, and model, **B** volcano plots for the results of model 1, and **C** top 10 genes with regard to *p*-value, stratified by direction of log_2_ fold-change

As in treatment with LDIR, there were more downregulated than upregulated DEGs in fibroblasts of all donor groups after exposure to HDIR (Fig. 5a, Additional file 1a). The total number of DEGs was slightly higher in N2+ and N1 compared to N0 in all adjustment models and the number of DEGs per group differed only slightly between adjustment models after HDIR. As with reaction to LDIR, upregulated genes after HDIR showed lower *p-*values compared to downregulated genes (Fig. 5b, model 1 only). In detail, only upregulated genes showed a *p-*value in the range of 10^–75^ to 10^–275^ in all donor groups. All of the highest-ranking up- and downregulated genes were found in all groups after HDIR (Fig. 5c, model 1 only). Here, qRT-PCR conducted for *PCNA* for a subsample (n = 6 per group) did confirm differential expression after HDIR for N1 (Additional file 3b). Also, the upregulation of *CDKN1A* and *MDM2* across all groups in the present study has been previously confirmed by us using qRT-PCR [(Brackmann et al. 2020), Additional file 3c]. Neither stratifying for sex, nor the prior removal of self-reported non-Caucasian participants did change the results of the differential expression main analysis for exposure to HDIR. Contrary to the reaction to LDIR, several genes were differentially expressed when comparing the response to HDIR between donor groups (Table 2, Fig. 3b, Additional file 1b). *Bridging integrator 3* (*BIN3*), *cordon-bleu WH2 repeat protein like 1* (*COBLL1*), *eukaryotic translation elongation factor 1 alpha lysine and N-terminal methyltransferase* (*EEF1AKNMT*), *long intergenic non-protein coding RNA 601* (*LINC00601*), *sestrin 2* (*SESN2*), and *Tumor necrosis factor receptor superfamily member 10a* (*TNFRSF10A*) were differentially expressed comparing fibroblasts of both cancer groups to N0 after HDIR (model 1). Additionally, *BTG Anti-Proliferation Factor 2* (*BTG2*) was differentially expressed comparing N1 to N0 (model 1). As with the gene expression after LDIR, the general direction of the gene expression after HDIR in genes that were commonly differentially expressed in more than one donor group, was identical (Additional files 1b and 2b). The additional filtering for non-significant genes with a *p-*value smaller than 0.15, the 0.01% lowest *p-*values of the respective data, or with an LFC larger than |0.75|, found five genes of interest for the comparison of cancer survivor groups after HDIR: *ADAM metallopeptidase with thrombospondin type 1 motif 17* (*ADAMTS17*), *dual oxidase maturation factor 1* (*DUOXA1*), *hyaluronan synthase 13* (*HAS3*), the uncharacterized locus *LOC100505622,* and *zinc finger protein 2* (*ZNF2*) (Table 2, Fig. 3b).

### Functional analysis of DEGs after exposure to HDIR

Comparing reaction to HDIR between donor groups, analysis for GO term enrichment resulted in several group-specific differences (Additional files 5e and 10b﻿). Comparing the reaction of N0 to N1 and N2+ , the largest GO term clusters were ***regulation of signal transduction*** and ***DNA damage response signal transduction by p53 class mediator*** after HDIR. Comparing N0 and N1, the largest cluster (8 terms) was ***signal transduction in response to DNA damage*** and comparing N0 with N2+ the largest cluster (2 terms) was ***DNA methylation***. The comparison between the cancer groups (N1 and N2+) resulted in two GO terms (***transporter complex*** and ***cation channel complex***).

Fibroblasts of all donor groups had *p53* as a predicted upstream regulator in reaction to HDIR (Fig. 4, Additional file 5a) in the main analysis. Moreover, *E2F1* was predicted to be an upstream regulator in all adjustment models of N1 and N2+ after HDIR. In the sensitivity analysis using an LFC threshold of |0.25|, *p53* was predicted to be an active upstream regulator with a z-score > 2 for all models of N0 and N1, but not N2+ (Additional file 13). Additionally, *MYB Proto-Oncogene Like 2* (*MYBL2*), *Lin-9 homolog* (*LIN9*), Chromobox homolog 7 (*CBX7*), and *H2.0 Like Homeobox* (*HLX*) were inactivated, *Bromodomain Containing 7* (*BRD7*) was an activated upstream regulator for all donor groups and all models after HDIR, based on the *IPA* prediction. Only in all models of N2+ *Jun Proto-Oncogene* (*JUN*) was an active upstream regulator.

In the pathway analysis of the main analysis, ***p53 Signaling*** was found to be affected after HDIR. Contrary to LDIR, it was affected in all groups and models. Moreover, it was activated in all groups and models except N2+ (crude model) after HDIR. The ***Mitotic Role of Polo-Like Kinase pathway*** was inactivated in all donor groups, but not affected in any model for N0. Here, *STE20-like serine/threonine-protein kinase* (*SLK*) and *Checkpoint kinase 2* (*CHEK2*) were only downregulated in all models of N1 and N2+ (Additional file 7a). In the sensitivity analysis, ***p53 Signaling*** was activated in both models for N0 and affected in all models of N1 and N2+ (Additional file 13). Here, the ***Mitotic Role of Polo-Like Kinase*** pathway was inactivated in all models of all groups. Moreover, only in the sensitivity analysis, ***Cyclins and Cell Cycle Regulation***, ***Kinetochore Metaphase Signaling Pathway***, and ***Estrogen-mediated S-phase entry*** were inactivated in all models of all donor groups after HDIR. In the main analysis, there were five pathways where z-score calculation was not possible by *IPA* that were affected in at least one group. The ***Molecular Mechanisms of Cancer*** pathway was affected in all donor groups and models. ***Chronic Myeloid Leukemia Signaling*** was affected in N2+ (crude model and model 1), ***Hereditary Breast Cancer Signaling*** in all models of N0 and N2+ , ***Prostate Cancer Signaling*** in all models for N1, and ***DNA Double-Strand Break Repair by Homologous Recombination*** in N2+ (crude model and model 1) after HDIR.

In the analysis for downstream diseases and bio functions (Fig. 4, Additional file 5c), senescence (*senescence of cells* and *senescence of fibroblast cell lines*) showed a trend toward activation, *interphase* [(N0: *interphase* and *interphase of fibroblasts* (both, crude model and model 1), N2+ : *interphase* (all models) and *interphase of fibroblasts* (crude model)] toward inactivation after HDIR. In N0, transformation (*cell transformation* and *transformation of fibroblast cell lines*) showed a trend toward inactivation. *Synthesis of DNA* was found in N2+ (all models) with a trend toward inactivation in N0 and N2+ after HDIR. *Repair of DNA* was found to be a downstream function in N0 and N1, though not significant.

Following HDIR*,* associated functions for the network in N0 across all adjustments were *Cellular Development*, *Cellular Growth and Proliferation*, and *Connective Tissue Development and Function* (Fig. 4, Additional files 5d and 9). In N1, networks for both adjustment models showed associated functions to be *Cell Cycle*, *Cell Death and Survival* and *Cellular Development* after HDIR. In N2+, we observed two networks for model 1 and model 2. For both adjustment models, associated functions for the first respective network were *Cellular Development, Embryonic Development,* and *Organismal Development* in N2+. The second network in N2+ differed and was *Cell Cycle*, *Cell Death and Survival,* and *Cellular Development* for model 1 and *Cell Cycle, Cellular Development,* and *DNA Replication, Recombination, and Repair* for model 2.

## Discussion

In the present nested case–control study we examined and compared the gene expression and associated pathways in primary skin fibroblasts of childhood cancer survivors without or with a subsequent second primary neoplasm and of cancer-free controls after exposure to different doses of IR. Our results revealed alterations in the response to a low and a high dose of IR in fibroblasts of former childhood cancer patients compared to cancer-free controls. The *IPA*-analysis of upstream regulators, molecular networks, and predicted downstream-effects supported these findings and indicate impaired protection and higher vulnerability to the genotoxic impact of IR in former childhood cancer patients. In particular, compromised DNA repair and cell cycle-regulation may foster the development of therapy-associated second primary neoplasm already after exposure to low doses of radiation.

### Exposure to LDIR

***P53 Signaling*** is the most prominent pathway activated by genotoxic stress, e.g., shown after irradiation of whole blood with a variety of doses and dose rates (Ghandhi et al. 2015). Furthermore, the predicted downstream activities are in line with previous findings on the response to LDIR (Ding et al. 2005). In our data, activity prediction in N0 and N1 was similar: cell cycle progression, apoptosis, and DNA repair were increased with cell survival being inactivated.﻿ This pathway not being affected in any model for N2+ and being only weakly affected in N1 is an important finding for the personalized risk assessment of the application of LDIR in diagnostics.

Results from pathway analysis were further supported by the corresponding molecular networks and their attributed functions and diseases, where N0 and N1 had *DNA Repair* as associated function, while only N2+ included *Cancer*. After LDIR, a key molecule among others (*PGR, PCNA, TP53INP1 IL15, HSPA8, SOD1, CHUK, IRF1, CDC6, CCNE2*) in the network of N0 was *DDB2*, with important functions for (excision-) repair and cell fate decision (Sugasawa et al. 2019; Stoyanova et al. 2009). Additionally, the downstream functions *Excision Repair* and *Repair of DNA* were only found for N0. This implies, that if an insufficient or no apoptotic response occurs, induced genomic mutations may be transmitted to following generations carrying a potential carcinogenic risk by inducing resistance and increased DNA damage tolerance. Multiple exposures to LDIR could then amplify this effect (Martin et al. 2010).

We additionally hypothesize that the high difference in number of DEGs following LDIR between N0 and N1/N2+ may implicate an undirected reaction in N1 and N2+, including expression of genes without necessity for radiation-response, while N0 react in a more precise manner. Interestingly, after stratifying by sex, this was found only in male N1 and N2+, whereas the number of DEGs in female N1 and N2+ was similar to those in male and female N0 after LDIR. Sex-specific differences in radiation-response have been proposed before (Alsbeih et al. 2016). Nevertheless, further research is needed to elaborate on such differences in fibroblasts of childhood cancer survivors after LDIR.

### Exposure to HDIR

After HDIR, our most important finding was that ***p53 Signaling*** was affected and activated in all models of N1 and N0 but not activated in the crude model or N2+. When we applied a 20% LFC-threshold, only N0 showed activation of this pathway. The inactivation of the ***Mitotic Roles of Polo-Like Kinase*** pathway after HDIR in N1 and N2+ was in line with a radiation-induced G1 arrest as Polo-like kinases are, amongst others, responsible for G1/S-transition (Zitouni et al. 2014). A notable difference to N0 was the upregulation of *Heat shock protein 90-α* (*Hsp90α*) after HDIR in N2+ and N1 donors that has been previously described in various pathological conditions (Zuehlke et al. 2015) and stabilization of the Polo-like kinase 1. Future efforts are needed to investigate this signal in detail in order to validate our findings. ***Molecular Mechanisms of Cancer*** was affected in all donor groups after HDIR and cell survival was inactivated via the radiation-triggered activation of the *nuclear factor ‘kappa-light-chain-enhancer’ of activated B-cells* (*NF-kB*), a well-documented mechanism (Pordanjani et al. 2016). In N1 and N0, cell cycle progression was inactivated and apoptosis was activated after HDIR. Remarkably, we observed an inverse response to HDIR in N2+ that was mediated by the up-regulation of *RAC-alpha serine/threonine-protein kinase 1* (*AKT1*). The different isoforms of the proto-oncogene *AKT* promote radiation resistance by fostering DNA repair, survival, and proliferation (Sahlberg et al. 2014; Hollander et al. 2011). Here, activation of cell cycle progression and inactivation of apoptosis may lead to accumulation of DNA-damage that is subsequently carried on in further cellular generations.

### Comparison of the reactions to LDIR and HDIR

In reaction to LDIR, a limited subset of genes has been found to be consistently upregulated by other studies, like *CDKN1A* (Sokolov et al. 2015), which is activated in a *p53*-dependent manner and plays a pivotal role in cell cycle arrest (Sokolov et al. 2015; Maier et al. 2016). Kabacik et al. (2011) proposed a set of genes to be positively associated with the radiation response after 2, 4, and 24 h, following HDIR (2 and 4 Gy) in cultured lymphocytes and peripheral whole blood, among them *PCNA, Sestrin 1* (*SESN1)*, *Growth arrest and DNA-damage-inducible protein GADD45 alpha* (*GADD45A*), *CDKN1A*, *Cyclin G1* (*CCNG1)*, *Ferredoxin Reductase* (*FDXR*), *BCL2 Binding Component 3* (*BBC3*), and *MDM2*. The results for the upregulation of these genes after LDIR or HDIR were confirmed in the present work for all donor groups. Comparing the prediction of pathway-specific downstream activity of ***p53 Signaling***, results were similar between LDIR and HDIR: After both treatments, activation of apoptosis and downregulation of cell survival was inferred. However, *Cell Cycle Progression* was activated after LDIR and inactivated after HDIR, which may be rooted in the cellular effort to rather repair than induce apoptosis after the lower genotoxic insult that LDIR entails compared to HDIR.

### Interactions between radiation dose and donor groups

*CAMTA2*, the only gene with a borderline significant *p*-value when comparing group-specific differential expression in reaction to LDIR (comparing N2+ and N0, crude model) has been shown to be involved in cancer (Luan et al. 2019), stress reaction across species (Mollet et al. 2016), and cardiac hypertrophy (Song et al. 2006). The genes differentially expressed after HDIR (N2+ /N1 vs. N0 and N1 vs. N0) were associated to regulation of apoptosis [*TNFRSF10A*, (Pan et al. 1997)], survival in chronic lymphocytic leukemia [*COBLL1*, (Plešingerová et al. 2018; Plesingerova et al. 2017)], prevention of accumulation of reactive oxygen species [*SESN2*, (Wang, et al. 2019)], cell growth/proliferation, heat shock response, and tumorigenesis [*EEF1A1* (also known as *METTL13*), which is methylated by *EEF1AKNMT* (Vera et al. 2014; Liu et al. 2019)].

### Strengths and limitations

To the best of our knowledge, this is the first study that examined the genome-wide transcriptome response to IR in primary fibroblasts of a large group of former childhood cancer patients compared to cancer-free controls. Moreover, the participants were carefully matched for sex, age at sampling, as well as age at and type of first cancer diagnosis to account for the largest sources of variation. In addition, cases were matched by year of diagnosis to account for differences in treatment. Following our previous work, we used fibroblasts from skin biopsies, that were collected from a large, well-defined pool of cell donors [n = 156, (Marron et al. 2021)]. Unlike other studies, we chose to not use commercial cell lines (Velegzhaninov et al. 2015; Ding et al. 2005; Mezentsev et al. 2011; Hou et al. 2015; Ghandhi et al. 2008; Jen et al. 2005). Although commercial cell lines may be well researched, they do not adequately depict intrinsic individual biological variability. Therein also lies a limitation, as this unknown variation might have influenced our results. Our study controlled for this with the aforementioned careful matching of subjects, accounting for variation in the R software package *limma*, and adjustment of differential expression for factors that introduce most variation, such as age and sex. Despite other cell types being more accessible, such as lymphocytes via venipuncture, we chose fibroblasts to perform our radiation experiments on, because survival and cultivation of lymphocytes without immortalization by Epstein-Barr virus transformation is very limited (Neitzel 1986). Additionally, untransformed fibroblasts keep an intact cell cycle that may provide a more complete analysis of gene expression, even in confluency. Moreover, as some of our participating long-term childhood cancer survivors have received bone marrow transplants, blood samples would have contained foreign blood cells of the bone marrow donors (Victor et al. 2013b). This would have precluded analysis of germline mutations, which was our main aim. Finally, pathways were analyzed using *IPA*. This steadily updated and curated database allows the analysis of complex RNA-sequencing data. It gives insight beyond expression of single genomic features by incorporating measurements and inferring further information from the transcriptional patterns provided. Moreover, we conducted sensitivity analyses, such as the exclusion of self-reported non-Caucasian participants, which did not change the results of this study after LDIR or HDIR. This came unsurprisingly, as to date there is no data that reports on disparities in radiation-response based on ethnicity. Despite the strengths mentioned, important issues for radiation carcinogenesis such as systemic or non-targeted radiation effects like the bystander effect or genomic instability (Mavragani et al. 2016) were not addressed in our work. We aimed to provide initial results for future studies by using primary cells and performing radiation experiments in vitro. It was essential to examine basic cellular events before accounting for highly complex intercellular or systemic effects. As this is an important factor for radiation-related adverse effects and carcinogenesis, we aim to address these important issues in future works for tissues, cellular networks, and different cell types, with particular regard for an involvement of the immune response/system (Mavragani et al. 2016; El-Saghire et al. 2013). As the design of the study allowed only the inclusion of long-term cancer survivors, aggressive tumor entities with high mortality rates could not be taken into account. However, most cancers share constitutive characteristics, such as autonomous and unlimited proliferation, evasion of cell death, or invasion of normal tissues (Spector et al. 2015). This work investigated the impact of two different radiation doses at a single time point post-exposure. A low and a high dose were chosen to simulate clinically relevant exposure during radiologic examinations or radiation therapy, respectively (Pearce et al. 2012; Seidlitz et al. 2017). Even though gene expression patterns after LDIR show a dynamic transient response within 24 h post-radiation (Albrecht et al. 2012; Berglund et al. 2008), we (Brackmann et al. 2020) and others (Yunis et al. 2012; Chaudhry et al. 2012) observed the highest amount of DEGSs at 3–4 h after irradiation compared to sham-irradiated cells and we hence chose this time point of analysis for our investigations. Although dose rate effects at the transcriptome level are known for radiation exposures, such studies usually compare acute and very protracted irradiations. The latter is usually performed at dose rates of a few mGy per minute for several hours until the desired cumulative dose is reached. In the present study, however, both exposure scenarios with very short irradiation times represent acute exposures. Thus, the difference in absolute dose is expected to dominate the impact on the transcriptome, but the influence of varying dose rates between HDIR and LDIR cannot be ruled out. In irradiation experiments with human lymphocytes, different dose rates (1.03 Gy/min vs. 3.1 Gy/min) had no effect on activation of ***p53 signaling*** at the same cumulative doses (Ghandhi et al. 2015).

## Conclusion

In this study, we report alterations in the radiation-response in primary fibroblasts of former childhood cancer patients compared to cancer-free donors that were most pronounced in reaction to a low dose of IR in long-term survivors which developed a subsequent second primary neoplasm. Mechanisms directed against genotoxic stress were activated to the same extent after a high dose in all donor groups. However, the radiation response was impaired after a low dose in fibroblast of childhood cancer survivors. This suggests an increased risk for adverse effects by accumulation of DNA-damage following genotoxic insult that may subsequently lead to carcinogenesis, particularly in fibroblasts of donors with at least one second primary neoplasm. This work provides the foundation for further projects and facilitates guided validatory works on the proposed differences in molecular mechanisms coping with IR.

## Supplementary Information

**Additional file 1. **All results of the differential gene expression analyses. AF1a. Complete list of differentially expressed genes. Model 1 considers age at sampling and sex; model 2 considers age at sampling, sex, age at and year of first diagnosis, and tumor type (not used with data from cancer-free controls). Abbreviations: Gy = Gray, N0 = fibroblasts of cancer-free controls, N1 = fibroblasts of childhood-cancer survivors, N2+  = fibroblasts of childhood-cancer survivors with at least one second primary neoplasm, FDR = *p-value* adjusted for false discovery rate. AF1b. Differential expression computed with the interaction of radiation and donor group. The comparison-column refers to the donor groups that were compared in the analysis for interactions of the effect of radiation dose and donor group to identify genes showing a differential reaction to radiation exposure between donor groups. Abbreviations: Gy = Gray, N0 = fibroblasts of cancer-free controls, N1 = fibroblasts of childhood-cancer survivors, N2+  = fibroblasts of childhood-cancer survivors with at least one second primary neoplasm, FDR = *p*-value adjusted for false discovery rate.

**Additional file 2.** Comparisons of FDR and LFC across donor groups. Comparison of differential expression (DE) and its direction between donor groups.

**Additional file 3.** Primer for and results of qRT-PCR. AF3a. Constructed and purchased primer sequences for the qPCR. AF3b. Log_10_ relative expression measured in quantitative polymerase reverse transcriptase chain reaction (qRT-PCR) for selected genes compared to *TATA-Box Binding Protein* (TBP), stratified by group (each n = 6, three replicates per individuals) and radiation dose. Significance stars imply the *p*-value thresholds of two-sided t-tests after adjustment for false discovery rate (* *p*-value < 0.05, ** *p*-value < 0.01, *** *p*-value < 0.001). N0 = fibroblasts of cancer-free controls, N1 = fibroblasts of childhood-cancer survivors, N2+  = fibroblasts of childhood-cancer survivors with at least one second primary neoplasm. AF3c. Log_10_-relative expression measured in quantitative polymerase reverse transcriptase chain reaction (qRT-PCR) for *mouse double minute 2* (MDM2) and *Cyclin Dependent Kinase Inhibitor 1A* (CDKN1A) compared to *TATA-Box Binding Protein* (TBP). Data adapted from Brackmann et al. 2020, coloured by group (each n = 2, three replicates per individual) and stratified by radiation dose. Significance stars imply the *p*-value thresholds of two-sided t-tests (* *p*-value < 0.05, ** *p*-value < 0.01, *** *p*-value < 0.001). N0 = fibroblasts of cancer-free controls, N1 = fibroblasts of childhood-cancer survivors, N2+  = fibroblasts of childhood-cancer survivors with at least one second primary neoplasm.

**Additional file 4.** IPA settings.

**Additional file 5.** All results of IPA and GO analyses. AF5a. Complete list of predicted upstream regulators. Model 1 considers age at sampling and sex; model 2 considers age at sampling, sex, age at and year of first diagnosis, and tumor type (not used with data from cancer-free controls). Abbreviations: Gy = Gray, N0 = fibroblasts of cancer-free controls, N1 = fibroblasts of childhood-cancer survivors, N2+  = fibroblasts of childhood-cancer survivors with at least one second primary neoplasm, FDR = *p*-value adjusted for false discovery rate. AF5b. Complete list of all affected pathways. Model 1 considers age at sampling and sex; model 2 considers age at sampling, sex, age at and year of first diagnosis, and tumor type (not used with data from cancer-free controls). Abbreviations: Gy = Gray, N0 = fibroblasts of cancer-free controls, N1 = fibroblasts of childhood-cancer survivors, N2+  = fibroblasts of childhood-cancer survivors with at least one second primary neoplasm, FDR = *p*-value adjusted for false discovery rate. AF5c. Complete list of all predicted downstream diseases and biofunctions. Model 1 considers age at sampling and sex; model 2 considers age at sampling, sex, age at and year of first diagnosis, and tumor type (not used with data from cancer-free controls). Abbreviations: Gy = Gray, N0 = fibroblasts of cancer-free controls, N1 = fibroblasts of childhood-cancer survivors, N2+  = fibroblasts of childhood-cancer survivors with at least one second primary neoplasm, FDR = *p*-value adjusted for false discovery rate. rate. AF5d. Complete list of all molecular networks. Model 1 considers age at sampling and sex; model 2 considers age at sampling, sex, age at and year of first diagnosis, and tumor type (not used with data from cancer-free controls). Abbreviations: Gy = Gray, N0 = fibroblasts of cancer-free controls, N1 = fibroblasts of childhood-cancer survivors, N2+  = fibroblasts of childhood-cancer survivors with at least one second primary neoplasm. AF5e. Complete list of Gene Ontology (GO) terms for the top 50 genes (with regard to the *p*-value) from the gene expression models comparing the reaction to treatment based on phenotype, considering age at sampling and sex (model 1). Abbreviations: Gy = Gray, N0 = fibroblasts of cancer-free controls, N1 = fibroblasts of childhood-cancer survivors, N2+  = fibroblasts of childhood-cancer survivors with at least one second primary neoplasm, FDR = *p*-value adjusted for false discovery rate. AF5f. List of genes used as background for the Gene Ontology over-representation analysis.

**Additional file 6.** All enriched pathways after 2 Gray. Heat map showing all pathways that were significantly enriched in one of the three donor groups (false discovery rate adjusted *p*-value < 0.05) in the differential gene expression data after exposure to 2 Gray. Model 1 considers age at sampling and sex, model 2 additionally considers age at and year of diagnosis of the first neoplasm, and tumor type. N0 = fibroblasts of cancer-free controls, N1 = fibroblasts of childhood-cancer survivors, N2+  = fibroblasts of childhood-cancer survivors with at least one second primary neoplasm, * *p*-value < 0.05, ** *p*-value < 0.01, *** *p*-value < 0.001.

**Additional file 7.** Heat maps for differentially expressed genes in affected/modulated pathways.

**Additional file 8. **Molecule activity prediction using Ingenuity Pathway Analysis.

**Additional file 9. **Heat map of genes in relevant molecular networks. Heat map showing all genes and their respective log_2_ fold-change that were associated with molecular networks with a network score > 10 in any of the three donor groups in the differential gene expression data after exposure to 0.05 or 2 Gray. Model 1 considers age at sampling and sex; model 2 considers age at sampling, sex, age at and year of first diagnosis, and tumor type (not used with data from cancer-free controls). Tiles with black frames show genes that were part of the top networks for that dose/group/model combination. N0 = fibroblasts of cancer-free controls, N1 = fibroblasts of childhood-cancer survivors, N2+  = fibroblasts of childhood-cancer survivors with at least one second primary neoplasm.

**Additional file 10.** Results of GO over-representation analyses in gene expression models with interaction terms for donor group. AF10a. Heat maps displaying *p*-value and log_2_ fold-change of the top 5 genes per radiation dose, the direction of log_2_ fold-change per group-wise comparison for expression models including group-dose interaction (with regard to *p*-value). Log_2_ fold-change values can be found inside the tiles. Only the results for model 1 (considering age at sampling and sex) are displayed. N0 = fibroblasts of cancer-free controls, N1 = fibroblasts of donors with a first primary neoplasm, N2+  = fibroblasts of donors with at least one second primary neoplasm. * *p*-valu*e* < 0.05, ** *p*-value < 0.01, *** *p*-value < 0.001 (adjusted for false discovery rate). AF10b. Tree maps displaying clustered top gene ontology terms for the top 50 genes (with regard to *p*-value) for expression models including group-dose interaction (with regard to *p*-value) for each group-wise comparison. Only the results for model 1 (considering age and sex) are displayed. N0 = fibroblasts of cancer-free controls, N1 = fibroblasts of donors with a first primary neoplasm, N2+  = fibroblasts of donors with at least one second primary neoplasm.

**Additional file 11.** Results of differential gene expression and pathway analyses stratified by sex. AF11a. Bar charts showing the number and proportion of up- and downregulated genes stratified by dose, donor group, and sex. Differentially expressed genes were computed comparing irradiated to sham-irradiated fibroblasts after exposure to 0.05 and 2 Gray (false discovery rate adjusted *p*-value < 0.05), considering age at sampling. N0 = fibroblasts of cancer-free controls, N1 = fibroblasts of childhood-cancer survivors, N2+  = fibroblasts of childhood-cancer survivors with at least one second primary neoplasm; * *p*-value < 0.05, ** *p*-value < 0.01, *** *p*-value < 0.001. AF11b. *Upper Half:* Volcano plots showing differential expression after 0.05 Gray stratified by donor group and sex, considering age at sampling. *Lower Half:* Heat map showing top five ranking genes with regard to *p*-value per group considering age at sampling. N0 = fibroblasts of cancer-free controls, N1 = fibroblasts of childhood-cancer survivors, N2+  = fibroblasts of childhood-cancer survivors with at least one second primary neoplasm; * *p*-value < 0.05, ** *p*-value < 0.01, *** *p*-value < 0.001. N.S. = not significant (*p*-value > 0.05). AF11c. *Upper Half:* Volcano plots showing differential expression after 2 Gray stratified by donor group and sex, considering age at sampling. *Lower Half:* Heat map showing top five ranking genes with regard to *p*-value per group considering age at sampling. N0 = fibroblasts of cancer-free controls, N1 = fibroblasts of childhood-cancer survivors, N2+  = fibroblasts of childhood-cancer survivors with at least one second primary neoplasm; * *p*-value < 0.05, ** *p*-value < 0.01, *** *p*-value < 0.001. N.S. = not significant (*p*-value > 0.05). AF11d. Overview of affected (false discovery rate adjusted *p*-value < 0.05) and (in-) activated pathways (|z|≥ 2), predicted upstream effectors, downstream biofunctions and diseases, and observed molecular networks after irradiation with a low (0.05 Gray) or a high dose (2 Gray) ordered by *p*-value, stratified by sex and considering age at sampling. For molecular networks, the network score instead of a *p*-value and no z-score was calculated by *Ingenuity Pathway Analysis*. N0 = fibroblasts of cancer-free controls, N1 = fibroblasts of childhood-cancer survivors, N2+  = fibroblasts of childhood-cancer survivors with at least one second primary neoplasm; *p-value < 0.05, ** *p*-value < 0.01, ***p-value < 0.001. AF11e. Heat map showing all pathways from *Ingenuity Pathway Analysis* that were significantly enriched in one of the three donor groups (false discovery rate adjusted *p*-value < 0.05) using results from the differential gene expression data stratified by sex and considering age at sampling after exposure to 2 Gray. N0 = fibroblasts of cancer-free controls, N1 = fibroblasts of childhood-cancer survivors, N2+  = fibroblasts of childhood-cancer survivors with at least one second primary neoplasm; * *p*-value < 0.05, ** *p*-value < 0.01, *** *p*-value < 0.001.

**Additional file 12.** Results of differential gene expression and pathway analyses after exclusion of self-reported non-Caucasian donors. AF12a. Bar charts showing the proportion of up- and downregulated genes stratified by dose, and donor group after exclusion of self-reported non-Caucasian participants. Differentially expressed genes in irradiated compared to sham-irradiated fibroblasts from N0 = fibroblasts of cancer-free controls, N1 = fibroblasts of childhood-cancer survivors, N2+  = fibroblasts of childhood-cancer survivors with at least one second primary neoplasm. Model 1 considers age at sampling and sex; model 2 considers age at sampling, sex, as well as age at and year of diagnosis of the first neoplasm, and tumor type. AF12b. Overview of affected (false discovery rate adjusted *p*-value < 0.05) and (in-) activated pathways (|z|≥ 2), predicted upstream effectors, downstream biofunctions and diseases, and observed molecular networks after irradiation with a low (0.05 Gray) or a high dose (2 Gray) ordered by *p*-value, after exclusion of self-reported non-Caucasian participants. For molecular networks, the network score instead of a *p*-value and no z-score was calculated by *Ingenuity Pathway Analysis*. Model 1 considers age at sampling and sex. N0 = fibroblasts of cancer-free controls, N1 = fibroblasts of childhood-cancer survivors, N2+  = fibroblasts of childhood-cancer survivors with at least one second primary neoplasm; * *p*-value < 0.05, ** *p-*value < 0.01, *** *p*-value < 0.001. AF12c. Heat map showing all pathways from *Ingenuity Pathway Analysis* that were significantly enriched in one of the three donor groups (false discovery rate adjusted *p*-value < 0.05) in the differential gene expression data after exclusion of self-reported non-Caucasian participants after exposure to 2 Gray. Model 1 considers age at sampling and sex. N0 = fibroblasts of cancer-free controls, N1 = fibroblasts of childhood-cancer survivors, N2+  = fibroblasts of childhood-cancer survivors with at least one second primary neoplasm; * *p*-value < 0.05, ** *p-*value < 0.01, *** *p*-value < 0.001.

**Additional file 13.** Results of pathway analysis after exclusion of genes with less than 20% up- or downregulation.Overview of affected (false discovery rate adjusted at *p*-value < 0.05) and (in-) activated pathways (|z| ≥ 2), predicted upstream effectors, downstream biofunctions and diseases after irradiation with a low (0.05 Gray) or a high dose (2 Gray) ordered by p-value.* *p-*-value < 0.05, ** *p-*-value < 0.01, *** *p-*-value < 0.001. Model 1: considering age at sampling and sex, model 2: considering age at sampling, sex, age at and year of diagnosis of the first neoplasm, and tumor type (not used with data from N0), N0 = fibroblasts of cancer-free controls, N1 = fibroblasts of childhood-cancer survivors, N2+ = fibroblasts of childhood-cancer survivors with at least one second primary neoplasm.

## Data Availability

All data generated or analyzed during this study are included in this published article and its additional information files.

## Abbreviations

ADAMTS17: ADAM metallopeptidase with thrombospondin type 1 motif 17
ADNP-AS1: ADNP antisense ribonucleic acid 1
AKT1: RAC-alpha serine/threonine-protein kinase 1
ALG9: ALG9 alpha-1,2-mannosyltransferase
BBC3: BCL2 binding component 3
BCL2: B-cell lymphoma 2
BIN3: Bridging integrator 3
BRCA2: Breast cancer gene 2, deoxyribonucleic acid repair associated
BRD7: Bromodomain containing 7
BTG2: BTG anti-proliferation factor 2
CAMTA2: Calmodulin binding transcription activator 2
CBX7: Chromobox homolog 7
CCNE2: Cyclin E2
CCNG1: Cyclin G1
CDC6: Cell division cycle 6
CDKN1A: Cyclin Dependent Kinase Inhibitor 1A
CLUHP3: Clustered mitochondria homolog pseudogene 3
CHEK2: Checkpoint kinase 2
CHUK: Inhibitor of nuclear factor kappa-B kinase subunit alpha
COBLL1: Cordon-bleu WH2 repeat protein-like 1
DAB2: Disabled homolog 2
DDB2: Deoxyribonucleic acid damage-binding protein 2
DEG: Differentially expressed genes
DLEU2: Deleted in lymphocytic leukemia 2
DNA: Deoxyribonucleic acid
DUOXA1: Dual oxidase maturation factor 1
E2F1: E2F transcription factor 1
EEF1AKNMT: Eukaryotic translation elongation factor 1 alpha lysine and N-terminal methyltransferase
FDR: False discovery rate
FDXR: Ferredoxin reductase
GADD45A: Growth arrest and deoxyribonucleic acid-damage-inducible protein GADD45 alpha
GO: Gene ontology
Gy: Gray
HAS3: Hyaluronan synthase 3
HDIR: High dose of ionizing radiation
HLX: H2.0 like homeobox
HMGCR: 3-Hydroxy-3-methyl-glutaryl-coenzyme A reductase
HSPA8: Heat shock protein family A (Hsp70) member 8
Hsp90α: Heat shock protein 90-α
IL15: Interleukin 15
IPA: Ingenuity pathway analysis
IQR: Interquartile range
IR: Ionizing radiation
IRF1: Interferon regulatory factor 1
JUN: Jun proto-oncogene, AP-1 transcription factor subunit
LDIR: Low dose of ionizing radiation
LFC: Log_2_ fold-change
LIN9: Lin-9 homolog
LINC00601: Long intergenic non-protein coding ribonucleic acid 601
LINC02257: Long intergenic non-protein coding ribonucleic acid 2257
MDM2: Mouse double minute 2
mRNA: Messenger ribonucleic acid
MSH6: MutS homolog 6
MYBL2: MYB proto-oncogene like 2
MYO7B: Myosin VIIB
N0: Fibroblasts of cancer-free controls
N1: Fibroblasts of donors with a first primary neoplasm in childhood
N2+: Fibroblasts of donors with a first primary neoplasm in childhood and at least one second primary neoplasm
NaN: Not a number
ncRNA: Non-coding ribonucleic acid
NF-kB: Nuclear factor ‘kappa-light-chain-enhancer’ of activated B-cells
NUP153: Nucleoporin 153
p53: Tumor protein 53
PATZ1: POZ/BTB and AT hook containing zinc finger 1
PCNA: Proliferating cell nuclear antigen
PGR: Progesterone receptor
PIDD1: P53-induced death domain protein 1
POLH: Deoxyribonucleic acid polymerase eta
PPM1D: Protein phosphatase, Mg2 + /MN2+ dependent 1D
PPRC1: Peroxisome proliferator-activated receptor gamma coactivator-related protein 1
qPCR: Quantitative real-time polymerase chain reaction
RAB41: Member RAS oncogene family
RAD9A: Cell cycle checkpoint control protein RAD9A
RIPK1: Receptor interacting serine/threonine kinase 1
RNA: Ribonucleic acid
RNA-Seq: Messenger ribonucleic acid-sequencing
SESN1: Sestrin 1
SESN2: Sestrin 2
SLK: STE20-like serine/threonine-protein kinase
SOD1: Superoxide dismutase 1
SPDYE3: Speedy/RINGO cell cycle regulator family member E3
SRF: Serum response factor
TBC1D10A: TBC1 domain family member 10A
TFCP2: Transcription factor CP2
TIAF1: Transforming growth factor beta-induced anti-apoptotic factor 1
TNFRSF10A: Tumor necrosis factor receptor superfamily member 10a
TP53INP1: Tumor protein P53 inducible nuclear protein 1
ZNF2: Zinc finger protein 2
ZNF226: Zinc finger protein 226
