# Supplementary material for: Radiation-response in primary fibroblasts of long-term survivors of childhood cancer with and without second primary neoplasms: the KiKme study
Source: Mol Med. 2022 Sep 6;28:105. doi: 10.1186/s10020-022-00520-6 (PMC9450413; doi:10.1186/s10020-022-00520-6)
Supplement: Supplementary file 2 — Additional file 2. Comparisons of FDR and LFC across donor groups. Comparison of differential expression (DE) and its direction between donor groups. [file 10020_2022_520_MOESM2_ESM.pdf]

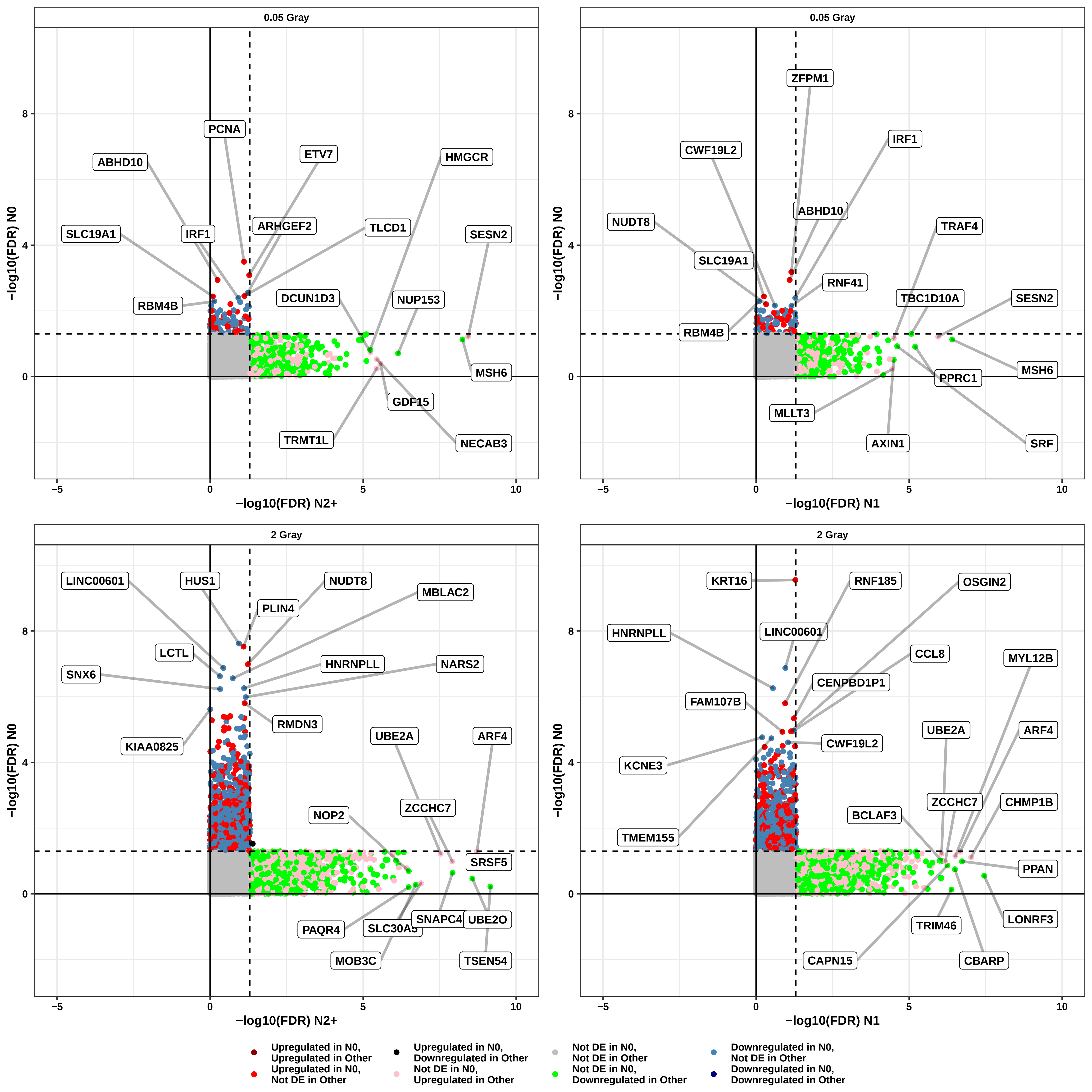

0.05 Gray
ABHD10
PCNA
ETV7
HMGCR
SLC19A1
IRF1
ARHGEF2
TLCD1
SESN2
RBM4B
DCUN1D3
NUP153
MSH6
GDF15
TRMT1L
NECAB3
−log10(FDR) N0
−log10(FDR) N2+
0.05 Gray
ZFPM1
IRF1
CWF19L2
NUDT8
ABHD10
TRAF4
SLC19A1
RNF41
TBC1D10A
SESN2
RBM4B
MSH6
PPRC1
MLLT3
AXIN1
SRF
−log10(FDR) N0
−log10(FDR) N1
2 Gray
LINC00601
HUS1
NUDT8
MBLAC2
PLIN4
LCTL
HNRNPLL
NARS2
SNX6
RMDN3
KIAA0825
UBE2A
ARF4
NOP2
ZCCHC7
SRSF5
PAQR4
SLC30A5
SNAPC4
UBE2O
MOB3C
TSEN54
−log10(FDR) N0
−log10(FDR) N2+
2 Gray
KRT16
RNF185
OSGIN2
HNRNPLL
LINC00601
CCL8
MYL12B
CENPBD1P1
FAM107B
UBE2A
ARF4
CWF19L2
KCNE3
ZCCHC7
CHMP1B
BCLAF3
TMEM155
PPAN
TRIM46
LONRF3
CAPN15
CBARP
−log10(FDR) N0
−log10(FDR) N1
Upregulated in N0, Upregulated in Other
Upregulated in N0, Not DE in Other
Upregulated in N0, Downregulated in Other
Not DE in N0, Upregulated in Other
Not DE in N0, Not DE in Other
Not DE in N0, Downregulated in Other
Downregulated in N0, Not DE in Other
Downregulated in N0, Downregulated in Other

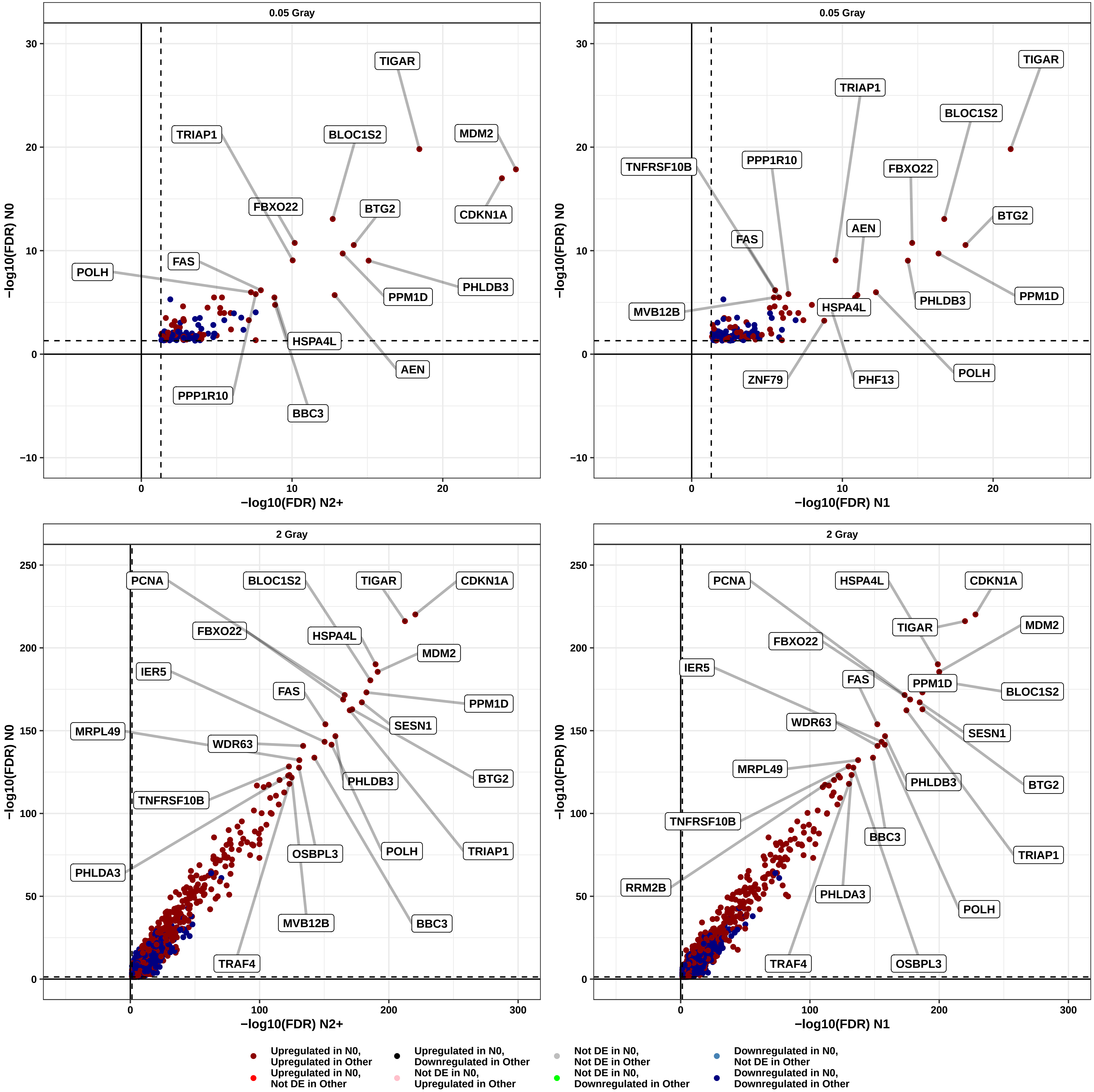

0.05 Gray
−log10(FDR) N2+
−log10(FDR) N0
TIGAR
MDM2
TRIAP1
BLOC1S2
FBXO22
BTG2
CDKN1A
FAS
POLH
PPM1D
PHLDB3
HSPA4L
AEN
PPP1R10
BBC3
−log10(FDR) N1
TNFRSF10B
PPP1R10
MVB12B
ZNF79
PHF13
2 Gray
PCNA
SESN1
IER5
MRPL49
WDR63
OSBPL3
MVB12B
TRAF4
PHLDA3
RRM2B
Upregulated in N0, Upregulated in Other
Upregulated in N0, Not DE in Other
Upregulated in N0, Downregulated in Other
Not DE in N0, Upregulated in Other
Not DE in N0, Not DE in Other
Not DE in N0, Downregulated in Other
Downregulated in N0, Not DE in Other
Downregulated in N0, Downregulated in Other
