# Supplementary material for: Radiation-response in primary fibroblasts of long-term survivors of childhood cancer with and without second primary neoplasms: the KiKme study
Source: Mol Med. 2022 Sep 6;28:105. doi: 10.1186/s10020-022-00520-6 (PMC9450413; doi:10.1186/s10020-022-00520-6)
Supplement: Supplementary file 3 — Additional file 3. Primer for and results of qRT-PCR. AF3a. Constructed and purchased primer sequences for the qPCR. AF3b. Log10 relative expression measured in quantitative polymerase reverse transcriptase chain reaction (qRT-PCR) for selected genes compared to TATA-Box Binding Protein (TBP), stratified by group (each n = 6, three replicates per individuals) and radiation dose. Significance stars imply the p-value thresholds of two-sided t-tests after adjustment for false discovery rate (* p-value < 0.05, ** p-value < 0.01, *** p-value < 0.001). N0 = fibroblasts of cancer-free controls, N1 = fibroblasts of childhood-cancer survivors, N2+ = fibroblasts of childhood-cancer survivors with at least one second primary neoplasm. AF3c. Log10-relative expression measured in quantitative polymerase reverse transcriptase chain reaction (qRT-PCR) for mouse double minute 2 (MDM2) and Cyclin Dependent Kinase Inhibitor 1A (CDKN1A) compared to TATA-Box Binding Protein (TBP). Data adapted from Brackmann et al. 2020, coloured by group (each n = 2, three replicates per individual) and stratified by radiation dose. Significance stars imply the p-value thresholds of two-sided t-tests (* p-value < 0.05, ** p-value < 0.01, *** p-value < 0.001). N0 = fibroblasts of cancer-free controls, N1 = fibroblasts of childhood-cancer survivors, N2+ = fibroblasts of childhood-cancer survivors with at least one second primary neoplasm. [file 10020_2022_520_MOESM3_ESM.docx]

**Additional File 3a:** Constructed and purchased primer sequences for the qRT-PCR.

| RNA PCNA F | GCTCTTCCCTTACGCAAGTCT |
| --- | --- |
| RNA PCNA R | TAGCTGGTTTCGGCTTCAGG |

| RNA FANCD2F | TGCATGTATGTTTGAAGTATGGGC |
| --- | --- |
| RNA FANCD2R | GGAATGGAAATGGGCATAGAAG |

| RNA IL1B F | TTCGAGGCACAAGGCACAA |
| --- | --- |
| RNA IL1B R | TGGCTGCTTCAGACACTTGAG |

| CCNE2 F (4) | CACCCGGGTTACCAATGACA |
| --- | --- |
| CCNE2 R (4) | GGATGCACTTTTTGCCCTTCTTA |

**Hs_IL6_1_SG QuantiTect Primer Assay CATALOG No. - QT00083720**

**Hs_DDB2_1_SG QuantiTect Primer Assay CATALOG No. - QT00062986**

**Hs_MSH6_1_SG QuantiTect Primer Assay CATALOG No. - QT00054663**

**Hs_EGR1_1_SG QuantiTect Primer Assay CATALOG No. - QT00218505**


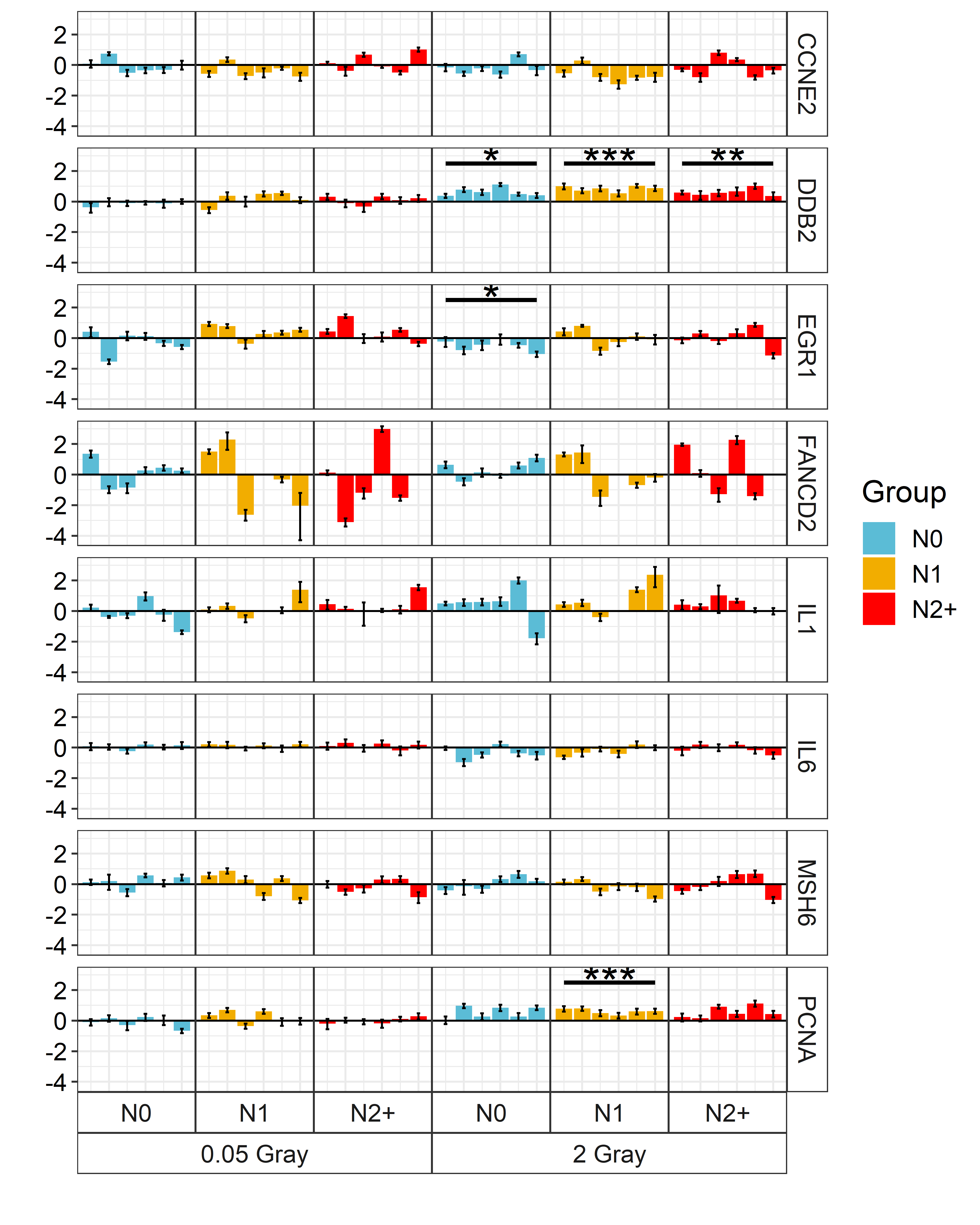


**Additional File 3b:** Log_10_ relative expression measured in quantitative reverse transcriptase polymerase chain reaction (qRT-PCR) for selected genes compared to *TATA-Box Binding Protein* (TBP), stratified by group (each n= 6, three replicates per individuals) and radiation dose. Asterisks imply the *p*-value thresholds of two-sided t-tests after adjustment for false discovery rate (* *p*-value < 0.05, ** *p*-value < 0.01, *** *p*-value < 0.001). N0 = fibroblasts of cancer-free controls, N1 = fibroblasts of childhood-cancer survivors, N2+ = fibroblasts of childhood-cancer survivors with at least one second primary neoplasm.

**
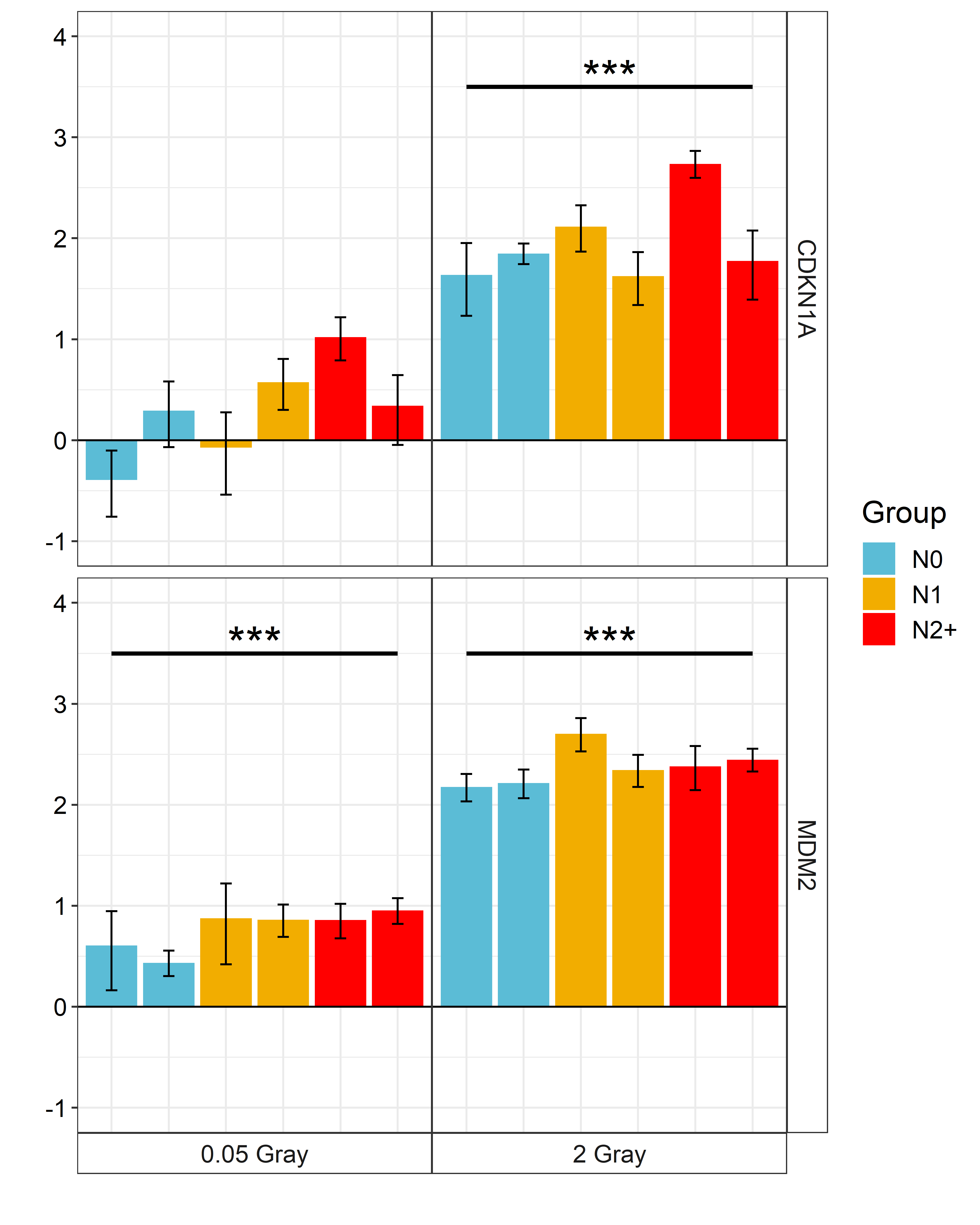
**

**Additional File 3c:** Log_10_-relative expression measured in quantitative reverse transcriptase polymerase chain reaction (qRT-PCR) for *mouse double minute 2* (MDM2) and *Cyclin Dependent Kinase Inhibitor 1A* (CDKN1A) compared to *TATA-Box Binding Protein* (TBP). Data adapted from Brackmann et al. 2020, coloured by group (each n = 2, three replicates per individuals) and stratified by radiation dose. Asterisks imply the *p*-value thresholds of two-sided t-tests (* *p*-value < 0.05, ** *p*-value < 0.01, *** *p*-value < 0.001). N0 = fibroblasts of cancer-free controls, N1 = fibroblasts of childhood-cancer survivors, N2+ = fibroblasts of childhood-cancer survivors with at least one second primary neoplasm.
