# Supplementary material for: Radiation-response in primary fibroblasts of long-term survivors of childhood cancer with and without second primary neoplasms: the KiKme study
Source: Mol Med. 2022 Sep 6;28:105. doi: 10.1186/s10020-022-00520-6 (PMC9450413; doi:10.1186/s10020-022-00520-6)
Supplement: Supplementary file 4 — Additional file 4. IPA settings. [file 10020_2022_520_MOESM4_ESM.docx]

**Additional File 4: *IPA* Settings.**

Consider only molecules and/or relationships where

(species = Human) AND

(confidence = Experimentally Observed) AND

(tissues/cell lines = Cos-7 cells OR Fibroblasts OR MEF cells OR Other Fibroblast cell lines OR Swiss 3T3 cells OR Fibroblast cell lines not otherwise specified OR NIH/3T3 cells OR 3T3-L1 cells) AND

(mol. types = biologic drug OR chemical - endogenous mammalian OR chemical - endogenous non-mammalian OR chemical - kinase inhibitor OR chemical - other OR chemical - protease inhibitor OR chemical drug OR chemical reagent OR chemical toxicant OR complex OR cytokine OR disease OR enzyme OR function OR G-protein coupled receptor OR group OR growth factor OR ion channel OR kinase OR ligand-dependent nuclear receptor OR mature microRNA OR microRNA OR other OR peptidase OR phosphatase OR transcription regulator OR translation regulator OR transmembrane receptor OR transporter) AND

(data sources = An Open Access Database of Genome-wide Association Results OR BIND OR BioGRID OR Catalogue Of Somatic Mutations In Cancer (COSMIC) OR Chemical Carcinogenesis Research Information System (CCRIS) OR ClinicalTrials.gov OR ClinVar OR Cognia OR DIP OR DrugBank OR Gene Ontology (GO) OR GVK Biosciences OR Hazardous Substances Data Bank (HSDB) OR HumanCyc OR Ingenuity Expert Findings OR Ingenuity ExpertAssist Findings OR IntAct OR Interactome studies OR MIPS OR miRBase OR miRecords OR Mouse Genome Database (MGD) OR Obesity Gene Map Database OR Online Mendelian Inheritance in Man (OMIM) OR TarBase OR TargetScan Human)
