# Supplementary material for: Radiation-response in primary fibroblasts of long-term survivors of childhood cancer with and without second primary neoplasms: the KiKme study
Source: Mol Med. 2022 Sep 6;28:105. doi: 10.1186/s10020-022-00520-6 (PMC9450413; doi:10.1186/s10020-022-00520-6)
Supplement: Supplementary file 6 — Additional file 6. All enriched pathways after 2 Gray. Heat map showing all pathways that were significantly enriched in one of the three donor groups (false discovery rate adjusted p-value < 0.05) in the differential gene expression data after exposure to 2 Gray. Model 1 considers age at sampling and sex, model 2 additionally considers age at and year of diagnosis of the first neoplasm, and tumor type. N0 = fibroblasts of cancer-free controls, N1 = fibroblasts of childhood-cancer survivors, N2+ = fibroblasts of childhood-cancer survivors with at least one second primary neoplasm, * p-value < 0.05, ** p-value < 0.01, *** p-value < 0.001. [file 10020_2022_520_MOESM6_ESM.docx]

**
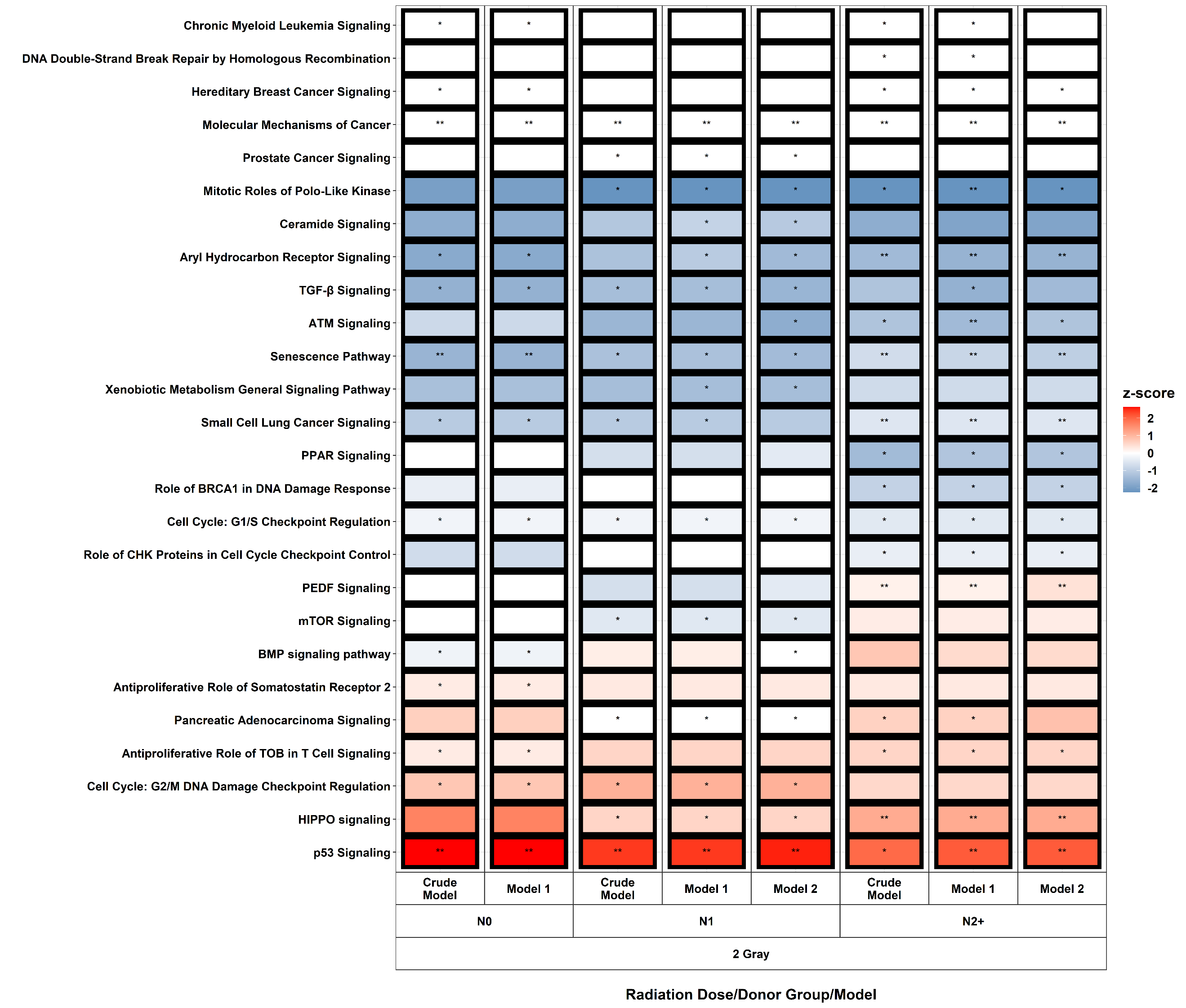
**

**AdditionalFile 6:** Heat map showing all pathways that were significantly enriched in one of the three donor groups (false discovery rate adjusted p-value < 0.05) in the differential gene expression data after exposure to 2 Gray. Model 1 considers age at sampling and sex, model 2 additionally considers age at and year of diagnosis of the first neoplasm, and tumor type. N0 = fibroblasts of cancer-free controls, N1 = fibroblasts of childhood-cancer survivors, N2+ = fibroblasts of childhood-cancer survivors with at least one second primary neoplasm, * p-value < 0.05, ** p-value < 0.01, *** p-value < 0.001.
