# Supplementary material for: Radiation-response in primary fibroblasts of long-term survivors of childhood cancer with and without second primary neoplasms: the KiKme study
Source: Mol Med. 2022 Sep 6;28:105. doi: 10.1186/s10020-022-00520-6 (PMC9450413; doi:10.1186/s10020-022-00520-6)
Supplement: Supplementary file 7 — Additional file 7. Heat maps for differentially expressed genes in affected/modulated pathways. [file 10020_2022_520_MOESM7_ESM.pptx]

## Slide 1
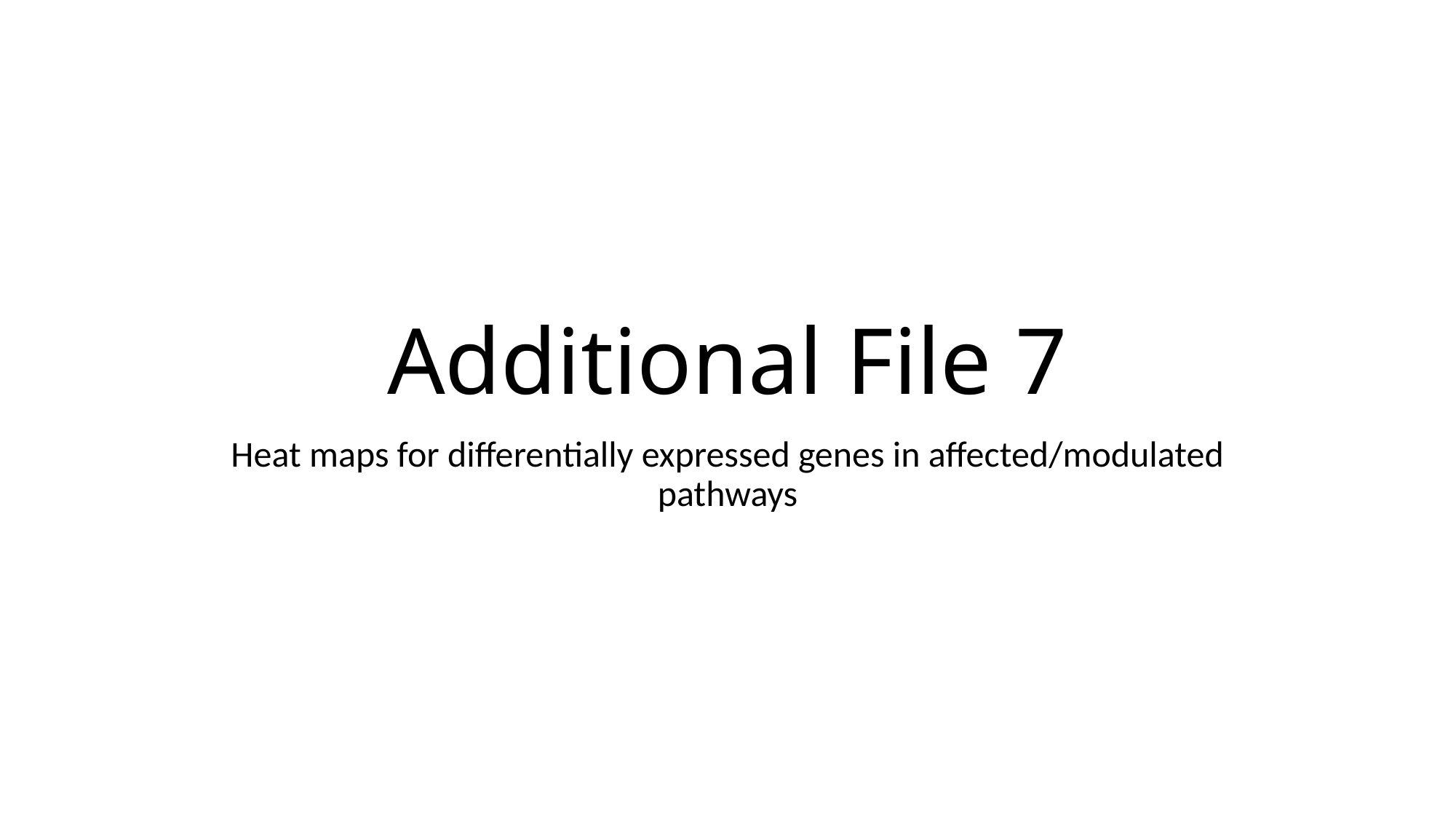

# Additional File 7
Heat maps for differentially expressed genes in affected/modulated pathways

## Slide 2
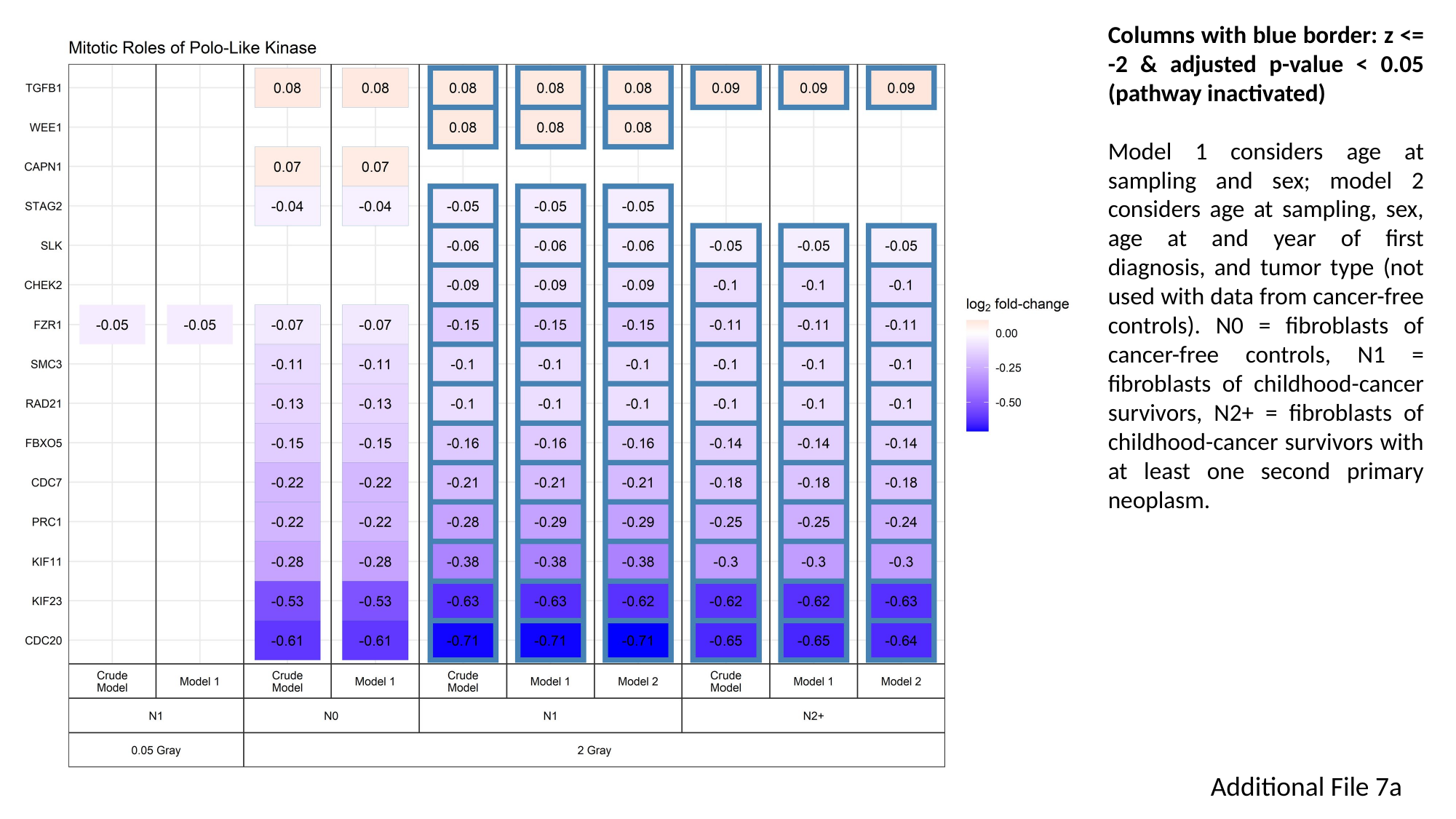

Columns with blue border: z <= -2 & adjusted p-value < 0.05 (pathway inactivated)
Model 1 considers age at sampling and sex; model 2 considers age at sampling, sex, age at and year of first diagnosis, and tumor type (not used with data from cancer-free controls). N0 = fibroblasts of cancer-free controls, N1 = fibroblasts of childhood-cancer survivors, N2+ = fibroblasts of childhood-cancer survivors with at least one second primary neoplasm.
Additional File 7a

## Slide 3
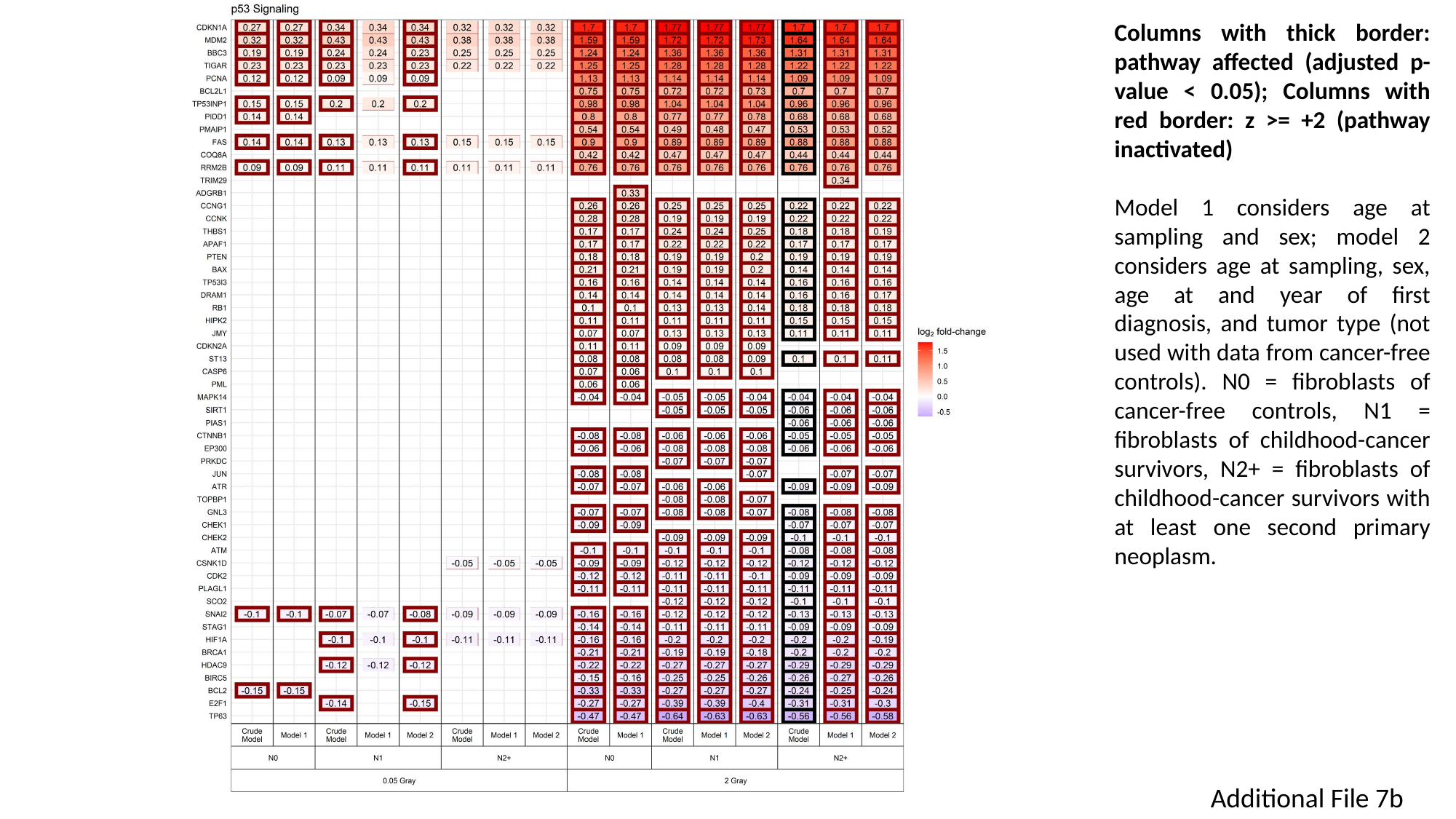

Columns with thick border: pathway affected (adjusted p-value < 0.05); Columns with red border: z >= +2 (pathway inactivated)
Model 1 considers age at sampling and sex; model 2 considers age at sampling, sex, age at and year of first diagnosis, and tumor type (not used with data from cancer-free controls). N0 = fibroblasts of cancer-free controls, N1 = fibroblasts of childhood-cancer survivors, N2+ = fibroblasts of childhood-cancer survivors with at least one second primary neoplasm.
Additional File 7b

## Slide 4
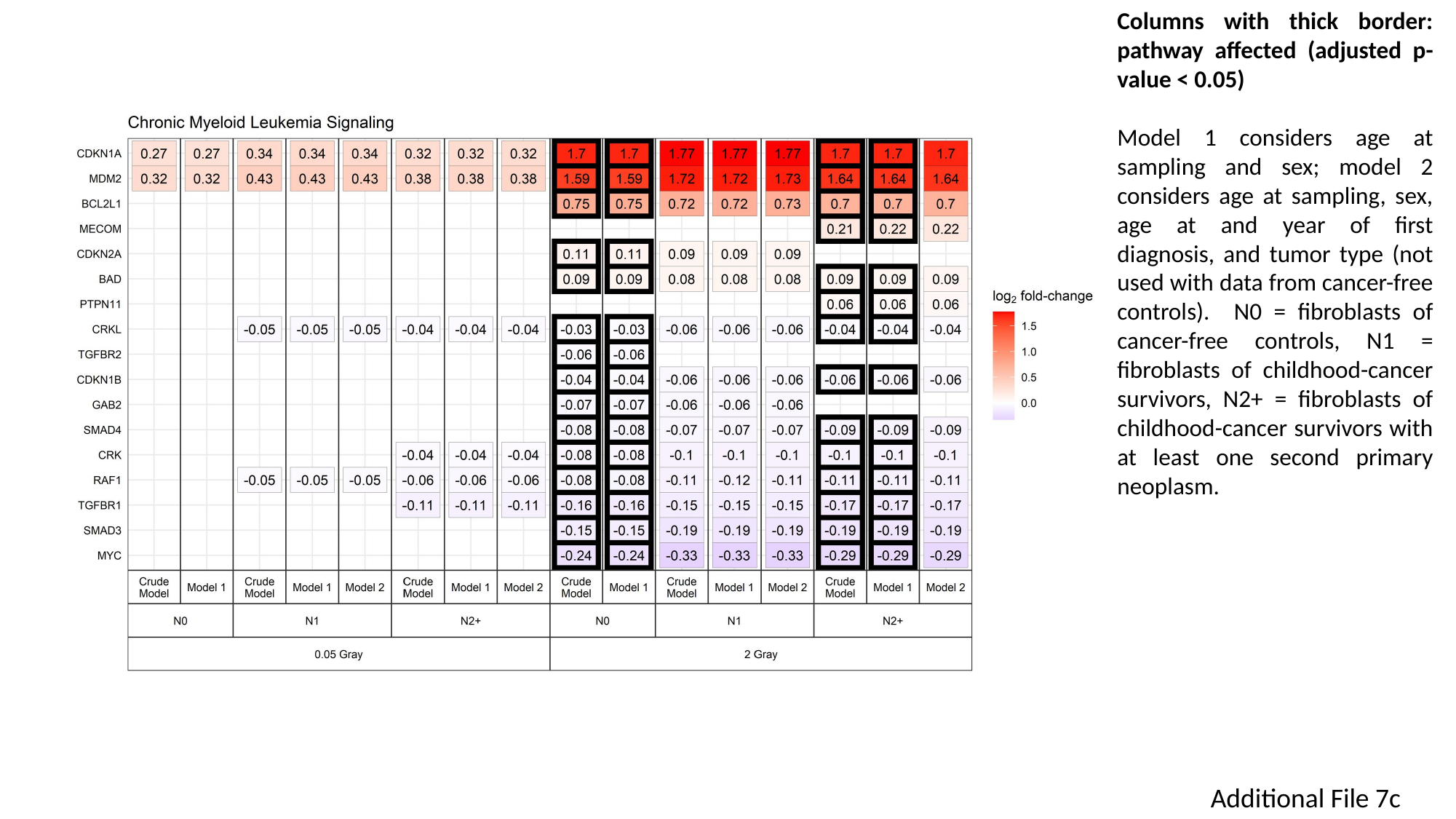

Columns with thick border: pathway affected (adjusted p-value < 0.05)
Model 1 considers age at sampling and sex; model 2 considers age at sampling, sex, age at and year of first diagnosis, and tumor type (not used with data from cancer-free controls). N0 = fibroblasts of cancer-free controls, N1 = fibroblasts of childhood-cancer survivors, N2+ = fibroblasts of childhood-cancer survivors with at least one second primary neoplasm.
Additional File 7c

## Slide 5
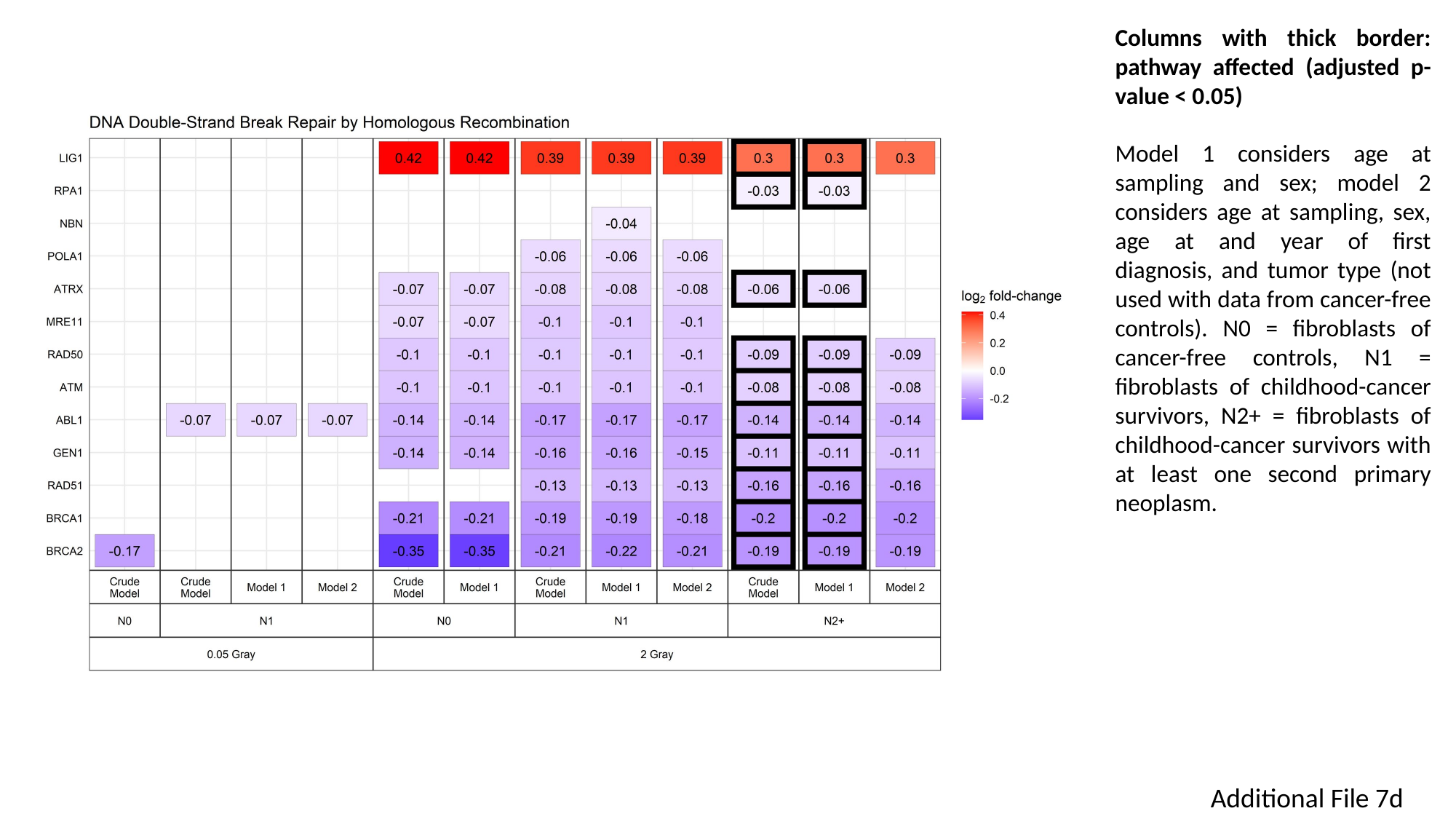

Columns with thick border: pathway affected (adjusted p-value < 0.05)
Model 1 considers age at sampling and sex; model 2 considers age at sampling, sex, age at and year of first diagnosis, and tumor type (not used with data from cancer-free controls). N0 = fibroblasts of cancer-free controls, N1 = fibroblasts of childhood-cancer survivors, N2+ = fibroblasts of childhood-cancer survivors with at least one second primary neoplasm.
Additional File 7d

## Slide 6
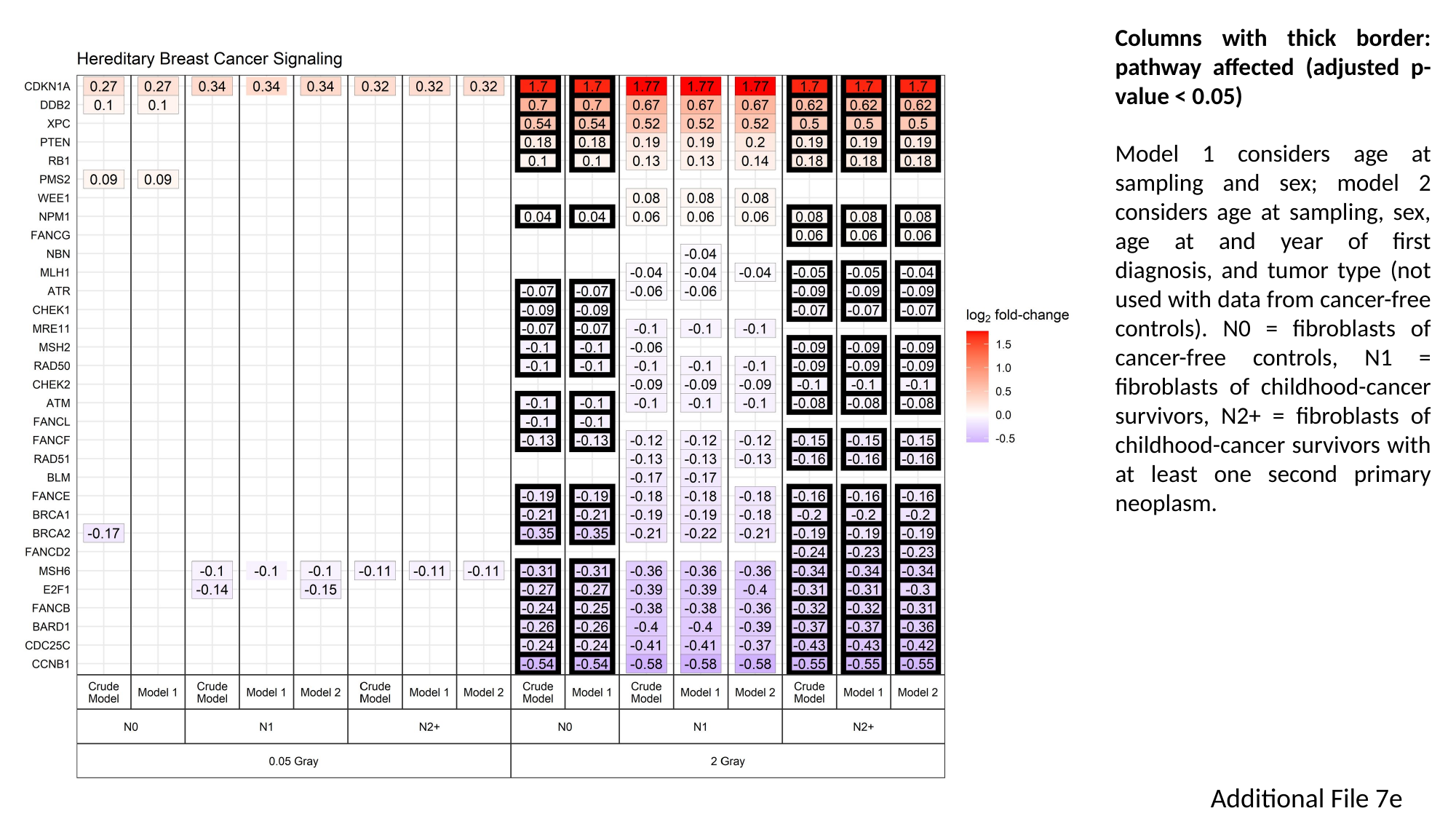

Columns with thick border: pathway affected (adjusted p-value < 0.05)
Model 1 considers age at sampling and sex; model 2 considers age at sampling, sex, age at and year of first diagnosis, and tumor type (not used with data from cancer-free controls). N0 = fibroblasts of cancer-free controls, N1 = fibroblasts of childhood-cancer survivors, N2+ = fibroblasts of childhood-cancer survivors with at least one second primary neoplasm.
Additional File 7e

## Slide 7
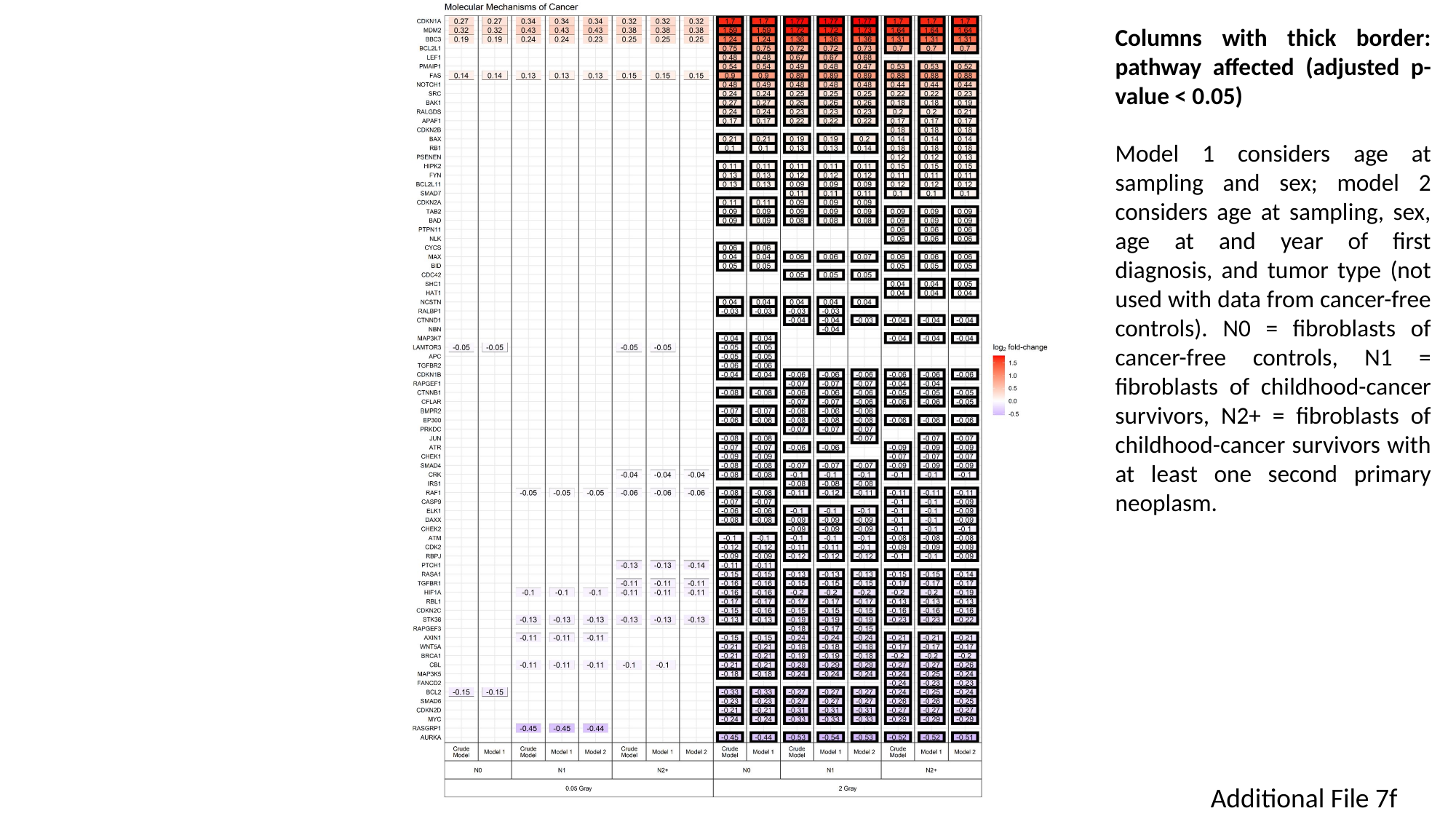

Columns with thick border: pathway affected (adjusted p-value < 0.05)
Model 1 considers age at sampling and sex; model 2 considers age at sampling, sex, age at and year of first diagnosis, and tumor type (not used with data from cancer-free controls). N0 = fibroblasts of cancer-free controls, N1 = fibroblasts of childhood-cancer survivors, N2+ = fibroblasts of childhood-cancer survivors with at least one second primary neoplasm.
Additional File 7f

## Slide 8
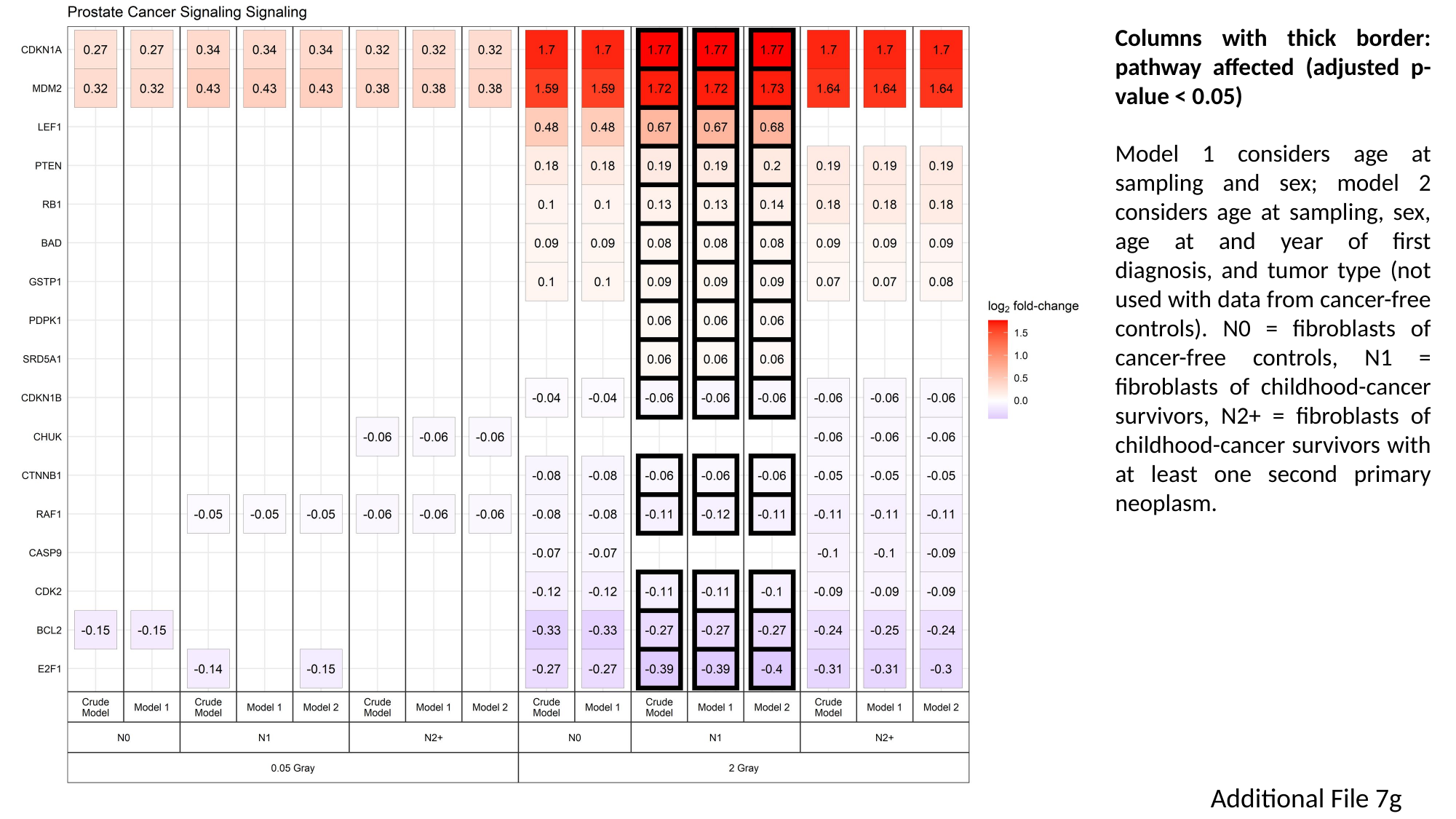

Columns with thick border: pathway affected (adjusted p-value < 0.05)
Model 1 considers age at sampling and sex; model 2 considers age at sampling, sex, age at and year of first diagnosis, and tumor type (not used with data from cancer-free controls). N0 = fibroblasts of cancer-free controls, N1 = fibroblasts of childhood-cancer survivors, N2+ = fibroblasts of childhood-cancer survivors with at least one second primary neoplasm.
Additional File 7g
