# Supplementary material for: Radiation-response in primary fibroblasts of long-term survivors of childhood cancer with and without second primary neoplasms: the KiKme study
Source: Mol Med. 2022 Sep 6;28:105. doi: 10.1186/s10020-022-00520-6 (PMC9450413; doi:10.1186/s10020-022-00520-6)
Supplement: Supplementary file 8 — Additional file 8. Molecule activity prediction using Ingenuity Pathway Analysis. [file 10020_2022_520_MOESM8_ESM.pptx]

## Slide 1
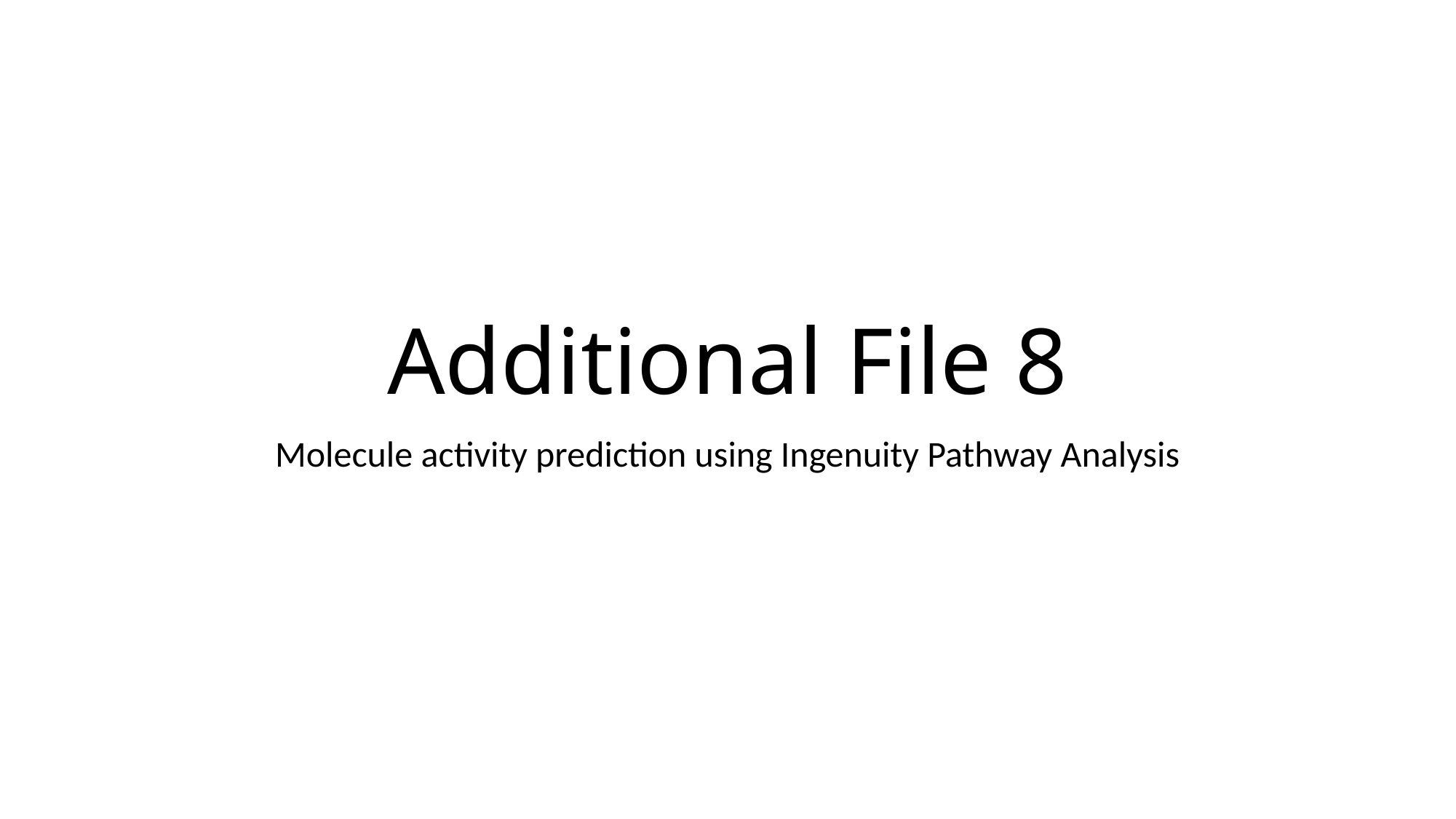

# Additional File 8
Molecule activity prediction using Ingenuity Pathway Analysis

## Slide 2
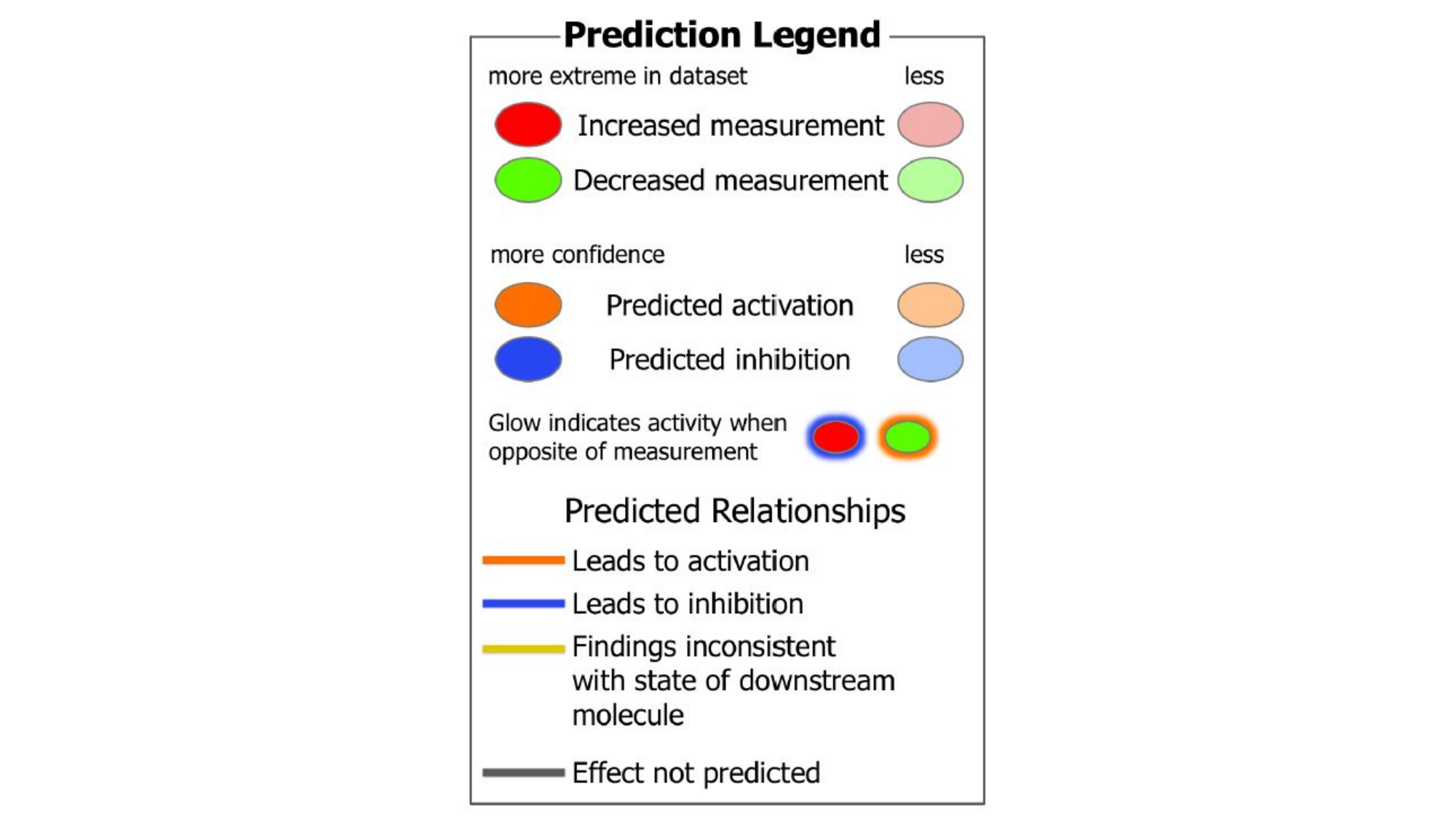

## Slide 3
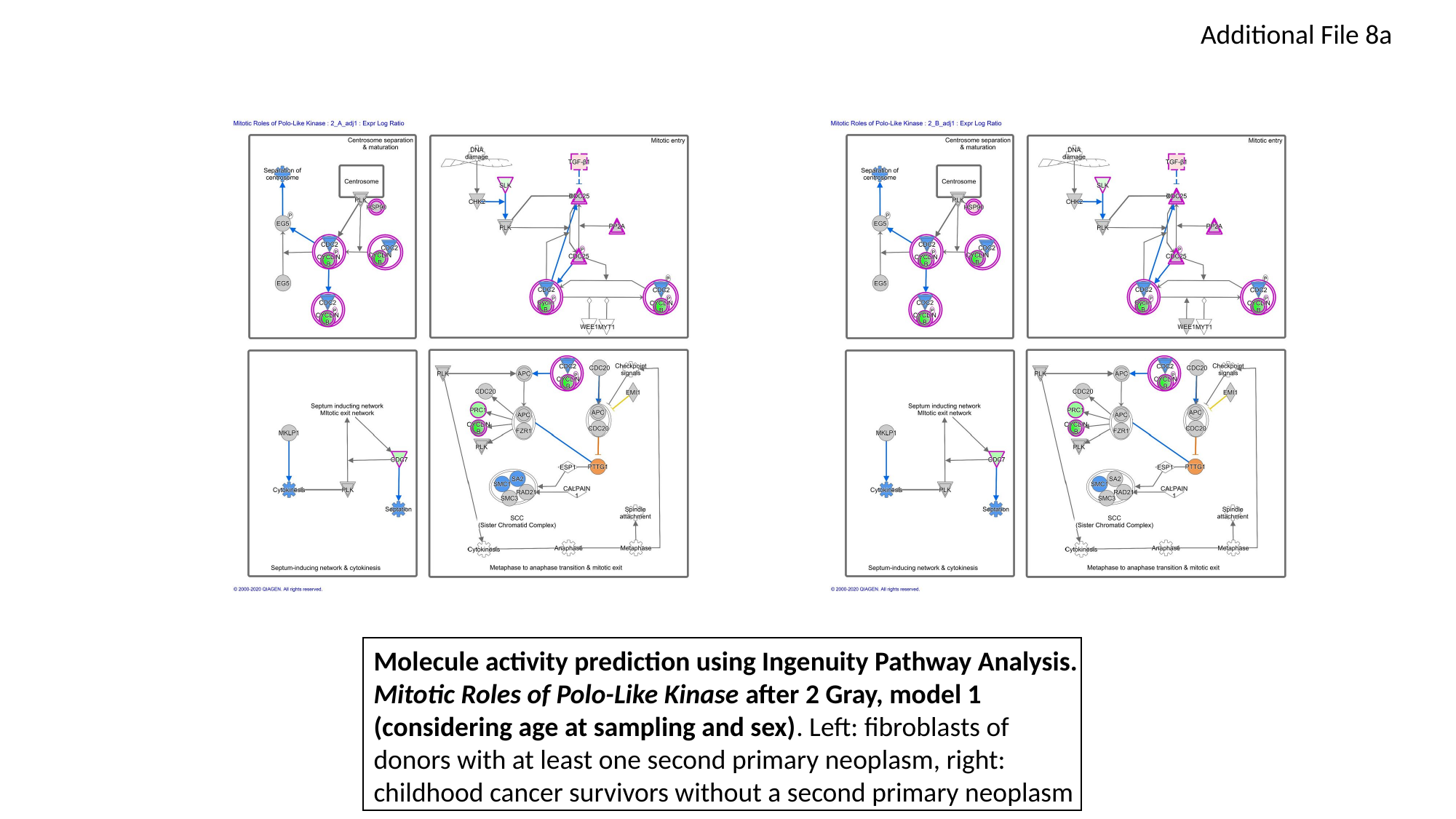

Additional File 8a
Molecule activity prediction using Ingenuity Pathway Analysis. Mitotic Roles of Polo-Like Kinase after 2 Gray, model 1 (considering age at sampling and sex). Left: fibroblasts of donors with at least one second primary neoplasm, right: childhood cancer survivors without a second primary neoplasm

## Slide 4
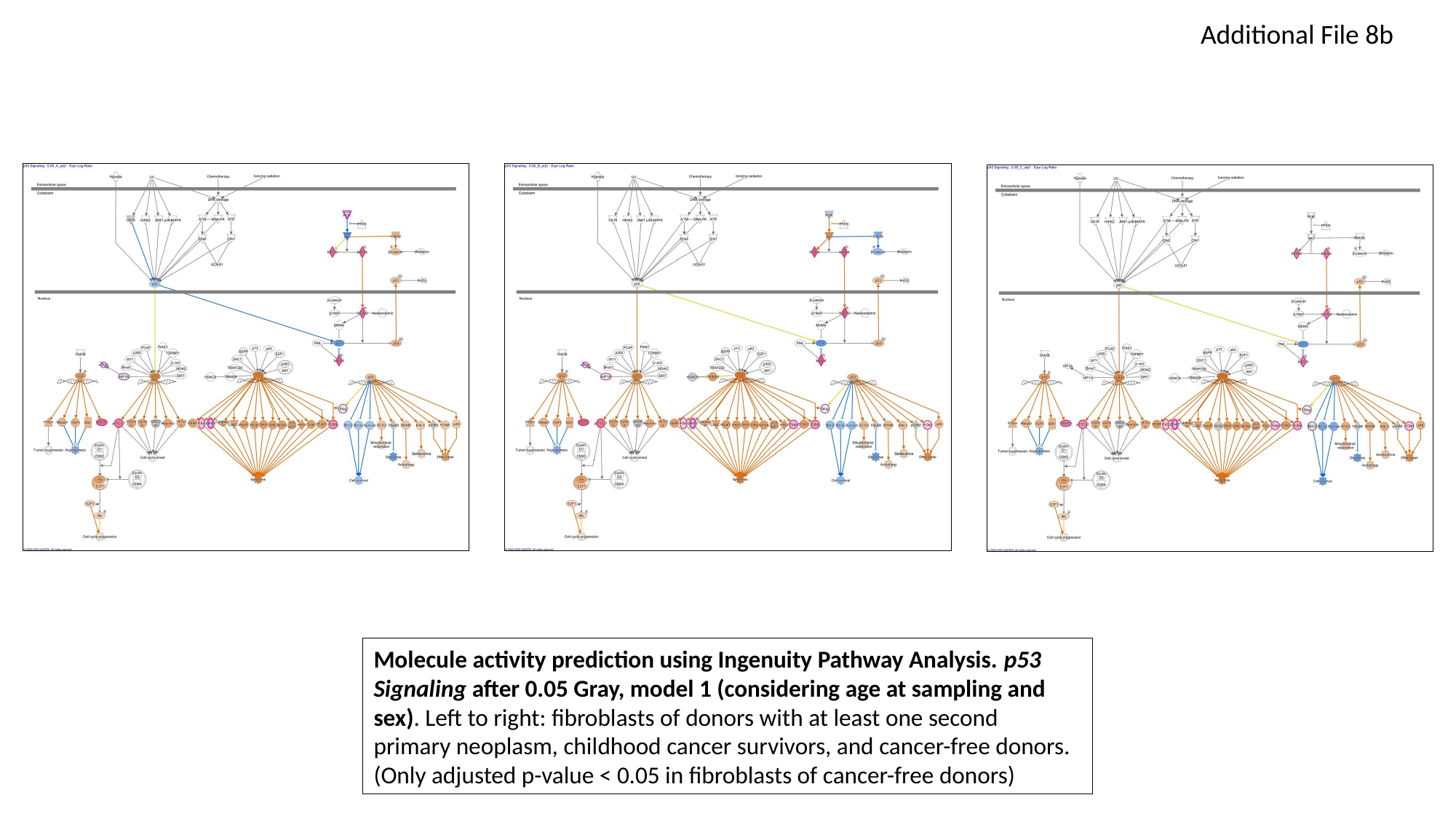

Additional File 8b
Molecule activity prediction using Ingenuity Pathway Analysis. p53 Signaling after 0.05 Gray, model 1 (considering age at sampling and sex). Left to right: fibroblasts of donors with at least one second primary neoplasm, childhood cancer survivors, and cancer-free donors. (Only adjusted p-value < 0.05 in fibroblasts of cancer-free donors)

## Slide 5
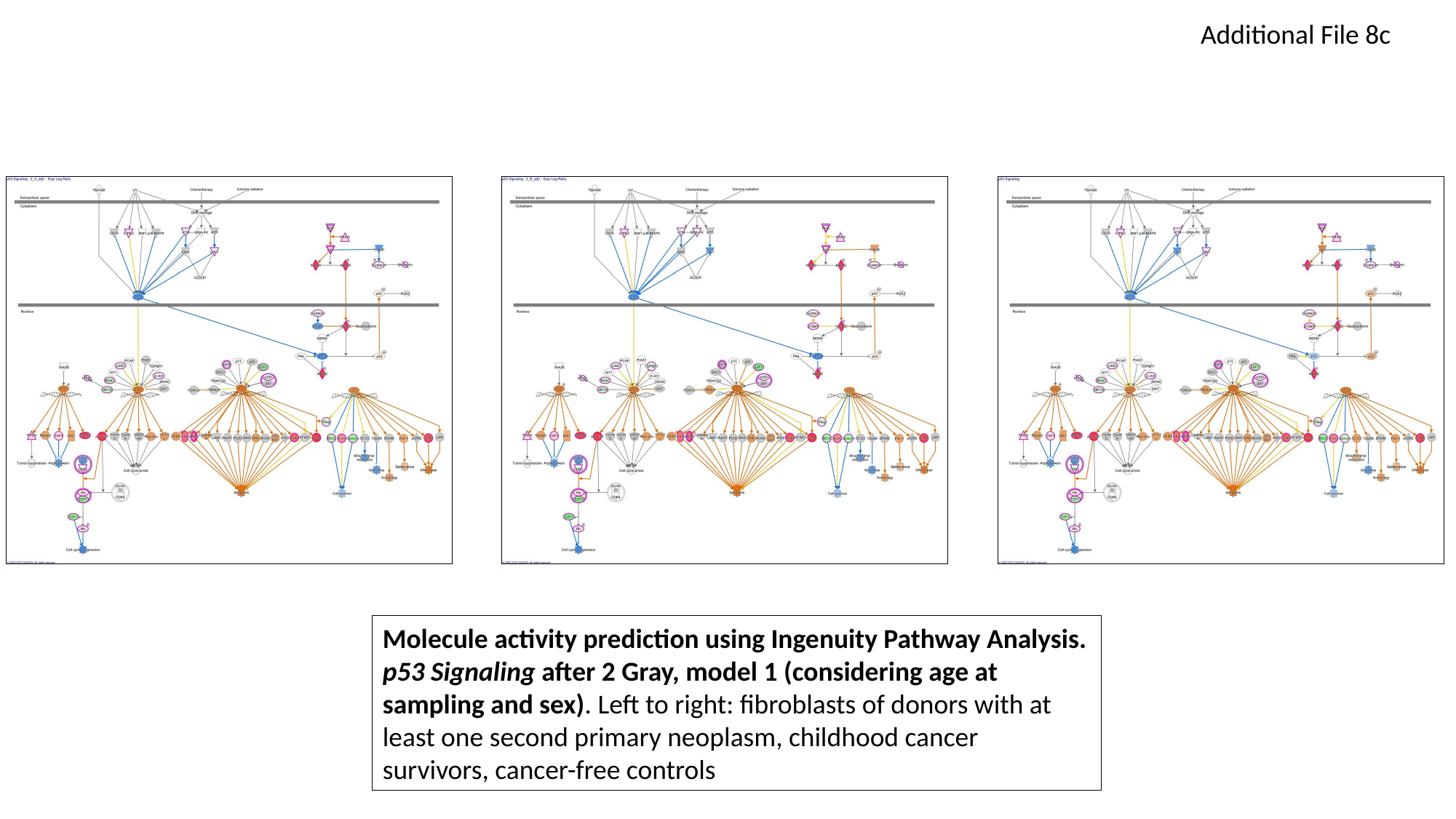

Additional File 8c
Molecule activity prediction using Ingenuity Pathway Analysis. p53 Signaling after 2 Gray, model 1 (considering age at sampling and sex). Left to right: fibroblasts of donors with at least one second primary neoplasm, childhood cancer survivors, cancer-free controls

## Slide 6
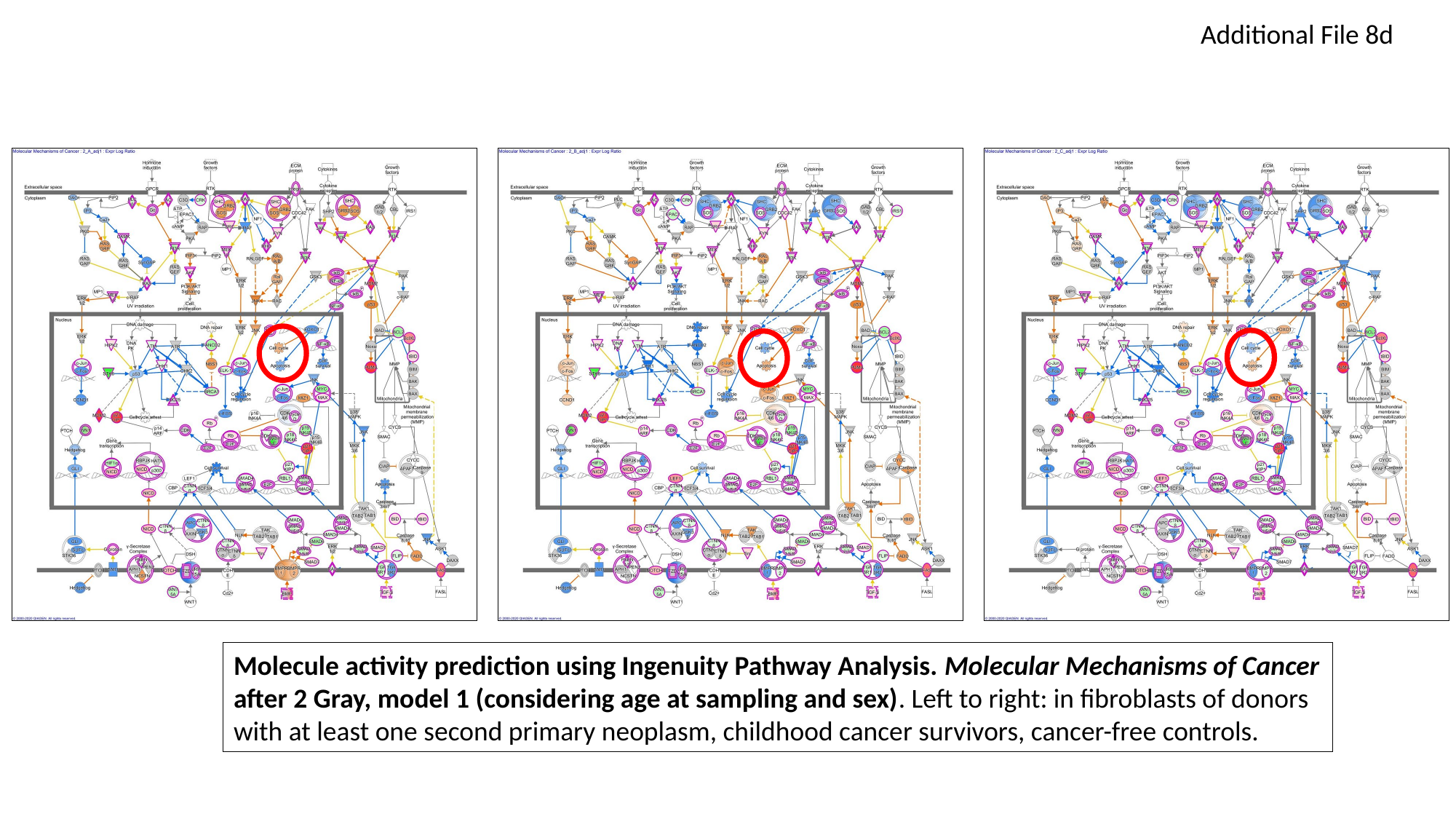

Additional File 8d
Molecule activity prediction using Ingenuity Pathway Analysis. Molecular Mechanisms of Cancer after 2 Gray, model 1 (considering age at sampling and sex). Left to right: in fibroblasts of donors with at least one second primary neoplasm, childhood cancer survivors, cancer-free controls.

## Slide 7
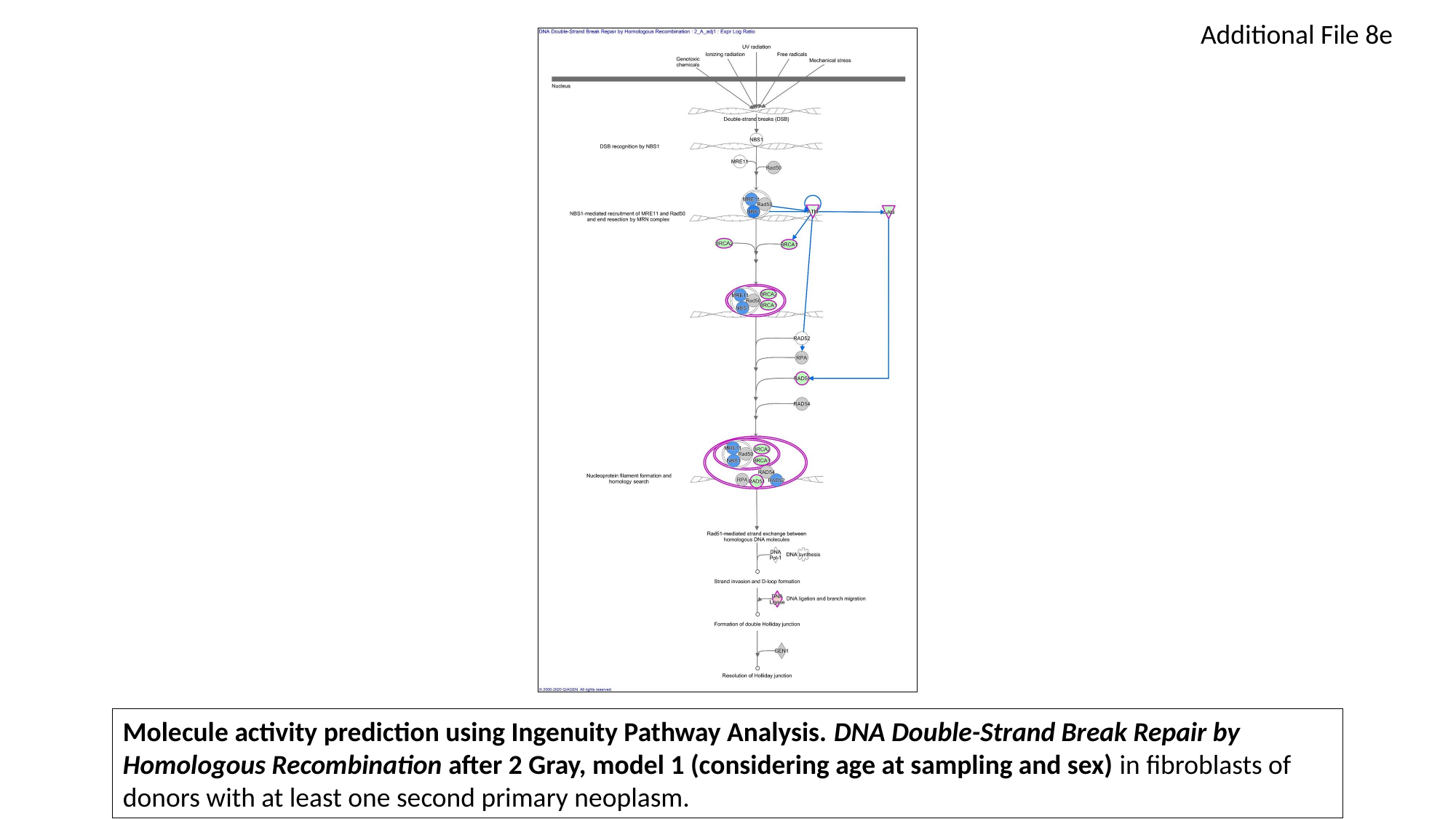

Additional File 8e
Molecule activity prediction using Ingenuity Pathway Analysis. DNA Double-Strand Break Repair by Homologous Recombination after 2 Gray, model 1 (considering age at sampling and sex) in fibroblasts of donors with at least one second primary neoplasm.

## Slide 8
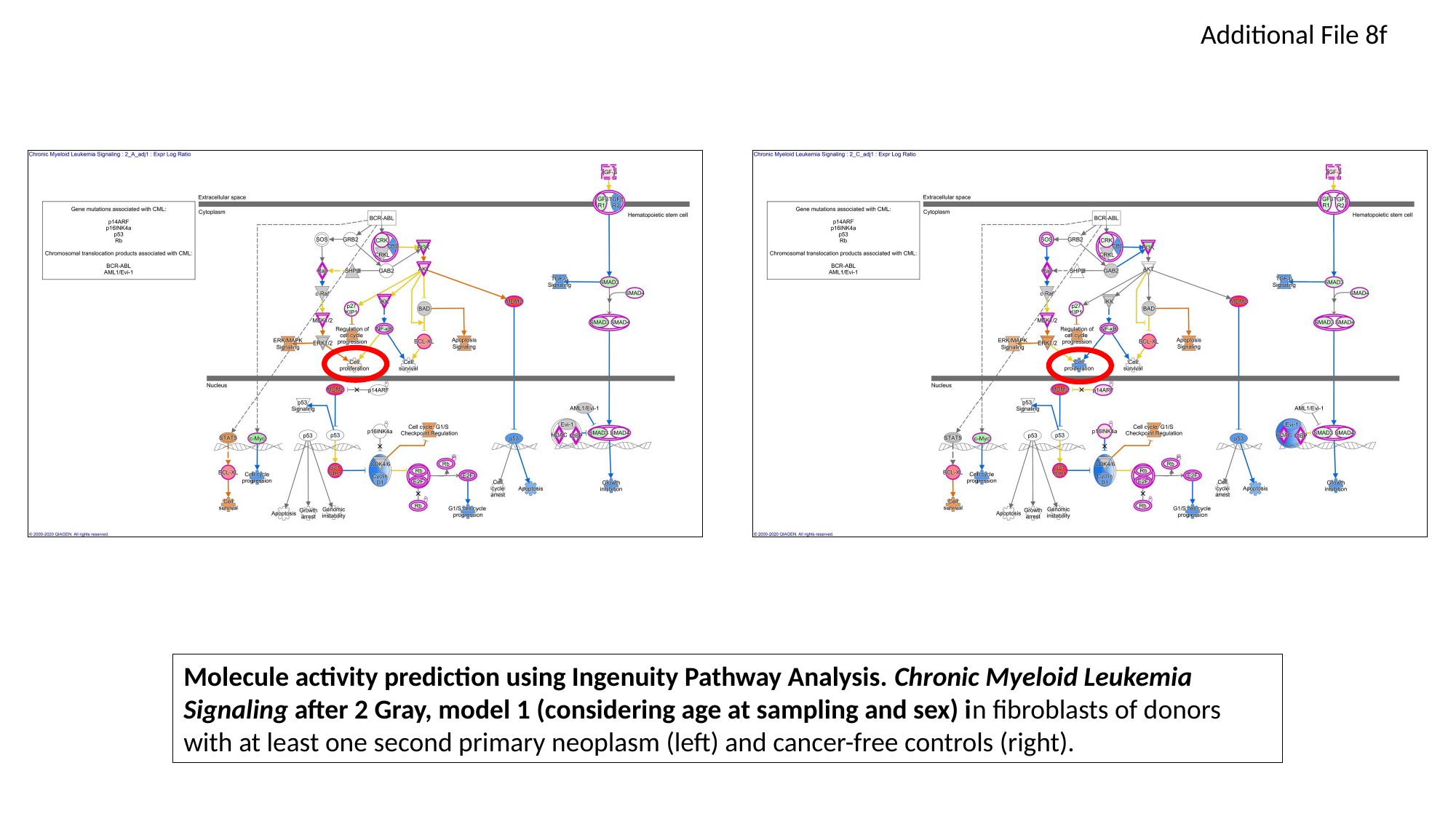

Additional File 8f
Molecule activity prediction using Ingenuity Pathway Analysis. Chronic Myeloid Leukemia Signaling after 2 Gray, model 1 (considering age at sampling and sex) in fibroblasts of donors with at least one second primary neoplasm (left) and cancer-free controls (right).

## Slide 9
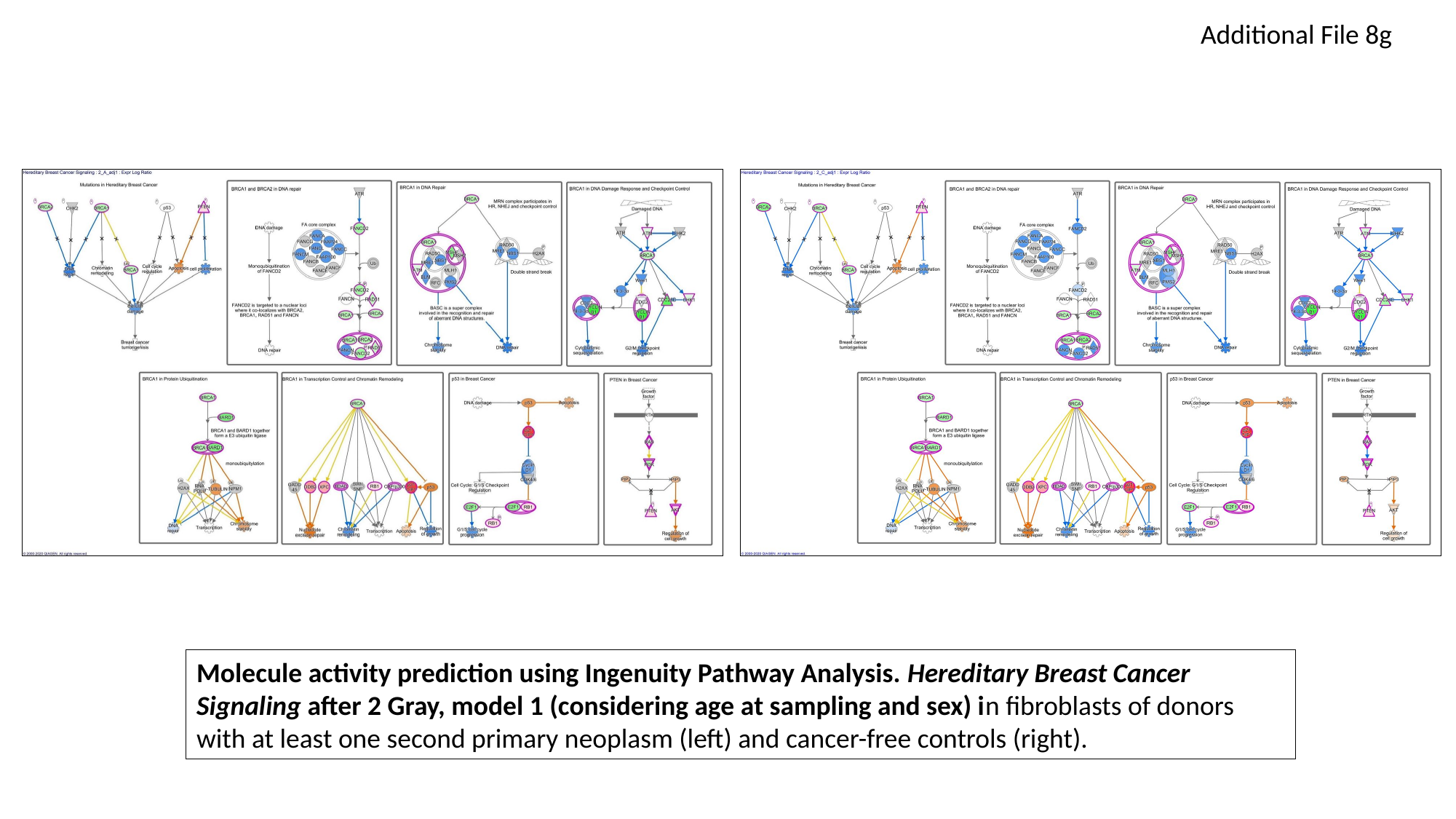

Additional File 8g
Molecule activity prediction using Ingenuity Pathway Analysis. Hereditary Breast Cancer Signaling after 2 Gray, model 1 (considering age at sampling and sex) in fibroblasts of donors with at least one second primary neoplasm (left) and cancer-free controls (right).

## Slide 10
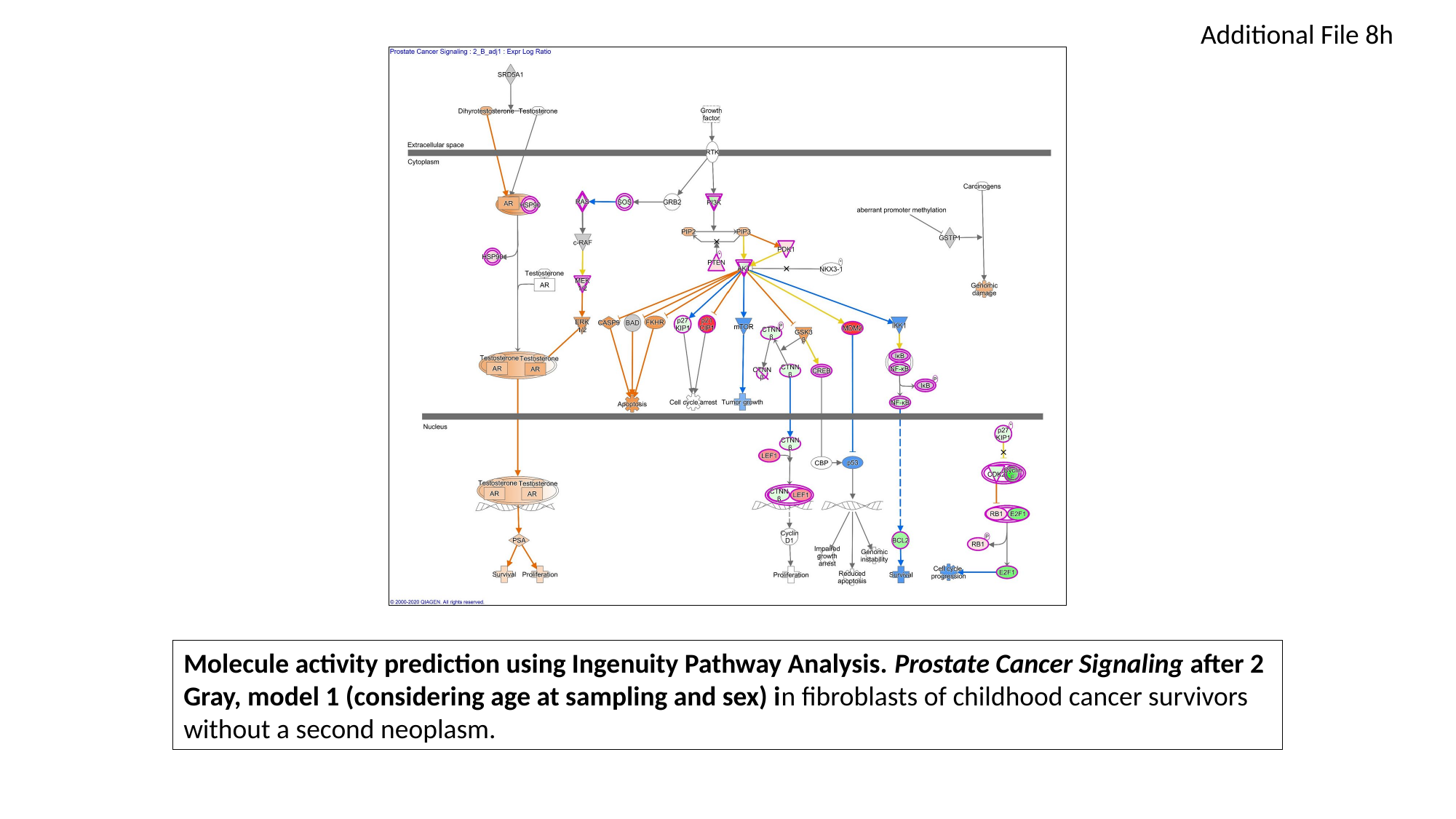

Additional File 8h
Molecule activity prediction using Ingenuity Pathway Analysis. Prostate Cancer Signaling after 2 Gray, model 1 (considering age at sampling and sex) in fibroblasts of childhood cancer survivors without a second neoplasm.
