# Supplementary material for: Radiation-response in primary fibroblasts of long-term survivors of childhood cancer with and without second primary neoplasms: the KiKme study
Source: Mol Med. 2022 Sep 6;28:105. doi: 10.1186/s10020-022-00520-6 (PMC9450413; doi:10.1186/s10020-022-00520-6)
Supplement: Supplementary file 9 — Additional file 9. Heat map of genes in relevant molecular networks. Heat map showing all genes and their respective log2 fold-change that were associated with molecular networks with a network score > 10 in any of the three donor groups in the differential gene expression data after exposure to 0.05 or 2 Gray. Model 1 considers age at sampling and sex; model 2 considers age at sampling, sex, age at and year of first diagnosis, and tumor type (not used with data from cancer-free controls). Tiles with black frames show genes that were part of the top networks for that dose/group/model combination. N0 = fibroblasts of cancer-free controls, N1 = fibroblasts of childhood-cancer survivors, N2+ = fibroblasts of childhood-cancer survivors with at least one second primary neoplasm. [file 10020_2022_520_MOESM9_ESM.docx]

**Additional File 9:** Heat map showing all genes and their respective log_2_ fold-change that were associated to molecule networks with a network score > 10 in any of the three donor groups in the differential gene expression data after exposure to 0.05 or 2 Gray. Model 1 considers age at sampling and sex; model 2 considers age at sampling, sex, age at and year of first diagnosis, and tumor type (not used with data from cancer-free controls). Tiles with black frames show genes that were part of the top networks for that dose/group/model combination. N0 = fibroblasts of cancer-free controls, N1 = fibroblasts of childhood-cancer survivors, N2+ = fibroblasts of childhood-cancer survivors with at least one second primary neoplasm.
