# Supplementary material for: Radiation-response in primary fibroblasts of long-term survivors of childhood cancer with and without second primary neoplasms: the KiKme study
Source: Mol Med. 2022 Sep 6;28:105. doi: 10.1186/s10020-022-00520-6 (PMC9450413; doi:10.1186/s10020-022-00520-6)
Supplement: Supplementary file 10 — Additional file 10. Results of GO over-representation analyses in gene expression models with interaction terms for donor group. AF10a. Heat maps displaying p-value and log2 fold-change of the top 5 genes per radiation dose, the direction of log2 fold-change per group-wise comparison for expression models including group-dose interaction (with regard to p-value). Log2 fold-change values can be found inside the tiles. Only the results for model 1 (considering age at sampling and sex) are displayed. N0 = fibroblasts of cancer-free controls, N1 = fibroblasts of donors with a first primary neoplasm, N2+ = fibroblasts of donors with at least one second primary neoplasm. * p-value < 0.05, ** p-value < 0.01, *** p-value < 0.001 (adjusted for false discovery rate). AF10b. Tree maps displaying clustered top gene ontology terms for the top 50 genes (with regard to p-value) for expression models including group-dose interaction (with regard to p-value) for each group-wise comparison. Only the results for model 1 (considering age and sex) are displayed. N0 = fibroblasts of cancer-free controls, N1 = fibroblasts of donors with a first primary neoplasm, N2+ = fibroblasts of donors with at least one second primary neoplasm. [file 10020_2022_520_MOESM10_ESM.pptx]

## Slide 1
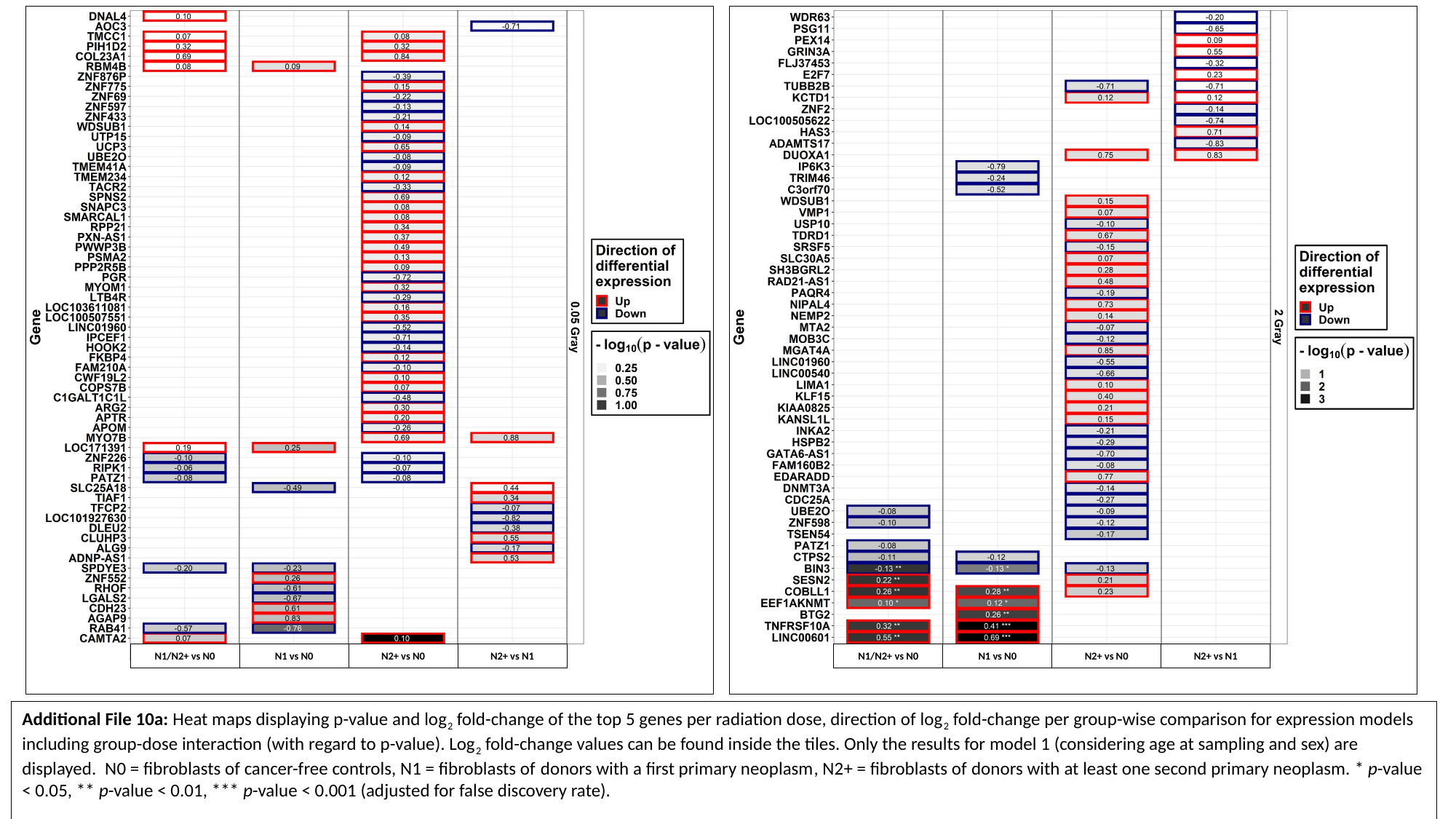

N1/N2+ vs N0
N1 vs N0
N2+ vs N0
N2+ vs N1
N1/N2+ vs N0
N1 vs N0
N2+ vs N0
N2+ vs N1
Additional File 10a: Heat maps displaying p-value and log2 fold-change of the top 5 genes per radiation dose, direction of log2 fold-change per group-wise comparison for expression models including group-dose interaction (with regard to p-value). Log2 fold-change values can be found inside the tiles. Only the results for model 1 (considering age at sampling and sex) are displayed. N0 = fibroblasts of cancer-free controls, N1 = fibroblasts of donors with a first primary neoplasm, N2+ = fibroblasts of donors with at least one second primary neoplasm. * p-value < 0.05, ** p-value < 0.01, *** p-value < 0.001 (adjusted for false discovery rate).

## Slide 2
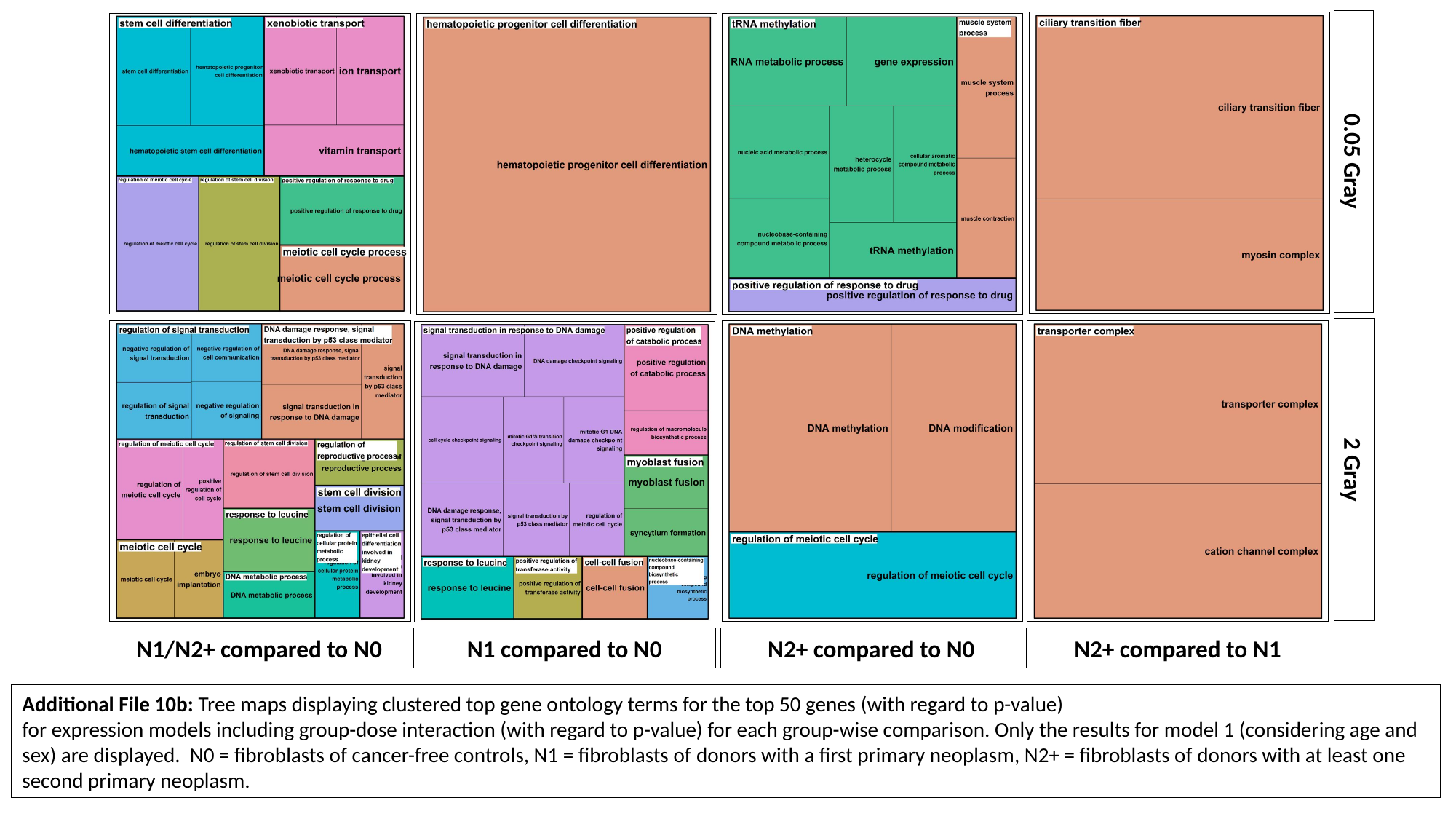

0.05 Gray
2 Gray
N1/N2+ compared to N0
N1 compared to N0
N2+ compared to N0
N2+ compared to N1
Additional File 10b: Tree maps displaying clustered top gene ontology terms for the top 50 genes (with regard to p-value)
for expression models including group-dose interaction (with regard to p-value) for each group-wise comparison. Only the results for model 1 (considering age and sex) are displayed. N0 = fibroblasts of cancer-free controls, N1 = fibroblasts of donors with a first primary neoplasm, N2+ = fibroblasts of donors with at least one second primary neoplasm.
