# Supplementary material for: Radiation-response in primary fibroblasts of long-term survivors of childhood cancer with and without second primary neoplasms: the KiKme study
Source: Mol Med. 2022 Sep 6;28:105. doi: 10.1186/s10020-022-00520-6 (PMC9450413; doi:10.1186/s10020-022-00520-6)
Supplement: Supplementary file 11 — Additional file 11. Results of differential gene expression and pathway analyses stratified by sex. AF11a. Bar charts showing the number and proportion of up- and downregulated genes stratified by dose, donor group, and sex. Differentially expressed genes were computed comparing irradiated to sham-irradiated fibroblasts after exposure to 0.05 and 2 Gray (false discovery rate adjusted p-value < 0.05), considering age at sampling. N0 = fibroblasts of cancer-free controls, N1 = fibroblasts of childhood-cancer survivors, N2+ = fibroblasts of childhood-cancer survivors with at least one second primary neoplasm; * p-value < 0.05, ** p-value < 0.01, *** p-value < 0.001. AF11b. Upper Half: Volcano plots showing differential expression after 0.05 Gray stratified by donor group and sex, considering age at sampling. Lower Half: Heat map showing top five ranking genes with regard to p-value per group considering age at sampling. N0 = fibroblasts of cancer-free controls, N1 = fibroblasts of childhood-cancer survivors, N2+ = fibroblasts of childhood-cancer survivors with at least one second primary neoplasm; * p-value < 0.05, ** p-value < 0.01, *** p-value < 0.001. N.S. = not significant (p-value > 0.05). AF11c. Upper Half: Volcano plots showing differential expression after 2 Gray stratified by donor group and sex, considering age at sampling. Lower Half: Heat map showing top five ranking genes with regard to p-value per group considering age at sampling. N0 = fibroblasts of cancer-free controls, N1 = fibroblasts of childhood-cancer survivors, N2+ = fibroblasts of childhood-cancer survivors with at least one second primary neoplasm; * p-value < 0.05, ** p-value < 0.01, *** p-value < 0.001. N.S. = not significant (p-value > 0.05). AF11d. Overview of affected (false discovery rate adjusted p-value < 0.05) and (in-) activated pathways (|z|≥ 2), predicted upstream effectors, downstream biofunctions and diseases, and observed molecular networks after irradiation with a low (0.05 Gray [file 10020_2022_520_MOESM11_ESM.docx]

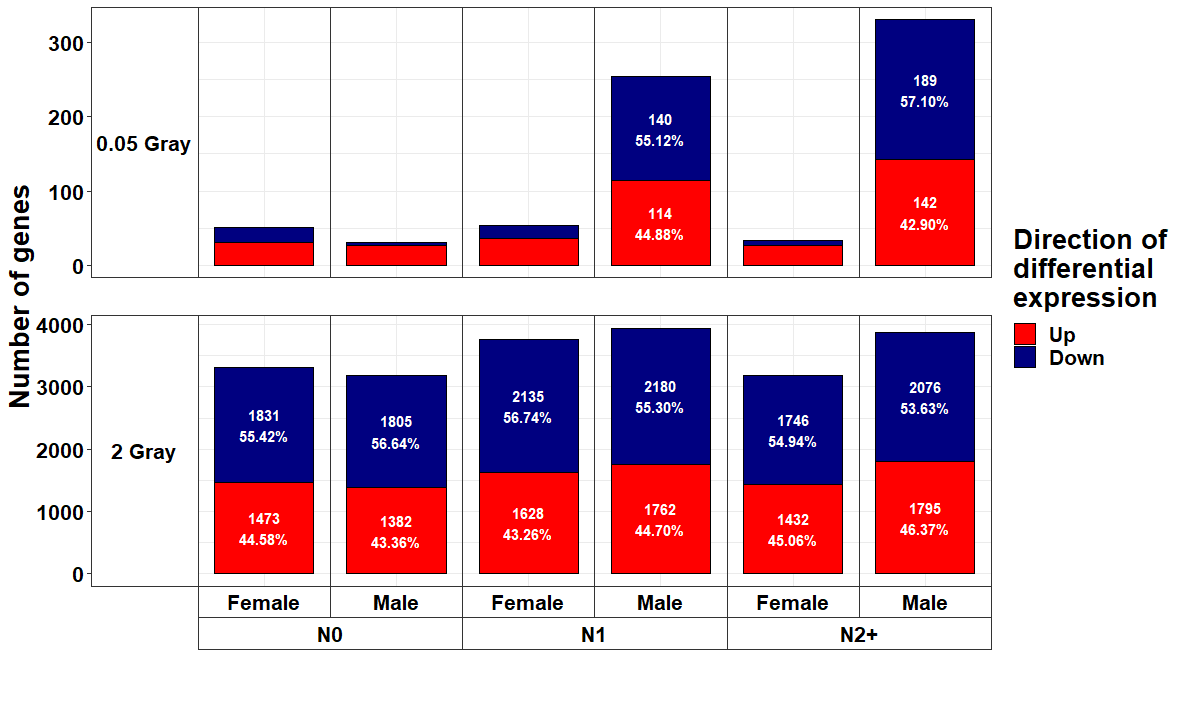


**Additional File 11a:** Bar charts showing the number and proportion of up- and downregulated genes stratified by dose, donor group, and sex. Differentially expressed genes were computed comparing irradiated to sham-irradiated fibroblasts after exposure to 0.05 and 2 Gray (false discovery rate adjusted p < 0.05), considering age at sampling. N0 = fibroblasts of cancer-free controls, N1 = fibroblasts of childhood-cancer survivors, N2+ = fibroblasts of childhood-cancer survivors with at least one second primary neoplasm; * p-value < 0.05, ** p-value < 0.01, *** p-value < 0.001.





**Additional File 11b. Upper Half:** Volcano plots showing differential expression after 0.05 Gray stratified by donor group and sex, considering age at sampling. **Lower Half:** Heat map showing top five ranking genes with regard to p-value per group considering age at sampling. N0 = fibroblasts of cancer-free controls, N1 = fibroblasts of childhood-cancer survivors, N2+ = fibroblasts of childhood-cancer survivors with at least one second primary neoplasm; * p-value < 0.05, ** p-value < 0.01, *** p-value < 0.001. N.S. = not significant (p-value > 0.05).





**Additional File 11c. Upper Half:** Volcano plots showing differential expression after 2 Gray stratified by donor group and sex, considering age at sampling. **Lower Half:** Heat map showing top five ranking genes with regard to p-value per group considering age at sampling. N0 = fibroblasts of cancer-free controls, N1 = fibroblasts of childhood-cancer survivors, N2+ = fibroblasts of childhood-cancer survivors with at least one second primary neoplasm; * p-value < 0.05, ** p-value < 0.01, *** p-value < 0.001. N.S. = not significant (p-value > 0.05).

**

**

**Additional File 11d:** Overview of affected (false discovery rate adjusted p-value < 0.05) and (in-) activated pathways (|z| ≥ 2), predicted upstream effectors, downstream biofunctions and diseases, and observed molecule networks after irradiation with a low (0.05 Gray) or a high dose (2 Gray) ordered by p-value, stratified by sex and considering age at sampling. For molecule networks, the network score instead of a p-value and no z-score was calculated by *Ingenuity Pathway Analysis*. N0 = fibroblasts of cancer-free controls, N1 = fibroblasts of childhood-cancer survivors, N2+ = fibroblasts of childhood-cancer survivors with at least one second primary neoplasm; *p-value < 0.05, ** p-value < 0.01, ***p-value < 0.001.





**Additional File 11e:** Heat map showing all pathways from *Ingenuity Pathway Analysis* that were significantly enriched in one of the three donor groups (false discovery rate adjusted p-value < 0.05) using results from the differential gene expression data stratified by sex and considering age at sampling after exposure to 2 Gray. N0 = fibroblasts of cancer-free controls, N1 = fibroblasts of childhood-cancer survivors, N2+ = fibroblasts of childhood-cancer survivors with at least one second primary neoplasm; *p-value < 0.05, ** p-value < 0.01, ***p-value < 0.001.
