# Supplementary material for: Radiation-response in primary fibroblasts of long-term survivors of childhood cancer with and without second primary neoplasms: the KiKme study
Source: Mol Med. 2022 Sep 6;28:105. doi: 10.1186/s10020-022-00520-6 (PMC9450413; doi:10.1186/s10020-022-00520-6)
Supplement: Supplementary file 12 — Additional file 12. Results of differential gene expression and pathway analyses after exclusion of self-reported non-Caucasian donors. AF12a. Bar charts showing the proportion of up- and downregulated genes stratified by dose, and donor group after exclusion of self-reported non-Caucasian participants. Differentially expressed genes in irradiated compared to sham-irradiated fibroblasts from N0 = fibroblasts of cancer-free controls, N1 = fibroblasts of childhood-cancer survivors, N2+ = fibroblasts of childhood-cancer survivors with at least one second primary neoplasm. Model 1 considers age at sampling and sex; model 2 considers age at sampling, sex, as well as age at and year of diagnosis of the first neoplasm, and tumor type. AF12b. Overview of affected (false discovery rate adjusted p-value < 0.05) and (in-) activated pathways (|z|≥ 2), predicted upstream effectors, downstream biofunctions and diseases, and observed molecular networks after irradiation with a low (0.05 Gray) or a high dose (2 Gray) ordered by p-value, after exclusion of self-reported non-Caucasian participants. For molecular networks, the network score instead of a p-value and no z-score was calculated by Ingenuity Pathway Analysis. Model 1 considers age at sampling and sex. N0 = fibroblasts of cancer-free controls, N1 = fibroblasts of childhood-cancer survivors, N2+ = fibroblasts of childhood-cancer survivors with at least one second primary neoplasm; * p-value < 0.05, ** p-value < 0.01, *** p-value < 0.001. AF12c. Heat map showing all pathways from Ingenuity Pathway Analysis that were significantly enriched in one of the three donor groups (false discovery rate adjusted p-value < 0.05) in the differential gene expression data after exclusion of self-reported non-Caucasian participants after exposure to 2 Gray. Model 1 considers age at sampling and sex. N0 = fibroblasts of cancer-free controls, N1 = fibroblasts of childhood-cancer survivors, N2+ = fibroblasts of childhood-cancer survivors with a [file 10020_2022_520_MOESM12_ESM.docx]

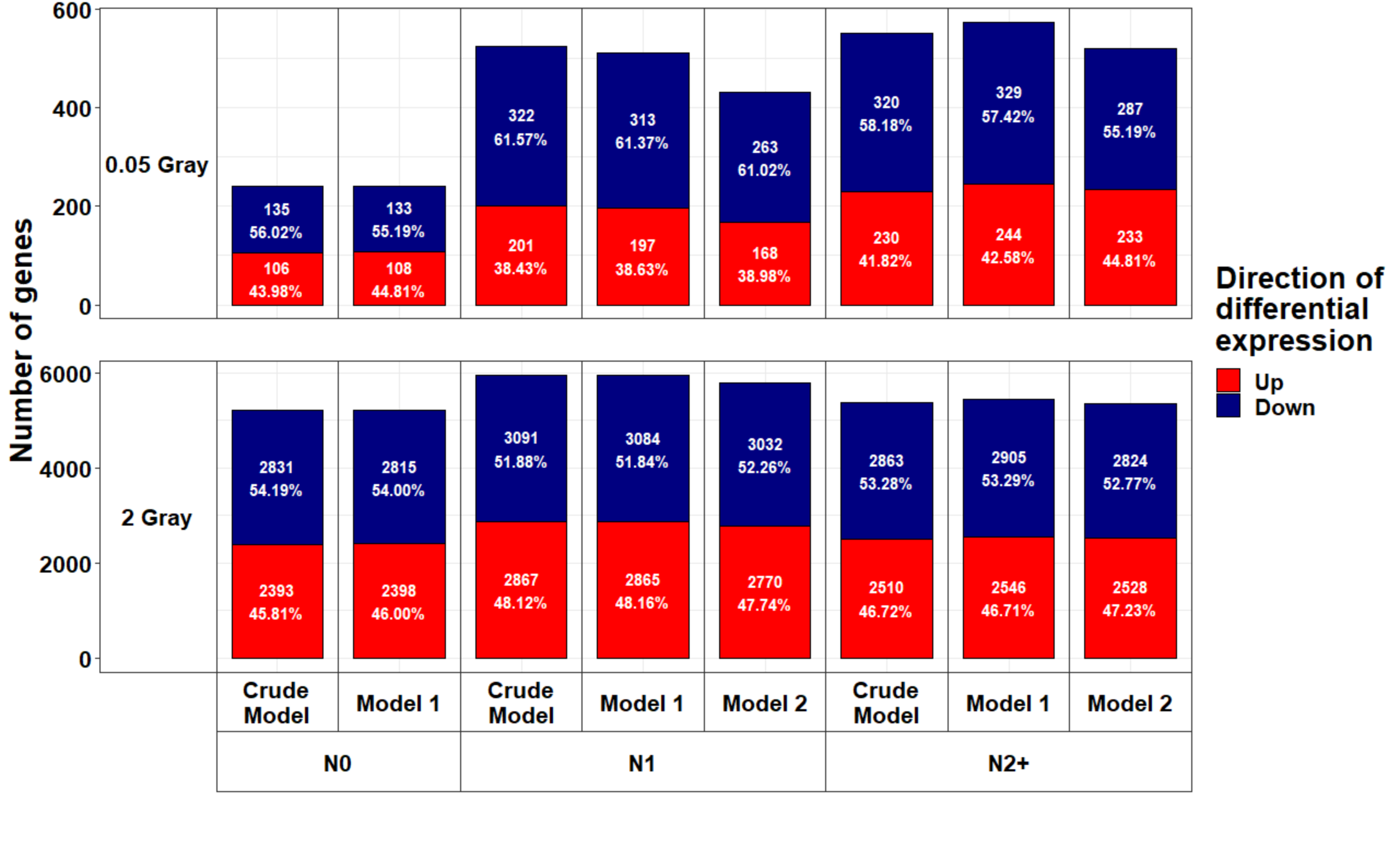
 **Additional File 12a:** Bar charts showing the proportion of up- and downregulated genes stratified by dose, donor group after exclusion of self-reported non-Caucasian participants. Differentially expressed genes in irradiated compared to sham-irradiated fibroblasts from N0 = fibroblasts of cancer-free controls, N1 = fibroblasts of childhood-cancer survivors, N2+ = fibroblasts of childhood-cancer survivors with at least one second primary neoplasm. Model 1 considers age at sampling and sex; model 2 considers age at sampling, sex, as well as age at and year of diagnosis of the first neoplasm, and tumor type.

**
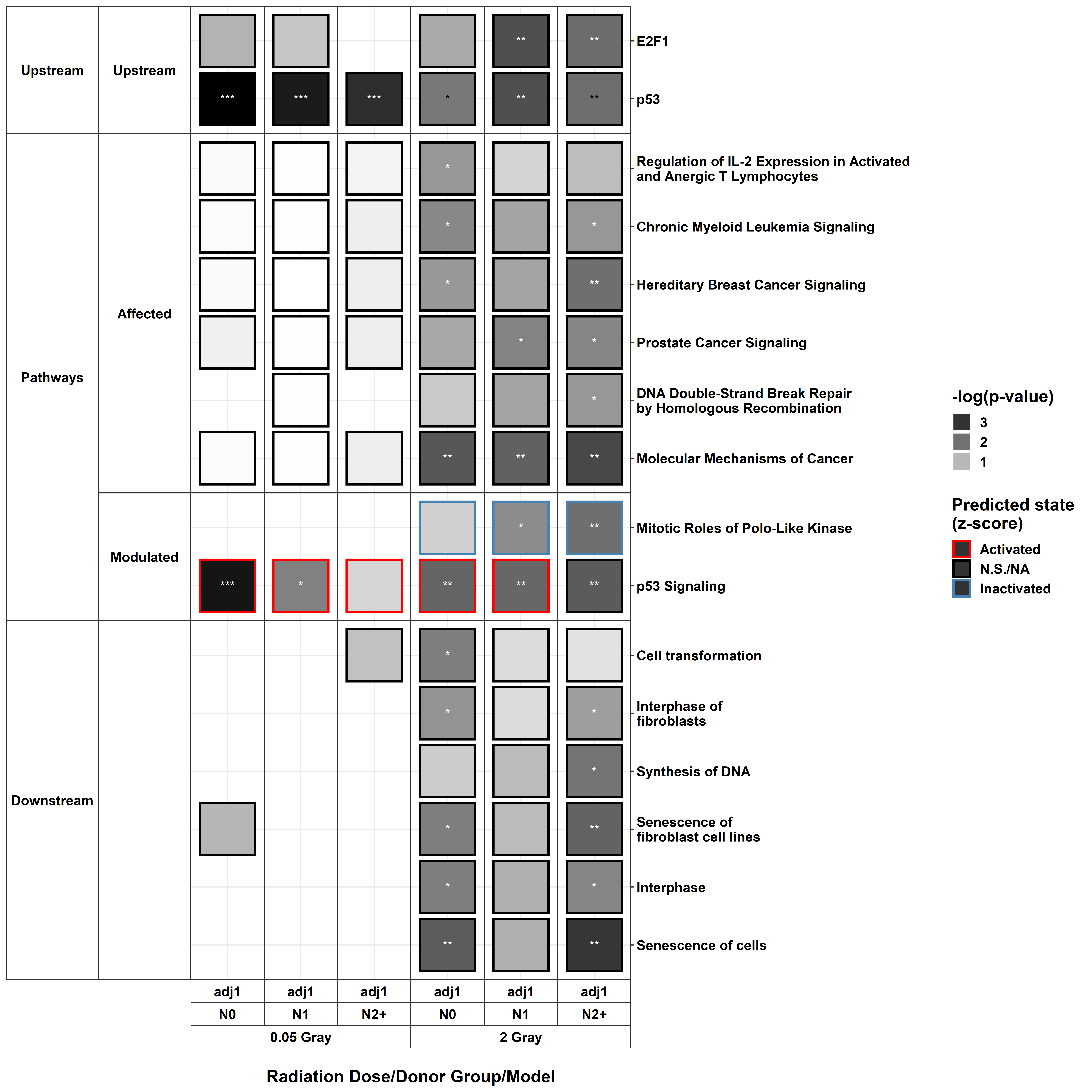
Additional File 12b:** Overview of affected (false discovery rate adjusted p < 0.05) and (in-) activated pathways (|z| ≥ 2), predicted upstream effectors, downstream biofunctions and diseases, and observed molecule networks after irradiation with a low (0.05 Gray) or a high dose (2 Gray) ordered by p-value, after exclusion of self-reported non-Caucasian participants. For molecule networks, the network score instead of a p-value and no z-score was calculated by *Ingenuity Pathway Analysis*. Model 1 considers age at sampling and sex. N0 = fibroblasts of cancer-free controls, N1 = fibroblasts of childhood-cancer survivors, N2+ = fibroblasts of childhood-cancer survivors with at leasts one second primary neoplasm; * p < 0.05, ** p < 0.01, *** p < 0.001.

**

**

**Additional File 12c:** Heat map showing all pathways from *Ingenuity Pathway Analysis* that were significantly enriched in one of the three donor groups (false discovery rate adjusted p < 0.05) in the differential gene expression data after exclusion of self-reported non-Caucasian participants after exposure to 2 Gray. Model 1 considers age at sampling and sex. N0 = fibroblasts of cancer-free controls, N1 = fibroblasts of childhood-cancer survivors, N2+ = fibroblasts of childhood-cancer survivors with at leasts one second primary neoplasm; * p < 0.05, ** p < 0.01, *** p < 0.001.
