# Supplementary material for: Radiation-response in primary fibroblasts of long-term survivors of childhood cancer with and without second primary neoplasms: the KiKme study
Source: Mol Med. 2022 Sep 6;28:105. doi: 10.1186/s10020-022-00520-6 (PMC9450413; doi:10.1186/s10020-022-00520-6)
Supplement: Supplementary file 13 — Additional file 13. Results of pathway analysis after exclusion of genes with less than 20% up- or downregulation.Overview of affected (false discovery rate adjusted at p-value < 0.05) and (in-) activated pathways (|z| ≥ 2), predicted upstream effectors, downstream biofunctions and diseases after irradiation with a low (0.05 Gray) or a high dose (2 Gray) ordered by p-value.* p--value < 0.05, ** p--value < 0.01, *** p--value < 0.001. Model 1: considering age at sampling and sex, model 2: considering age at sampling, sex, age at and year of diagnosis of the first neoplasm, and tumor type (not used with data from N0), N0 = fibroblasts of cancer-free controls, N1 = fibroblasts of childhood-cancer survivors, N2+ = fibroblasts of childhood-cancer survivors with at least one second primary neoplasm. [file 10020_2022_520_MOESM13_ESM.docx]

**

**

**Additional File 13. Results of *Ingenuity Pathway Analysis* using only genes with a log_2_fold-change > |0.25|:** Overview of affected (false discovery rate adjusted at *p-value* < 0.05) and (in-) activated pathways (|z| ≥ 2), predicted upstream effectors, downstream biofunctions and diseases after irradiation with a low (0.05 Gray) or a high dose (2 Gray) ordered by *p-*value.* *p-*value < 0.05, ** *p-*value < 0.01, *** *p-*value < 0.001. Model 1: considering age at sampling and sex, model 2: considering age at sampling, sex, age at and year of diagnosis of the first neoplasm, and tumor type (not used with data from N0), N0 = fibroblasts of cancer-free controls, N1 = fibroblasts of childhood-cancer survivors, N2+ = fibroblasts of childhood-cancer survivors with at least one second primary neoplasm.
